# Non-commutative Calculus, Optimal Transport and Functional Inequalities in Dissipative Quantum Systems

**DOI:** 10.1007/s10955-019-02434-w

**Published:** 2019-11-27

**Authors:** Eric A. Carlen, Jan Maas

**Affiliations:** 1 Department of Mathematics, Hill Center, Rutgers University, 110 Frelinghuysen Road, Piscataway, NJ 08854-8019 USA; 2 Institute of Science and Technology Austria (IST Austria), Am Campus 1, 3400 Klosterneuburg, Austria

**Keywords:** Non-commutative optimal transport, Functional inequalities, Lindblad equation, Gradient flow

## Abstract

We study dynamical optimal transport metrics between density matrices associated to symmetric Dirichlet forms on finite-dimensional $C^*$-algebras. Our setting covers arbitrary skew-derivations and it provides a unified framework that simultaneously generalizes recently constructed transport metrics for Markov chains, Lindblad equations, and the Fermi Ornstein–Uhlenbeck semigroup. We develop a non-nommutative differential calculus that allows us to obtain non-commutative Ricci curvature bounds, logarithmic Sobolev inequalities, transport-entropy inequalities, and spectral gap estimates.

## Introduction

In the context of diffusion semigroups, a great deal of recent progress has been made based on two different gradient flow interpretations of the heat flow, namely as

1. The gradient flow of the Dirichlet energy in $L^2$;
2. The gradient flow of the Boltzmann entropy in the space of probability measures endowed with the 2-Kantorovich metric.

In this paper we study the analogs of (1) and (2) for non-commutative probability, in the setting von Neumann algebras, and we establish the equivalence of (1) and (2) in this setting. This naturally involves the construction of non-commutative analogs of the 2-Kantorovich metric, a topic that was investigated in our earlier papers [8, 10] and in the independent work [34, 36]. Recently the subject received the attention of a number of authors; see [11, 12] for noncommutative transport metrics, [22, 45, 46] for functional inequalities, and [28, 48] for results in infinite dimensions. We refer to [6, 23] for different non-commutative variants of the 2-Kantorovich metric in other contexts.

Our focus in this paper is on developing the relations between (1) and (2) in the non-commutative setting with the aim of proving functional inequalities relevant to the study of the rate of approach to equilibrium for quantum Markov semigroups, in close analogy with what has been accomplished along these lines in the classical setting in recent years.

In order not to obscure the main ideas we shall work in a finite-dimensional setting and postpone the infinite-dimensional extension to a future work. The finite-dimensional case is of direct interest in quantum information theory, and the essential aspects of our new results are interesting even in this setting where they can be explained to a wider audience that is not thoroughly familiar with the Tomita–Takesaki theory. We now briefly describe the content of the paper. Any unfamiliar terminology is explained in the next subsection, but hopefully many readers will not need to look ahead.

The central object of study in this paper is a quantum Markov semigroup (QMS) $(\mathscr {P}_t)_{t>0}$ on $\mathcal {A}$, a finite-dimensional $C^*$-algebra containing the identity ${\mathbf{1}}$. That is, for each *t*, $\mathscr {P}_t{\mathbf{1}}= {\mathbf{1}}$ and $\mathscr {P}_t$ is completely positive. The generators ${\mathscr {L}}$ of such semigroups have been characterized in [24, 31].

We are concerned with the case in which there is a unique faithful invariant state $\sigma$ for the dual semigroup; i.e., $\mathscr {P}_t^\dagger \sigma = \sigma$ for all *t*. The paper [47] is an excellent source for the physical context and makes it clear that assuming that the invariant state $\sigma$ is tracial, which we do not do, would preclude a great many physical applications. Let ${{\mathfrak {P}}}_+$ denote the space of faithful states. We would like to know, for instance, when there is a Riemannian metric *g* on ${{\mathfrak {P}}}_+$ such that the flow on ${{\mathfrak {P}}}_+$ given by the dual semigroup $(\mathscr {P}_t^\dagger )_{t>0}$ is the gradient flow driven by the relative entropy functional ${{\,\mathrm{Ent}\,}}_\sigma (\rho ) = {{\,\mathrm{Tr}\,}}[\rho (\log \rho - \log \sigma )]$ with respect to the Riemannian metric. In [10, 36], it is shown that when each $\mathscr {P}_t$ is self-adjoint with respect to the Gelfand-Naimark-Segal (GNS) inner product induced on $\mathcal {A}$ by $\sigma$, this is the case. We constructed the metric using ideas from optimal mass transport, and showed that, as in the classical case, the framework provided an efficient means for proving functional inequalities. This has been taken up and further developed by other authors, in particular Rouzé and Datta [45, 46]. As in the classical case, Ricci curvature bounds are essential for the framework to be used to obtain functional inequalities. As shown in [10, 46], once one has Ricci curvature bounds, a host of functional inequalities follow. A central problem then is to prove such bounds. A main contribution of the present paper is a flexible framework for doing this. It turns out that there are many ways to write a given QMS generator $\mathscr {L}$ (that is self-adjoint in the GNS sense) in “divergence form” for non-commutative derivatives. Each of the different ways of doing this can be associated to a Riemannian metric on ${{\mathfrak {P}}}_+$. Different ways of writing $\mathscr {L}$ in divergence form may have advantages over others, for example in proving Ricci curvature bounds. Hence it is important to have as much flexibility here as possible. We shall use this flexibility to give new examples in which we can obtain sharp Ricci curvature bounds. The machinery is useful for other functionals and other flows; the methods of this paper are not by any means restricted to gradient flow for relative entropy, despite our focus on this example here in the introduction.

An interesting problem remains: For each way of writing $\mathscr {L}$ in divergence form, we have a Riemannian metric. The formulas are different, but in principle, all of the metrics might be the same. That is, they might all be determined by $\mathscr {L}$, and not the particular way of writing in divergence form, even though doing this one way or another may facilitate certain computations.

The problem of writing QMS as gradient flow for the relative entropy was also taken up independently by Mittnenzweig and Mielke [36], and although their framework is somewhat different, their approach also works in the case that each $\mathscr {P}_t$ is self-adjoint with respect to the GNS inner product induced on $\mathcal {A}$ by $\sigma$. Here, we shall show that if $(\mathscr {P}_t)_{t\ge 0}$ can be written as gradient flow for ${{\,\mathrm{Ent}\,}}_\sigma$ with respect to some continuously differentiable Riemannian metric, then each $\mathscr {P}_t$ is necessarily self-adjoint with respect to another inner product associated to $\sigma$, the Boguliobov-Kubo-Mori (BKM) inner product. As we show, the class of QMS with this self-adjointness property is strictly larger than the class of QMS with the GNS self-adjointness property. Thus, there is at present an interesting gap between the known necessary condition for the construction of the Riemannian metric, and the known sufficient condition. Of course, in the classical setting, the two notions of self-adjointness coincide, and one has a pleasing characterization of reversible Markov chains in terms of gradient flow [15].

### Notation

Let $\mathcal {A}$ be finite-dimensional $C^*$-algebra containing the identity ${\mathbf{1}}$. In the finite-dimensional setting, all topologies one might impose on $\mathcal {A}$ are equivalent, and $\mathcal {A}$ is also a von Neumann algebra. In particular, it is generated by the projections it contains. We may regard any such algebra as a $*$-subalgebra of ${{\mathbb {M}}}_n({{\mathbb {C}}})$, the set of all complex $n \times n$ matrices. Let $\mathcal {A}_h$ be the subset of hermitian elements in $\mathcal {A}$, and let $\mathcal {A}_+ \subseteq \mathcal {A}$ denote the class of elements that are positive definite (i.e., ${{\,\mathrm{sp}\,}}(A) \subseteq (0,\infty )$ for $A \in \mathcal {A}_+$. For $\mathcal {A}= {{\mathbb {M}}}_n({{\mathbb {C}}})$ we write $\mathcal {A}_+ = {{\mathbb {M}}}_n^+({{\mathbb {C}}})$.

Throughout this section we fix a positive linear functional $\tau$ on $\mathcal {A}$ that is *tracial* (i.e., $\tau [AB] = \tau [BA]$ for all $A, B \in \mathcal {A}$) and *faithful* (i.e., $A=0$ whenever $\tau [A^*A] =0$). Under these assumptions, $\tau$ induces a scalar product on $\mathcal {A}$ given by $\langle {A,B}\rangle _{L^2(\mathcal {A},\tau )} = \tau [A^* B]$ for $A, B \in \mathcal {A}$. In our applications, $\tau$ will often be the usual trace ${{\,\mathrm{Tr}\,}}$ on ${{\mathbb {M}}}_n({{\mathbb {C}}})$ in which case the scalar product is the Hilbert–Schmidt scalar product, but it will be useful to include different situations, e.g., the trace induced by a non-uniform probability measure on a finite set.

A *state* on $\mathcal {A}$ is a positive linear functional $\varphi$ on $\mathcal {A}$ such that $\varphi ({\mathbf{1}}) =1$. If $\varphi$ is a state, there is a uniquely determined $\sigma \in \mathcal {A}$ such that $\varphi (A) = \tau [\sigma A]$ for all $A \in \mathcal {A}$. Note that $\sigma$ is a *density matrix*; i.e., it is positive semidefinite and $\tau [\sigma ] =1$. Let ${{\mathfrak {P}}}(\mathcal {A})$ denote the set of density matrices. We write ${{\mathfrak {P}}}_+(\mathcal {A}) = \{ \rho \in {{\mathfrak {P}}}(\mathcal {A}) : \rho \text { is positive definite} \}$. We will simply write ${{\mathfrak {P}}}= {{\mathfrak {P}}}(\mathcal {A})$ and ${{\mathfrak {P}}}_+ = {{\mathfrak {P}}}_+(\mathcal {A})$ if the algebra $\mathcal {A}$ is clear from the context.

We always use $\dagger$ to denote the adjoint of a linear transformation on $\mathcal {A}$ with respect to the scalar product $\langle {\cdot , \cdot }\rangle _{L^2(\mathcal {A},\tau )}$. If $\mathscr {K}$ is such a linear transformation,

1.1 $$\begin{aligned} \langle A, \mathscr {K}B\rangle _{L^2(\mathcal {A},\tau )} = \langle \mathscr {K}^\dagger A , B\rangle _{L^2(\mathcal {A},\tau )} \ . \end{aligned}$$

Though we suppose no familiarity with the Tomita–Takesaki Theory of standard forms of von Neumann algebras, we will make use of the so-called modular and relative modular operators that arise there. In our setting, these operators have a simple direct definition:

#### Definition 1.1

(*The relative modular operator*) Let $\sigma ,\rho \in {{\mathfrak {P}}}_+$. The corresponding *relative modular operator* $\Delta _{\sigma ,\rho }$ is the linear transformation on $\mathcal {A}$ defined by

1.2 $$\begin{aligned} \Delta _{\sigma ,\rho }(A) = \sigma A \rho ^{-1}\ . \end{aligned}$$

The *modular operator* corresponding to $\sigma$, $\Delta _\sigma$, is defined by $\Delta _{\sigma } := \Delta _{\sigma ,\sigma }$.

Since $\langle B, \Delta _{\sigma ,\rho } A\rangle _{L^2(\mathcal {A},\tau )} = \tau [(\sigma ^{1/2}B\rho ^{-1/2})^*(\sigma ^{1/2}A\rho ^{-1/2})]$ for all $A,B\in \mathcal {A}$, the operator $\Delta _{\sigma ,\rho }$ is positive definite on $L^2(\mathcal {A},\tau )$. In case that $\tau$ is the restriction of the usual trace ${{\,\mathrm{Tr}\,}}$ to $\mathcal {A}\subseteq {{\mathbb {M}}}_n({{\mathbb {C}}})$, the operators $\sigma$ and $\rho$ are also positive density matrices in ${{\mathbb {M}}}_n({{\mathbb {C}}})$, and the same computations are valid for all $A,B\in {{\mathbb {M}}}_n({{\mathbb {C}}})$. We may regard $\Delta _\sigma$ as an operator on ${{\mathbb {M}}}_n({{\mathbb {C}}})$, equipped with the Hilbert–Schmidt inner product, and then, so extended, it is still positive definite.

We are interested in evolution equations on ${{\mathfrak {P}}}_+(\mathcal {A})$ that correspond to forward Kolmogorov equations for ergodic Markov processes satisfying a detailed balance condition, or in other words a reversibility condition, with respect to their unique invariant probability measure. Before presenting our results, we introduce the class of quantum Markov semigroups satisfying a detailed balance condition that are the focus of our investigation.

## Quantum Markov Semigroups with Detailed Balance

Let $\mathcal {A}\subseteq B(\mathscr {H})$ be a $C^*$-algebra of operators acting on a finite-dimensional Hilbert space $\mathscr {H}$. Let $\tau$ be a tracial and faithful positive linear functional on $\mathcal {A}$. A *quantum Markov semigroup* on $\mathcal {A}$ is a $C_0$-semigroup of operators $(\mathscr {P}_t)_{t \ge 0}$ acting on $\mathcal {A}$, satisfying

1. $$\mathscr {P}_t {\mathbf{1}}= {\mathbf{1}}$$;
2. $\mathscr {P}_t$ is *completely positive*, i.e., $\mathscr {P}_t \otimes I_{{{\mathbb {M}}}_n({{\mathbb {C}}})}$ is a positivity preserving operator on $\mathcal {A}\otimes {{\mathbb {M}}}_n({{\mathbb {C}}})$ for all $n \in {{\mathbb {N}}}$.

Note that (2) implies that $\mathscr {P}_{t}$ is *real*, i.e., $(\mathscr {P}_t A)^* = \mathscr {P}_t A^*$ for all $A \in \mathcal {A}$. Let $\mathscr {P}_t^\dagger$ be the Hilbert–Schmidt adjoint of $\mathscr {P}_t$ satisfying $\tau [A^* \mathscr {P}_t^\dagger B] = \tau [(\mathscr {P}_t A)^*B]$ for all $A, B \in \mathcal {A}$. It follows that $\mathscr {P}_t^\dagger$ is trace-preserving and completely positive.

It is well known [24, 31] that the generator $\mathscr {L}$ of the semigroup $\mathscr {P}_t = e^{t \mathscr {L}}$ can be written in *Lindblad form*

2.1 $$\begin{aligned} \mathscr {L}A&= i [{\widetilde{H}},A] + \sum _{j \in \mathcal {J}} V_j^* [A, V_j] + [V_j^* , A] V_j \ , \end{aligned}$$

2.2 $$\begin{aligned} \mathscr {L}^\dagger \rho&= - i [{\widetilde{H}},\rho ] + \sum _{j \in \mathcal {J}} [V_j, \rho V_j^*] + [V_j \rho , V_j^*] \ , \end{aligned}$$

where $\mathcal {J}$ is a finite index set, $V_j \in B(\mathscr {H})$ (not necessarily belonging to $\mathcal {A}$) for all $j \in \mathcal {J}$, and the Hamiltonian ${\widetilde{H}} \in B(\mathscr {H})$ is self-adjoint.

### Detailed Balance

The starting point of our investigations is the assumption that $(\mathscr {P}_t)_{t \ge 0}$ satisfies the condition of *detailed balance*.

In the commutative setting, if $P = (P_{ij})$ is the transition matrix of a Markov chain on $\{1,\ldots , n\}$ with invariant probability vector $\sigma$, we say that detailed balance holds if $\sigma _i P_{ij} = \sigma _j P_{ji}$ for all *i*, *j*. An analytic way to formulate this condition is that *P* is self-adjoint with respect to the weighted inner product on ${{\mathbb {C}}}^n$ given by $\langle {f,g}\rangle _\sigma = \sum _{j=1}^n \sigma _j \overline{f_j}g_j$.

In the quantum setting, with a reference density matrix $\sigma$ that is not a multiple of the identity, there are many candidates for such a weighted inner product. E.g., given $\sigma \in {{\mathfrak {P}}}_+$, and $s\in [0,1]$ one can define an inner product on $\mathcal {A}$ by

2.3 $$\begin{aligned} \langle {X,Y}\rangle _s = \tau [X^* \sigma ^{s} Y\sigma ^{1-s}]\ . \end{aligned}$$

Note that by cyclicity of the trace, $\langle {X,X}\rangle _s = \tau [|\sigma ^{s/2}X \sigma ^{(1-s)/2}|^2] \ge 0$, so that $\langle {\cdot , \cdot }\rangle _s$ is indeed a positive definite sesquilinear form. The inner products for $s=0$ and $s=\frac{1}{2}$ will come up frequently in what follows, and they have their own names: $\langle {\cdot ,\cdot }\rangle _0$ is the Gelfand–Naimark–Segal inner product, denoted $\langle {\cdot , \cdot }\rangle _{L^{2}_\mathrm{GNS}(\sigma )}$, and $\langle {\cdot , \cdot }\rangle _{1/2}$ is the Kubo–Martin–Schwinger inner product, denoted $\langle {\cdot , \cdot }\rangle _{L^2_\mathrm{KMS}(\sigma )}$. We shall write $\mathcal {A}= L^2_\mathrm{GNS}(\mathcal {A}, \sigma )$ (resp. $\mathcal {A}= L^2_\mathrm{KMS}(\mathcal {A}, \sigma )$) if we want to stress this Hilbert space structure.

Suppose, for some $s\in [0,1]$, that $\mathscr {P}_t$ is self-adjoint with respect to the $\langle \cdot ,\cdot \rangle _s$ inner product. Then, for all $A \in \mathcal {A}$,

$$\begin{aligned} \tau [(\mathscr {P}_t^\dagger \sigma ) A]= & {} \tau [\sigma \mathscr {P}_t A ] = \tau [\sigma ^{1-s} {\mathbf{1}}\sigma ^s \mathscr {P}_t A ] =\langle {\mathbf{1}}, \mathscr {P}_t A\rangle _s\\= & {} \langle \mathscr {P}_t{\mathbf{1}}, A\rangle _s = \langle {\mathbf{1}}, A\rangle _s = \tau [\sigma A ] \ . \end{aligned}$$

Hence for each of these inner products, self-adjointness of $\mathscr {P}_t$ implies that $\sigma$ is invariant under $\mathscr {P}_t^\dagger$.

The following lemma of Alicki [1] relates some of the possible definitions of detailed balance; a proof may be found in [10].

#### Lemma 2.1

Let $\mathscr {K}$ be a real linear transformation on $\mathcal {A}$. If $\mathscr {K}$ is self-adjoint with respect to the $\langle \cdot , \cdot \rangle _s$ inner product for some $s\in [0,1/2)\cup (1/2,1]$, then $\mathscr {K}$ commutes with $\Delta _\sigma$, and $\mathscr {K}$ is self-adjoint with respect to $\langle \cdot , \cdot \rangle _s$ for all $s\in [0,1]$, including $s=1/2$.

As we have remarked, for a QMS $(\mathscr {P}_t)_{t\ge 0}$, each $\mathscr {P}_t$ is real, and so $\mathscr {P}_t$ is self-adjoint with respect to the GNS inner product if and only if it is self-adjoint with respect to the $\langle \cdot , \cdot \rangle _s$ inner product for all $s\in [0,1]$. However, if each $\mathscr {P}_t$ is self-adjoint with respect to the KMS inner product, then it need not be self-adjoint with respect to the GNS inner product: There exist QMS for which each $\mathscr {P}_t$ is self-adjoint with respect to the KMS inner product, but for which $\mathscr {P}_t$ does not commute with $\Delta _\sigma$, and therefore cannot be self-adjoint with respect to the GNS inner product. A simple example is provided in appendix B of [10]. The generators of QMS such that $\mathscr {P}_t$ is self-adjoint with respect to the KMS inner product have been investigated by Fagnola and Umanita [20]. However, there is a third notion of detailed balance that is natural in the present context, namely the requirement that each $\mathscr {P}_t$ be self-adjoint with respect to the Boguliobov–Kubo–Mori inner product:

#### Definition 2.2

(*BKM inner product*) The *BKM inner product* is defined by

2.4 $$\begin{aligned} \langle {A, B}\rangle _{L^2_\mathrm{BKM}(\sigma )} = \int _0^1 \langle {A,B}\rangle _s \; \mathrm {d}s\ . \end{aligned}$$

By what we have remarked above, if each $\mathscr {P}_t$ is self-adjoint with respect to the GNS inner product, then each $\mathscr {P}_t$ is self-adjoint with respect to the BKM inner product. However, as will be discussed at the end of this section, the converse is not in general true. The relevance of the BKM version of detailed balance is due to the following result that we show in Theorem 2.9: If the forward Kolmogorov equation for an ergodic QMS $(\mathscr {P}_t)_{t\ge 0}$ with invariant state $\sigma \in {{\mathfrak {P}}}_+$ is gradient flow for the quantum relative entropy ${{\,\mathrm{Ent}\,}}_{\sigma }(\rho ) := \tau [\rho ( \log \rho - \log \sigma ) ]$ with respect to some continuously differentiable Riemannian metric on ${{\mathfrak {P}}}_+$, then each $\mathscr {P}_t$ is self-adjoint with respect to the BKM inner product. The BKM inner product is closely connected to the relative entropy functional, and for this reason it appears in some of the functional inequalities that we consider in Sect. 11.

On the other hand, only when each $\mathscr {P}_t$ is self-adjoint with respect to the GNS inner product do we have a construction of such a Riemannian metric. The same is true for other constructions of Riemannian metrics on ${{\mathfrak {P}}}_+$ for which QMS become gradient flow for ${{\,\mathrm{Ent}\,}}_{\sigma }(\rho )$, in particular see [36]. Since most of this paper is concerned with our construction and its consequences, we make the following definition:

#### Definition 2.3

(*Detailed balance*) Let $\sigma \in \mathcal {A}$ be non-negative. We say that a quantum Markov semigroup $(\mathscr {P}_t)_{t\ge 0}$ satisfies the *detailed balance condition* with respect to $\sigma$ if for each $t>0$, $\mathscr {P}_t$ is self-adjoint with respect to the GNS inner product on $\mathcal {A}$ induced by $\sigma$, i.e.,

$$\begin{aligned} \tau [\sigma A^* \mathscr {P}_t B ] = \tau [\sigma (\mathscr {P}_t A)^* B ] \quad \text { for all } A, B \in \mathcal {A}\ . \end{aligned}$$

We shall write that $(\mathscr {P}_t)_t$ satisfies $\sigma$-DBC for brevity.

The following result gives the general form of the generator of quantum Markov semigroups on $B(\mathscr {H})$ satisfying detailed balance. This result is due to Alicki [1, Theorem 3]; see [10] for a detailed proof.

#### Theorem 2.4

(Structure of Lindblad operators with detailed balance) Let $\mathscr {P}_t = e^{t \mathscr {L}}$ be a quantum Markov semigroup on $B(\mathscr {H})$ satisfying detailed balance with respect to $\sigma \in {{\mathfrak {P}}}_{+}$. Then the generator $\mathscr {L}$ and its adjoint $\mathscr {L}^\dagger$ have the form

2.5 $$\begin{aligned} \mathscr {L}&= \sum _{j \in \mathcal {J}} e^{-\omega _j/2} \mathscr {L}_j \ , \qquad \mathscr {L}_{j}(A) = V_j^* [A, V_j] + [V_j^* , A] V_j \ , \end{aligned}$$

2.6 $$\begin{aligned} \mathscr {L}^\dagger&= \sum _{j \in \mathcal {J}} e^{-\omega _j/2} \mathscr {L}_{j}^\dagger \ , \qquad \mathscr {L}^\dagger _{j}(\rho ) = [V_j, \rho V_j^*] + [V_j \rho , V_j^* ] \ , \end{aligned}$$

where $\mathcal {J}$ is a finite index set, the operators $V_j \in B(\mathscr {H})$ satisfy $\{ V_{j} \}_{j \in \mathcal {J}} = \{ V_{j}^{*} \}_{j \in \mathcal {J}}$, and $\omega _{j} \in {{\mathbb {R}}}$ satisfies

2.7 $$\begin{aligned} \Delta _\sigma V_j&= e^{-\omega _j} V_j \quad \text {for all } j \in \mathcal {J}\ . \end{aligned}$$

For $j \in \mathcal {J}$, let $j^{*} \in \mathcal {J}$ be an index such that $V_{j^{*}} = V_{j}^{*}$. It follows from (2.7) that

$$\begin{aligned} \omega _{j^{*}} = - \omega _{j} \ . \end{aligned}$$

Moreover, if we define $H = -\log \sigma$, (2.7) is equivalent to the commutator identity $[V_j,H] = - \omega _{j}V_j$. Furthermore, in our finite-dimensional context, the identity

2.8 $$\begin{aligned} \Delta _\sigma ^t V_j = e^{- \omega _j t} V_j \end{aligned}$$

is valid for some $t\ne 0$ in ${{\mathbb {R}}}$ if and only if it is valid for all $t \in {{\mathbb {C}}}$.

### Gradient Flow Structure for the Non-commutative Dirichlet Energy

Let $(\mathscr {P}_t)_{t\ge 0}$ be a quantum Markov semigroup satisfying detailed balance with respect to $\sigma \in {{\mathfrak {P}}}_+(\mathcal {A})$. Let $\mathscr {L}$ be the generator, so that for each $t>0$, $\mathscr {P}_t = e^{t\mathscr {L}}$. As explained in the discussion leading up to Definition 2.3, for each *t*, $\mathscr {P}_t$ is self-adjoint with respect to *both* the GNS and the KMS inner products induced by $\sigma$. Therefore, we may define a *Dirichlet form* $\mathscr {E}$ on $\mathcal {A}$ by

2.9 $$\begin{aligned} \mathscr {E}(A,A) = \lim _{t\downarrow 0}\frac{1}{t} \langle A, (I - \mathscr {P}_t )A\rangle \end{aligned}$$

where the inner product is either the GNS or the KMS inner product. Then, either way, the *Kolmogorov backward equation* $\partial _t A = \mathscr {L}A$ is a gradient flow equation for the energy $\mathscr {E}(A,A)$ with respect to the chosen $L^2$ metric.

The class of bilinear forms $\mathscr {E}$ defined in terms of a self-adjoint QMS $(\mathscr {P}_t)_{t\ge 0}$ through (2.9) is, by definition, the class of *conservative completely Dirichlet forms* on $\mathcal {A}$ in the specified inner product. The abstract Beurling–Deny Theorem, discussed in the next section, provides an intrinsic characterization of such bilinear forms.

Although Definition 2.3 might seem to suggest that the natural choice of the $L^2$ metric is the one given by the GNS inner product, we shall show that in some sense it is the KMS inner product that is more natural: The Dirichlet form defined by (2.9) using the KMS inner product induced by $\sigma$ can be expressed in terms of a “squared gradient”, and the associated non-commutative differential calculus will turn out to be very useful for investigating properties of the flow specified by $\partial _t A = \mathscr {L}A$. A somewhat different construction leading to the representation of Dirichlet forms with respect to the KMS metric in terms of derivations has been given by Cipriani and Sauvageot [13]. Our “derivatives” are not always derivations, and this more general structure is suited to applications. Indeed, one of the first non-commutative Dirichlet forms to be investigated in mathematical physics, the *Clifford Dirichlet form* of Gross, is most naturally expressed in terms of a sum of squares of *skew derivations*. The flexibility of our framework will be essential to our later applications. In this part of the introduction, we present only some of the key computations in a simple setting involving derivations to explain the roles of the KMS inner product. Our more general framework will be presented in Sect. 4.

Consider a Lindblad generator $\mathscr {L}$ given as in Theorem 2.4. To bring out the analogy with classical Kolmogorov backward diffusion equations of the form

2.10 $$\begin{aligned} \frac{\partial }{\partial t}f(x,t) = \Delta f(x,t) + (\nabla \log \sigma (x))\cdot \nabla f(x,t)\ , \end{aligned}$$

where $\sigma$ is a smooth, strictly positive probability density on ${{\mathbb {R}}}^n$, we define the following *partial derivative operators on* $\mathcal {A}$:

2.11 $$\begin{aligned} \partial _j A = [V_j, A] \ , \end{aligned}$$

$j\in \mathcal {J}$. Note that $\partial _{j}^\dagger = \partial _{j^{*}}$, where we recall that $j^{*}$ denotes an index such that $V_{j^*} = V_j^*$. An easy computation shows that the adjoint of $\partial _{j}$ with respect to $\langle {\cdot ,\cdot }\rangle _{L_\mathrm{KMS}^{2}(\sigma )}$ is given by

2.12 $$\begin{aligned} \partial _{j,\sigma }^\dagger A = \sigma ^{-1/2} \partial _{j}^\dagger \big ( \sigma ^{1/2} A \sigma ^{1/2} \big ) \sigma ^{-1/2} \ . \end{aligned}$$

#### Proposition 2.5

(Divergence form representation of $\mathscr {L}$) For all $A \in \mathcal {A}$ we have

$$\begin{aligned} \mathscr {L}A = - \sum _{j \in \mathcal {J}} \partial _{j,\sigma }^\dagger \partial _{j} A \ . \end{aligned}$$

#### Proof

Using (2.12) and (2.8) we obtain

$$\begin{aligned} \sum _{j \in \mathcal {J}} \partial _{j,\sigma }^\dagger \partial _{j} A&= \sum _{j \in \mathcal {J}} \partial _{j,\sigma }^\dagger (V_j A - A V_j )\\&= \sum _{j \in \mathcal {J}} \sigma ^{-1/2} \partial _{j}^\dagger \big ( \sigma ^{1/2} (V_j A - A V_j ) \sigma ^{1/2} \big ) \sigma ^{-1/2} \\&= \sum _{j \in \mathcal {J}} \sigma ^{-1/2} \Big ( V_j^* \sigma ^{1/2} (V_j A - A V_j ) \sigma ^{1/2} - \sigma ^{1/2} (V_j A - A V_j ) \sigma ^{1/2} V_j^* \Big ) \sigma ^{-1/2} \\&= \sum _{j \in \mathcal {J}} \Big ( e^{-\omega _j/2} V_j^* (V_j A - A V_j ) - e^{\omega _j/2} (V_j A - A V_j ) V_j^* \Big ) \\&= \sum _{j \in \mathcal {J}} \Big ( e^{-\omega _j/2} V_j^* (V_j A - A V_j ) - e^{-\omega _j/2} (V_j^* A - A V_j^* ) V_j \Big )\\&= -\sum _{j \in \mathcal {J}} e^{-\omega _j/2} \mathscr {L}_j(A) = -\mathscr {L}A\ , \end{aligned}$$

as desired. $$\square $$

Proposition 2.5 can be stated equivalently as an integration by parts identity

2.13 $$\begin{aligned} \sum _{j \in \mathcal {J}} \langle {\partial _j A, \partial _j B}\rangle _{L_\mathrm{KMS}^{2}(\sigma )} = - \langle {A,\mathscr {L}B}\rangle _{L_\mathrm{KMS}^{2}(\sigma )} \quad \text { for } A, B \in \mathcal {A}\ . \end{aligned}$$

It is now immediate that the backward equation $\partial _t A = \mathscr {L}A$ with $\mathscr {L}$ given by (2.1), is the gradient flow equation for the energy $\mathscr {E}(A,A)$ with respect to the KMS inner product induced by $\sigma$. What makes this particular gradient flow representation especially useful is that the Dirichlet form $\mathscr {E}$ is written, in (2.13), as the expectation of a squared gradient. That is, the gradient flow structure given here is analogous to the gradient flow formulation for the Kolmogorov backward equation (2.10) for the Dirichlet energy $\mathcal {D}_{class}(f) = \frac{1}{2}\int _{{{\mathbb {R}}}^n} |\nabla f(x)|^2 \sigma (x)\; \mathrm {d}x$. This would not be the case if we had considered the Dirichlet form based on the GNS inner product: We would have a gradient flow structure, but the Dirichlet form would not be the expectation of a squared gradient in any meaningful sense; see however, Proposition 4.12 below for a related representation.

In the next section we show how the non-commutative differential calculus associated to the Dirichlet from $\mathscr {E}$ allows us to write the corresponding *forward equation* as gradient flow for the relative entropy with respect to a Riemannian metric constructed in terms of this differential calculus.

### A Gradient Flow Structure for the Quantum Relative Entropy

Consider the * quantum relative entropy functionals* ${{\,\mathrm{Ent}\,}}_{\sigma }: {{\mathfrak {P}}}_{+} \rightarrow {{\mathbb {R}}}$ given by

$$\begin{aligned} {{\,\mathrm{Ent}\,}}_{\sigma }(\rho ) := \tau [\rho ( \log \rho - \log \sigma ) ] \ . \end{aligned}$$

Our goal is to sketch a proof of one of the results of [10, 36], namely that the quantum master equation $\partial _t \rho = \mathscr {L}^\dagger \rho$, which is a Kolmogorov *forward equation*, can be formulated as the gradient flow equation for ${{\,\mathrm{Ent}\,}}_{\sigma }$ with respect to a suitable Riemannian metric on ${{\mathfrak {P}}}_{+}$. The construction of the Riemannian metric will make use of the “quantum directional derivatives” $\partial _j$ introduced in the last subsection.

Since ${{\mathfrak {P}}}_{+}$ is a relatively open subset of the ${{\mathbb {R}}}$-affine subspace $\{ A \in \mathcal {A}_{h} : \tau [A] = 1 \}$, we may identify, at each point in $\rho \in {{\mathfrak {P}}}_{+}$, its tangent space $T_{\rho } {{\mathfrak {P}}}_{+}$ with $\mathcal {A}_{0} := \{ A \in \mathcal {A}_{h} : \tau [A] = 0 \}$. The cotangent space $T_{\rho }^\dagger {{\mathfrak {P}}}_{+}$ may also be identified with $\mathcal {A}_{0}$ through the duality pairing $\langle {A,B}\rangle = \tau [A B]$ for $A, B \in \mathcal {A}_{0}$.

Let $(g_{\rho })_{\rho \in {{\mathfrak {P}}}_{+}}$ be a Riemannian metric on ${{\mathfrak {P}}}_{+}$, i.e., a collection of positive definite bilinear forms $g_{\rho } : T_{\rho }{{\mathfrak {P}}}_{+} \times T_{\rho }{{\mathfrak {P}}}_{+} \rightarrow {{\mathbb {R}}}$ depending smoothly on $\rho \in {{\mathfrak {P}}}_{+}$. Consider the associated operator $\mathscr {G}_{\rho } : T_{\rho }{{\mathfrak {P}}}_{+} \rightarrow T_{\rho }^{\dagger }{{\mathfrak {P}}}_{+}$ defined by $\langle {A, \mathscr {G}_{\rho } B}\rangle = g_{\rho }(A, B)$ for $A, B \in T_{\rho } {{\mathfrak {P}}}_{+}$. Clearly, $\mathscr {G}_{\rho }$ is invertible and self-adjoint with respect to the Hilbert–Schmidt inner product on $\mathcal {A}_{0}$. Define $\mathscr {K}_{\rho } : T_{\rho }^{\dagger }{{\mathfrak {P}}}_{+} \rightarrow T_{\rho } {{\mathfrak {P}}}_{+}$ by $\mathscr {K}_{\rho } = (\mathscr {G}_{\rho })^{-1}$, so that

2.14 $$\begin{aligned} g_{\rho }(A,B) = \langle { A, \mathscr {K}_{\rho }^{-1} B}\rangle \ . \end{aligned}$$

In many situations of interest it is convenient to define the metric $g_{\rho }$ by specifying the operator $\mathscr {K}_{\rho }$. In such cases, there is often no explicit formula available for $\mathscr {G}_{\rho }$ and $g_{\rho }$.

For a smooth functional $\mathcal {F}: {{\mathfrak {P}}}_{+} \rightarrow {{\mathbb {R}}}$ and $\rho \in {{\mathfrak {P}}}_{+}$, its *differential* $\mathrm {D}\mathcal {F}(\rho ) \in T_{\rho }^{\dagger }{{\mathfrak {P}}}_{+}$ is defined by $\lim _{\varepsilon \rightarrow 0} \varepsilon ^{-1}(\mathcal {F}(\rho + \varepsilon A) - \mathcal {F}(\rho ) ) = \langle {A,\mathrm {D}\mathcal {F}(\rho )}\rangle$ for $A \in T_{\rho }{{\mathfrak {P}}}_{+}$ (independently of the Riemannian metric $g_{\rho }$). Its *gradient* $\nabla _g \mathcal {F}(\rho ) \in T_{\rho }{{\mathfrak {P}}}_{+}$ depends on the Riemannian metric through the duality formula $g_{\rho }(A, \nabla _g \mathcal {F}(\rho )) = \langle {A, \mathrm {D}\mathcal {F}(\rho )}\rangle$ for $A \in T_{\rho }{{\mathfrak {P}}}_{+}$. It follows that $\mathscr {G}_{\rho }\nabla _g \mathcal {F}(\rho ) = \mathrm {D}\mathcal {F}(\rho )$, or equivalently

$$\begin{aligned} \nabla _g \mathcal {F}(\rho ) = \mathscr {K}_{\rho } \mathrm {D}\mathcal {F}(\rho ) \ . \end{aligned}$$

The gradient flow equation $\partial _{t}\rho = - \nabla _g \mathcal {F}(\rho )$ takes the form

$$\begin{aligned} \partial _{t}\rho = - \mathscr {K}_{\rho } \mathrm {D}\mathcal {F}(\rho ) \ . \end{aligned}$$

Let us now focus on the relative entropy functional ${{\,\mathrm{Ent}\,}}_\sigma$ for some $\sigma \in {{\mathfrak {P}}}_+$, and note that its differential is given by

2.15 $$\begin{aligned} \mathrm {D}{{\,\mathrm{Ent}\,}}_{\sigma }(\rho ) = \log \rho - \log \sigma \ . \end{aligned}$$

Consider a generator $\mathscr {L}^\dagger$ written in the form (2.6), i.e.,

$$\begin{aligned} \mathscr {L}^\dagger&= \sum _{j \in \mathcal {J}} e^{-\omega _j/2} \mathscr {L}_{j}^\dagger \ , \qquad \mathscr {L}^\dagger _{j}(\rho ) = [V_j, \rho V_j^*] + [V_j \rho , V_j^* ] \ , \end{aligned}$$

where $\{V_j\}_{j\in \mathcal {J}}$ is a finite set of eigenvectors of $\Delta _\sigma$ such that $\{V_j^*\}_{j\in \mathcal {J}} = \{V_j\}_{j\in \mathcal {J}}$, and where $\Delta _\sigma V_j = e^{-\omega _j}V_j$ for some $\omega _j \in {{\mathbb {R}}}$. As before, we use the notation $\partial _j A := [V_j, A]$.

For $\rho \in {{\mathfrak {P}}}$ we define ${\widehat{\rho }}_j\in \mathcal {A}\otimes \mathcal {A}$ by

$$\begin{aligned} \widehat{\rho _{j}}= \int _0^1 \big (e^{\omega _j /2} \rho \big )^{1-s} \otimes \big (e^{-\omega _j /2} \rho \big )^{s}\; \mathrm {d}s \ . \end{aligned}$$

We shall frequently make use of the *contraction operator* $\# : (\mathcal {A}\otimes \mathcal {A}) \times \mathcal {A}\rightarrow \mathcal {A}$ defined by

2.16 $$\begin{aligned} (A \otimes B) \# C&:= ACB \end{aligned}$$

and linear extension. A crucial step towards obtaining the gradient flow structure is the following chain rule for the commutators $\partial _j$, which involves the differential of the entropy.

#### Lemma 2.6

(Chain rule for the logarithm) For all $\rho \in {{\mathfrak {P}}}_+$ and $j \in \mathcal {J}$ we have

2.17 $$\begin{aligned} e^{-\omega _j/2}V_j \rho - e^{\omega _j/2}\rho V_j&= \widehat{\rho _{j}}\# \partial _{j}(\log \rho - \log \sigma ) \ . \end{aligned}$$

#### Proof

Using (2.7) we infer that

$$\begin{aligned} \partial _{j}( \log \rho - \log \sigma ) = V_j \log (e^{-\omega _j/2} \rho ) - \log (e^{\omega _j/2} \rho ) V_j \ . \end{aligned}$$

Consider the spectral decomposition $\rho = \sum _\ell \lambda _\ell E_\ell$, where $\lambda _\ell > 0$ for all *i*, and $\{E_\ell \}_\ell$ are the spectral projections, so that $E_\ell E_m = \delta _{\ell m} E_\ell$ and $\sum _\ell E_\ell = {\mathbf{1}}$. Observe that

$$\begin{aligned} {\widehat{\rho }}_{j} = \sum _{\ell ,m} \Lambda (e^{\omega _j/2} \lambda _\ell , e^{-\omega _j/2} \lambda _m) E_\ell \otimes E_m \ , \end{aligned}$$

where $\Lambda (\xi ,\eta ) = \int _0^1 \xi ^{1-s} \eta ^s \; \mathrm {d}s = \frac{\xi - \eta }{\log \xi - \log \eta }$ denotes the logarithmic mean of $\xi$ and $\eta$. Thus,

$$\begin{aligned}&\widehat{\rho _{j}}\# \big ( \partial _{j}( \log \rho - \log \sigma ) \big ) \\&\quad = \sum _{\ell , m, p} \Lambda (e^{\omega _j/2} \lambda _\ell , e^{-\omega _j/2} \lambda _m) E_\ell \Big ( \log (e^{-\omega _j/2} \lambda _p) V_j E_p - \log (e^{\omega _j/2} \lambda _p) E_p V_j \Big ) E_m\\&\quad = \sum _{\ell , m} \Lambda (e^{\omega _j/2} \lambda _\ell , e^{-\omega _j/2} \lambda _m) \Big ( \log (e^{-\omega _j/2} \lambda _m) - \log (e^{\omega _j/2} \lambda _\ell ) \Big ) E_\ell V_j E_m\\&\quad = \sum _{\ell , m} \big ( e^{-\omega _j/2} \lambda _m - e^{\omega _j/2} \lambda _\ell \big ) E_\ell V_j E_m\\&\quad = e^{-\omega _j/2} V_j \rho - e^{\omega _j/2} \rho V_j\ , \end{aligned}$$

which proves (2.17). $$\square $$

For $\rho \in {{\mathfrak {P}}}_+$ we define the operator $\mathscr {K}_\rho : \mathcal {A}\rightarrow \mathcal {A}$ by

2.18 $$\begin{aligned} \mathscr {K}_\rho A := \sum _{j \in \mathcal {J}} \partial _{j}^\dagger \big ( \widehat{\rho _{j}}\# \partial _{j} A \big ) \ . \end{aligned}$$

Since ${{\,\mathrm{Tr}\,}}(A^* \mathscr {K}_\rho B) = \overline{{{\,\mathrm{Tr}\,}}(B^* \mathscr {K}_\rho A)}$ for $A, B \in \mathcal {A}$, it follows that $\mathscr {K}_\rho$ is a non-negative self-adjoint operator on $L^2(\mathcal {A}, \tau )$ for each $\rho \in {{\mathfrak {P}}}_+$. Assuming that $\mathscr {P}_t$ is ergodic, the operator $\mathscr {K}_\rho : \mathcal {A}_0 \rightarrow \mathcal {A}_0$ is invertible for each $\rho \in {{\mathfrak {P}}}_+$ (see Corollary 7.4 below for a proof of this statement). Since $\mathscr {K}_\rho$ depends smoothly on $\rho$, it follows that $\mathscr {K}_\rho$ induces a Riemannian metric on ${{\mathfrak {P}}}_+$ defined by (2.14).

The following result shows that the Kolmogorov forward equation $\partial _t \rho = \mathscr {L}^\dagger \rho$ can be formulated as the gradient flow equation for ${{\,\mathrm{Ent}\,}}_{\sigma }$.

#### Proposition 2.7

For $\rho \in {{\mathfrak {P}}}_+$ we have the identity

$$\begin{aligned} \mathscr {L}^\dagger \rho = - \mathscr {K}_\rho \mathrm {D}{{\,\mathrm{Ent}\,}}_{\sigma }(\rho )\ , \end{aligned}$$

hence the gradient flow equation of ${{\,\mathrm{Ent}\,}}_\sigma$ with respect to the Riemannian metric induced by $(\mathscr {K}_\rho )_\rho$ is the master equation $\partial _t \rho = \mathscr {L}^\dagger \rho$.

#### Proof

Using the identity (2.15), the chain rule from Lemma 2.6, and the fact that $\{V_j\} = \{V_j^*\}$ and $\omega _{j^*} = -\omega _j$, we obtain

$$\begin{aligned} \mathscr {K}_\rho \mathrm {D}{{\,\mathrm{Ent}\,}}_{\sigma }(\rho )&= \sum _{j \in \mathcal {J}} \partial _{j}^\dagger \big ( \widehat{\rho _{j}}\# \partial _{j} (\log \rho - \log \sigma ) \big )\\&= \sum _{j \in \mathcal {J}} \partial _{j}^\dagger \big ( e^{-\omega _j/2}V_j \rho - e^{\omega _j/2}\rho V_j \big )\\&= \frac{1}{2} \sum _{j \in \mathcal {J}} \Big ( \partial _{j}^\dagger \big ( e^{-\omega _j/2}V_j \rho - e^{\omega _j/2}\rho V_j \big ) + \partial _{j} \big ( e^{\omega _{j}/2}V_j^* \rho - e^{-\omega _j/2}\rho V_j^* \big ) \Big ) \\&= - \frac{1}{2}\sum _{j \in \mathcal {J}} e^{-\omega _j/2} \Big ( [V_j, \rho V_j^*] + [V_j \rho , V_j^* ] \Big ) + e^{\omega _j/2} \Big ( [V_j^*, \rho V_j] + [V_j^* \rho , V_j ]\Big ) \\&= - \sum _{j \in \mathcal {J}} e^{-\omega _j/2} \Big ( [V_j, \rho V_j^*] + [V_j \rho , V_j^* ] \Big ) \\&= - \mathscr {L}^\dagger \rho \ , \end{aligned}$$

which is the desired identity. $\square$

In this paper we extend this result into various directions: we consider more general entropy functionals, more general Riemannian metrics, and nonlinear evolution equations.

#### Remark 2.8

The gradient flow structure given in Proposition 2.7 can be viewed as a non-commutative analogue of the Kantorovich gradient flow structure obtained by Jordan, Kinderlehrer and Otto [29] for the *Kolmogorov backward equation*

$$\begin{aligned} \frac{\partial }{\partial t}\rho (x,t) = \Delta \rho (x,t) - \nabla \cdot ( \rho (x,t) \nabla \log \sigma (x) ) \ . \end{aligned}$$

This structure is formally given in terms of the operator $K_\rho$ defined by

$$\begin{aligned} K_{\rho } \psi = - \nabla \cdot (\rho \nabla \psi ) \ , \end{aligned}$$

for probability densities $\rho$ on ${{\mathbb {R}}}^n$ and suitable functions $\psi : {{\mathbb {R}}}^n \rightarrow {{\mathbb {R}}}$ in analogy with (2.18). As the differential of the relative entropy ${{\,\mathrm{Ent}\,}}_\sigma (\rho ) = \int _{{{\mathbb {R}}}^n} \rho (x)\log \frac{\rho (x)}{\sigma (x)} \; \mathrm {d}x$ is given by $\mathrm {D}{{\,\mathrm{Ent}\,}}_\sigma (\rho ) = 1 + \log \frac{\rho }{\sigma }$, we have

$$\begin{aligned} K_\rho \mathrm {D}{{\,\mathrm{Ent}\,}}_\sigma (\rho ) = - \Delta \rho + \nabla \cdot (\rho \nabla \log \sigma )\ , \end{aligned}$$

which is the commutative counterpart of Proposition 2.7.

### The Necessity of BKM-Detailed Balance

In the classical setting of irreducible finite Markov chain, Dietert [15] has proven that if the Kolmogorov forward equation for a Markov semigroup can be written as gradient flow for the relative entropy with respect to the unique invariant measure for some continuously differentiable Riemannian metric, then the Markov chain is necessarily reversible. That is, it satisfies the classical detailed balance condition.

#### Theorem 2.9

Let $(\mathscr {P}_t)_{t\ge 0}$ be an ergodic QMS with generator $\mathscr {L}$ and invariant state $\sigma \in {{\mathfrak {P}}}_+$. If there exists a continuously differentiable Riemannian metric $(g_\rho )$ on ${{\mathfrak {P}}}_+$ such that the quantum master equation $\partial \rho = \mathscr {L}^\dagger \rho$ is the gradient flow equation for ${{\,\mathrm{Ent}\,}}_\sigma$ with respect to $(g_\rho )$, then each $\mathscr {P}_t$ is self-adjoint with respect to the BKM inner product associated to $\sigma$.

Before beginning the proof, we recall some relevant facts, and introduce some notation. Regarding $\sigma$ as an element of ${{\mathbb {M}}}_n({{\mathbb {C}}})$, we define the operator $\mathscr {M}$ on ${{\mathbb {M}}}_n({{\mathbb {C}}})$ by

$$\begin{aligned} \mathscr {M}A = \int _0^1 \sigma ^{1-s}A \sigma ^s \; \mathrm {d}s \ . \end{aligned}$$

A simple calculation shows that ${\mathscr {M}}$ is the derivative of the matrix exponential function. Its inverse is the derivative of the matrix logarithm function:

$$\begin{aligned} \mathscr {M}^{-1} A = \int _0^\infty \frac{1}{t+\sigma } A \frac{1}{t+\sigma } \; \mathrm {d}t \ , \end{aligned}$$

(see Example 6.5 below for more details). While the matrix logarithm function is monotone, the matrix exponential is not. Thus $\mathscr {M}^{-1}$ preserves positivity, but $\mathscr {M}$ does not. In fact $A \mapsto \mathscr {M}^{-1} A$ is evidently completely positive. The BKM inner product can now be written as

$$\begin{aligned} \langle {A,B}\rangle _{L^2_\mathrm{BKM}(\sigma )} = \tau [A^* \mathscr {M}B ] = \tau [\mathscr {M}(A^*) B ] \ . \end{aligned}$$

#### Proof of Theorem 2.9

As before, it will be convenient to consider the operators $(\mathscr {K}_\rho )$ defined by (2.14). Since $\mathrm {D}{{\,\mathrm{Ent}\,}}_{\sigma }(\rho ) = \log \rho - \log \sigma$, the gradient flow equation $\partial _t \rho = - \mathscr {K}_{\rho } \mathrm {D}{{\,\mathrm{Ent}\,}}_{\sigma }(\rho )$ becomes

2.19 $$\begin{aligned} \mathscr {L}^\dagger \rho = - \mathscr {K}_\rho (\log \rho - \log \sigma ) \ . \end{aligned}$$

Applying this identity to $\rho _\varepsilon = \sigma + \varepsilon A$ for $A \in \mathcal {A}_0$, and differentiating at $\varepsilon = 0$, we obtain using the identity $\partial _\varepsilon |_{\varepsilon = 0} \log \rho _\varepsilon = \mathscr {M}^{-1} A$ that

2.20 $$\begin{aligned} \mathscr {L}^\dagger A = - \mathscr {K}_\sigma \mathscr {M}^{-1} A \ , \end{aligned}$$

Consequently, for $$A, B \in \mathcal {A}$$,

$$\begin{aligned} \langle {\mathscr {L}A, B}\rangle _{L^2_\mathrm{BKM}(\sigma )} = \tau [ (\mathscr {L}A)^* \mathscr {M}B] = \tau [ A^* \mathscr {L}^\dagger \mathscr {M}B] = - \tau [ A^* \mathscr {K}_\sigma B] \ . \end{aligned}$$

As $g_\sigma$ is a symmetric bilinear form, the operator $\mathscr {K}_\sigma$ is self-adjoint with respect to the Hilbert-Schmidt scalar product. This implies the result. $\square$

We are unaware of any investigation of the nature of the class of QMS generators that are self-adjoint for the BKM inner product associated to their invariant state $\sigma$. Therefore we briefly demonstrate that this class *strictly* includes the class of QMS generators that are self-adjoint for the GNS inner product associated to their invariant state $\sigma$.

Let $\mathscr {P}$ be a unital completely positive map such that $\mathscr {P}^\dagger \sigma = \sigma$, and define

$$\begin{aligned} {{\widetilde{\mathscr {P}}}}(A) = {\mathscr {M}}^{-1}(\sigma ^{1/2} \mathscr {P}(A) \sigma ^{1/2})\ . \end{aligned}$$

Note that

$$\begin{aligned} {\mathscr {M}}^{-1}(\sigma ^{1/2} A \sigma ^{1/2}) = \int _0^\infty \frac{\sigma ^{1/2}}{t + \sigma } A \frac{\sigma ^{1/2}}{t + \sigma } \; \mathrm {d}t \end{aligned}$$

defines a completely positive and unital operator, and hence ${\widetilde{\mathscr {P}}}$ is completely positive and unital. Moreover,

$$\begin{aligned} {\widetilde{\mathscr {P}}}^\dagger (A) = \mathscr {P}^\dagger ( {\mathscr {M}}^{-1}(\sigma ^{1/2} A \sigma ^{1/2}))\ , \end{aligned}$$

and hence ${\widetilde{\mathscr {P}}}^\dagger \sigma = \sigma$. Now observe that ${\widetilde{\mathscr {P}}}$ is self-adjoint with respect to the BKM inner product if and only if $\mathscr {P}$ is self-adjoint for the KMS inner product. In fact, for all $A, B \in \mathcal {A}$,

$$\begin{aligned} \langle {{\widetilde{\mathscr {P}}} A , B}\rangle _{L^2_\mathrm{BKM}(\sigma )} = \tau [ \mathscr {M}( \mathscr {M}^{-1} (\sigma ^{1/2} \mathscr {P}(A^*) \sigma ^{1/2})) B] = \langle {\mathscr {P}A, B}\rangle _{L^2_\mathrm{KMS}(\sigma )}\ . \end{aligned}$$

Next, it is clear that ${\widetilde{\mathscr {P}}}$ commutes with $\Delta _\sigma$ if and only if $\mathscr {P}$ commutes with $\Delta _\sigma$. Since there exist completely positive unital maps $\mathscr {P}$ satisfying $\mathscr {P}^\dagger \sigma = \sigma$ that are KMS symmetric but do not commute with $\Delta _\sigma$, there exists completely positive unital maps ${\widetilde{\mathscr {P}}}$ satisfying ${\widetilde{\mathscr {P}}}^\dagger \sigma = \sigma$ that are BKM symmetric but do not commute with $\Delta _\sigma$.

Moreover, the class of completely positive unital maps ${\widetilde{\mathscr {P}}}$ satisfying ${\widetilde{\mathscr {P}}}^\dagger \sigma = \sigma$ that are BKM symmetric is in some sense larger than the class of completely positive unital maps $\mathscr {P}$ satisfying $\mathscr {P}^\dagger \sigma = \sigma$ that are KMS symmetric: The map $\mathscr {P}\mapsto {\widetilde{\mathscr {P}}}$ is invertible, but ${\mathscr {M}}$ is not even positivity preserving, let alone completely positive, so that

$$\begin{aligned} \mathscr {P}(A) = \sigma ^{-1/2} {\mathscr {M}}({\widetilde{\mathscr {P}}}(A) )\sigma ^{-1/2} \end{aligned}$$

need not be completely positive. It is therefore an interesting problem to characterize the QMS generators that are self-adjoint with respect to the BKM inner product.

## Beurling–Deny Theory in Finite-Dimensional von Neumann Algebras

In this section we recall some key results of Beurling–Deny theory that will be used in our construction of Dirichlet forms in Sect. 4. We present some proofs of known results for the reader’s convenience, especially when available references suppose a familiarity with the Tomita–Takesaki theory. However, Theorem 3.8, which singles out the KMS inner product, is new.

### Abstract Beurling–Deny Theory

In this subsection, $\mathcal {H}$ always denotes a *real* Hilbert space with inner product $\langle \cdot ,\cdot \rangle$. Let $\mathscr {P}$ be a cone in $\mathcal {H}$. That is, $\mathscr {P}$ is a convex subset of $\mathcal {H}$ such that if $\varphi \in \mathscr {P}$, then $\lambda \varphi \in \mathscr {P}$ for all $\lambda >0$. The cone $\mathscr {P}$ is *pointed* in case $\varphi \in \mathscr {P}$ and $-\varphi \in \mathscr {P}$ together imply that $\varphi =0$. In particular, a subspace of $\mathcal {H}$ is a cone, but it is not a pointed cone.

#### Definition 3.1

(*Dual cone*) The *dual cone* $\mathscr {P}^{\circ }$ of a cone $\mathscr {P}$ is the set

3.1 $$\begin{aligned} \mathscr {P}^\circ := \{ \psi \in \mathcal {H}\ :\ \langle \psi ,\varphi \rangle \ge 0\quad \mathrm{for\ all}\ \varphi \in \mathscr {P}\ \}\ . \end{aligned}$$

A cone $\mathscr {P}$ is *self-dual* in case $\mathscr {P}^\circ = \mathscr {P}$.

Let $\mathscr {P}$ be a non-empty self-dual cone in $\mathcal {H}$, and take $\varphi \in \mathcal {H}$. Since $\mathscr {P}$ is a non-empty closed, convex set, the Projection Lemma ensures the existence of $\mathsf {P}_{\mathscr {P}}(\varphi )\in \mathscr {P}$ such that

3.2 $$\begin{aligned} \Vert \varphi - \mathsf {P}_{\mathscr {P}}(\varphi )\Vert < \Vert \varphi - \psi \Vert \quad \mathrm{for\ all}\ \psi \in \mathscr {P},\ \psi \ne \mathsf {P}_{\mathscr {P}}(\varphi )\ . \end{aligned}$$

#### Theorem 3.2

(Decomposition Theorem) Let $\mathscr {P}$ be a non-empty self-dual cone in $\mathcal {H}$. Then for each $\varphi \in \mathcal {H}$, there exists a unique pair $\varphi _+,\varphi _-\in \mathscr {P}$ such that

3.3 $$\begin{aligned} \varphi = \varphi _+ - \varphi _-\quad \mathrm{and}\quad \langle \varphi _+,\varphi _-\rangle = 0 \ . \end{aligned}$$

In fact, $\varphi _+ = \mathsf {P}_{\mathscr {P}}(\varphi )$ and $\varphi _- = \mathsf {P}_{\mathscr {P}}(-\varphi )$, where $\mathsf {P}_\mathscr {P}$ denotes projection onto (the closed convex set) $\mathscr {P}$.

#### Proof

Define $\varphi _+ := \mathsf {P}_{\mathscr {P}}(\varphi )$. Then define $-\varphi _- := \varphi - \varphi _+$. We claim that $\varphi _-\in \mathscr {P}$. Indeed, for any $\psi \in \mathscr {P}$ and any $\epsilon >0$, $\varphi _+ + \epsilon \psi \in \mathscr {P}$, and hence,

$$\begin{aligned} \Vert \varphi _-\Vert ^2 = \Vert \varphi - \varphi _+\Vert ^2 < \Vert \varphi - (\varphi _+ +\epsilon \psi )\Vert ^2 = \Vert \varphi _-\Vert ^2 + 2\epsilon \langle \varphi _-,\psi \rangle + \epsilon ^2\Vert \psi \Vert ^2\ . \end{aligned}$$

Therefore, $\langle \varphi _-,\psi \rangle \ge 0$ for all $\psi \in \mathscr {P}$. Since $\mathscr {P}$ is self-dual, the claim follows.

To see that $\varphi _+$ and $\varphi _-$ are orthogonal, let $\epsilon \in (-1,1)$, so that $(1+\epsilon )\varphi _+ \in \mathscr {P}$. It follows that $\Vert \varphi _-\Vert ^2 = \Vert \varphi - \varphi _+\Vert ^2 \le \Vert \varphi - (1+\epsilon )\varphi _+\Vert ^2 = \Vert \varphi _-\Vert ^2 + 2\epsilon \langle \varphi _-,\varphi _+\rangle + \epsilon ^2 \Vert \varphi _+\Vert ^2$ which yields a contradiction for negative $\epsilon$ sufficiently close to zero, unless $\langle \varphi _-,\varphi _+\rangle = 0$. This proves existence of the decomposition. Now the fact that $\varphi _- = \mathsf {P}_{\mathscr {P}}(-\varphi )$ follows from a theorem of Moreau [37], as does the uniqueness of the decomposition, though both points can be proved directly by variations on the arguments just provided. $\square$

#### Definition 3.3

Let $\mathcal {H}$ be a real Hilbert space with a non-empty self-dual cone $\mathscr {P}$. For $\varphi$ in $\mathcal {H}$, define $\varphi _+$ and $\varphi _-$ as in Theorem 3.2. Then $\varphi _+$ is the *positive part of* $\varphi$, $\varphi _-$ is the *negative part of* $\varphi$, and $|\varphi |:= \varphi _+ + \varphi _-$ is the *absolute value of* $\varphi$. If $\varphi _- = 0$, we write $\varphi \ge 0$.

We next recall some elements of the abstract theory of symmetric Dirichlet forms. A *bilinear form* on a real Hilbert space $\mathcal {H}$ is a bilinear mapping $\mathscr {E}: \mathcal {D}\times \mathcal {D}\rightarrow {{\mathbb {R}}}$ where $\mathcal {D}\subseteq \mathcal {H}$ is a linear subspace (called the *domain* of $\mathscr {E}$). We say that $\mathscr {E}$ is *non-negative* if $\mathscr {E}(\varphi ,\varphi )\ge 0$ for all $\varphi \in \mathcal {D}$; *symmetric* if $\mathscr {E}(\varphi ,\psi ) = \mathscr {E}(\psi ,\varphi )$ for all $\psi , \psi \in \mathcal {D}$; *closed* if $\mathcal {D}$ is complete when endowed with the norm $\Vert \varphi \Vert _\mathscr {E}= (\Vert \varphi \Vert ^2 + \mathscr {E}(\varphi ,\varphi ))^{1/2}$; and *densely defined* if $\mathcal {D}$ is dense in $\mathcal {H}$.

#### Definition 3.4

(*Dirichlet form*) Let $\mathcal {H}$ be a real Hilbert space with a non-empty self-dual cone $\mathscr {P}$. A non-negative, symmetric, closed bilinear form $\mathscr {E}$ on $\mathcal {H}$ with dense domain $\mathcal {D}$ is a *Dirichlet form* in case $|\varphi | \in {{\mathcal {D}}}$ for all $\varphi \in {{\mathcal {D}}}$ , and

3.4 $$\begin{aligned} \mathscr {E}(|\varphi |,|\varphi |) \le \mathscr {E}(\varphi ,\varphi ) \ , \end{aligned}$$

or equivalently, if for all $\varphi \in \mathcal {D}$,

3.5 $$\begin{aligned} \mathscr {E}(\varphi _+,\varphi _-) \le 0 \ . \end{aligned}$$

To see the equivalence of (3.4) and (3.5), note that

$$\begin{aligned} \mathscr {E}(|\varphi |,|\varphi |) - \mathscr {E}(\varphi ,\varphi ) =4\mathscr {E}(\varphi _+,\varphi _-)\ . \end{aligned}$$

Given a non-negative, symmetric, closed bilinear form $\mathscr {E}$, the *operator* $\mathscr {L}: \mathcal {D}_\mathscr {L}\subseteq \mathcal {H}\rightarrow \mathcal {H}$ *associated to* $\mathscr {E}$ is defined by

$$\begin{aligned} \mathcal {D}_{\mathscr {L}}&:= \{ \psi \in \mathcal {D}\ | \ \exists \xi \in \mathcal {H}: \mathscr {E}(\varphi ,\psi ) = -\langle {\varphi , \xi }\rangle \ \ \forall \varphi \in \mathcal {D}\} \ , \qquad \mathscr {L}\psi := \xi \ . \end{aligned}$$

This operator is well-defined since $\mathcal {D}_\mathscr {L}$ is dense. Moreover, $\mathscr {L}$ is non-positive and self-adjoint.

The following abstract result by Ouhabaz [40] characterizes the invariance of closed convex sets under the associated semigroup (in a more general setting that includes nonsymmetric Dirichlet forms).

#### Theorem 3.5

(Ouhabaz’ Theorem) Let $\mathcal {H}$ be a real Hilbert space, and let $\mathscr {E}$ be a non-negative, symmetric, closed bilinear form with domain $\mathcal {D}$ and associated operator $\mathscr {L}$. Let $\mathcal {C}\subseteq \mathcal {H}$ be closed and convex. Then, the following assertions are equivalent:

1. $e^{t\mathscr {L}} \varphi \in \mathcal {C}$ for all $\varphi \in \mathcal {C}$ and all $t \ge 0$;
2. $\mathsf {P}_\mathcal {C}\varphi \in \mathcal {D}$ and $\mathscr {E}(\mathsf {P}_\mathcal {C}\varphi , \varphi - \mathsf {P}_\mathcal {C}\varphi ) \le 0$ for all $\varphi \in \mathcal {D}$.

Combining Theorems 3.2 and 3.5 we obtain the following result.

#### Corollary 3.6

(Abstract Beurling–Deny Theorem) Let $\mathcal {H}$ be a real Hilbert space with a non-empty self-dual cone $\mathscr {P}$. Let $\mathscr {E}$ be a non-negative, symmetric, closed bilinear form with domain $\mathcal {D}$. Then, $\mathscr {E}$ is a Dirichlet form if and only if $e^{t\mathscr {L}}\varphi \ge 0$ for all $t \ge 0$ and all $\varphi \ge 0$.

### Completely Dirichlet Forms

Let $\mathscr {E}$ be a Dirichlet form on $\big (\mathcal {A}, \langle {\cdot , \cdot }\rangle _{L^2_\mathrm{KMS}(\sigma )}\big )$ with the KMS inner product specified by a faithful state $\sigma$. Here, the notion of Dirichlet form is understood with respect to the self-dual cone consisting of all positive semidefinite matrices belonging to $\mathcal {A}$; see Lemma 3.10 below. Let $\mathscr {P}_t = e^{t\mathscr {L}}$ where $\mathscr {L}$ is the semigroup generator associated to $\mathscr {E}$. Recall that the Dirichlet form $\mathscr {E}$ is said to be *completely Dirichlet* in case for each *t*, $\mathscr {P}_t$ is completely positive.

The condition that $\mathscr {E}$ be completely Dirichlet may be expressed in terms of $\mathscr {E}$ itself, permitting one to check the property directly from a specification of $\mathscr {E}$.

For $m\in {{\mathbb {N}}}$, let $E_{ij}$ denote the matrix whose (*i*, *j*)-entry is 1, with all other entries being 0. Alternatively, $E_{ij}$ represents the linear transformation taking $\mathbf{e}_j$ to $\mathbf{e}_i$, while annihilating $\mathbf{e}_k$ for $k\ne j$. (Here $\{\mathbf{e}_1,\dots ,\mathbf{e}_m\}$ is the standard orthonormal basis of ${{\mathbb {C}}}^m$.) It follows that $E_{ij}E_{k\ell } = \delta _{jk}E_{i\ell }$. The general element of $\mathcal {A}\otimes {{\mathbb {M}}}_m({{\mathbb {C}}})$ can be written as

3.6 $$\begin{aligned} \mathbf{A} = \sum _{i,j=1}^m A_{ij}\otimes E_{ij} \end{aligned}$$

where each $A_{ij}\in \mathcal {A}$. With $\tau _m$ denoting the normalized trace on ${{\mathbb {M}}}_m({{\mathbb {C}}})$, the state $\sigma \otimes \tau _m$ on $\mathcal {A}\otimes {{\mathbb {M}}}_m({{\mathbb {C}}})$ is defined by

$$\begin{aligned} \sigma \otimes \tau _m(\mathbf{A}) := \frac{1}{m} \sum _{j=1}^m \sigma (A_{jj}) \ , \end{aligned}$$

where $\mathbf{A}$ is given by (3.6). The corresponding KMS inner product on $\mathcal {A}\otimes {{\mathbb {M}}}_m({{\mathbb {C}}})$ is denoted $\langle \cdot ,\cdot \rangle _{L^2_\mathrm{KMS}(\sigma \otimes \tau _m)}$. One readily checks that for $\mathbf{A},\mathbf{B}\in \mathcal {A}\otimes {{\mathbb {M}}}_m({{\mathbb {C}}})$,

$$\begin{aligned} \langle \mathbf{B},\mathbf{A}\rangle _{L^2_\mathrm{KMS}(\sigma \otimes \tau _m)} = \frac{1}{m} \sum _{i,j=1}^m \langle { B_{ij}, A_{ij} }\rangle _{L^2_\mathrm{KMS}(\sigma )} \ . \end{aligned}$$

Define $\mathscr {P}_t^{(m)}$ on $\mathcal {A}\otimes {{\mathbb {M}}}_m({{\mathbb {C}}})$ by

3.7 $$\begin{aligned} \mathscr {P}_t^{(m)} \mathbf{A} = \sum _{i,j=1}^m \mathscr {P}_t A_{ij}\otimes E_{ij} \end{aligned}$$

where $\mathbf{A}$ is given by (3.6). One then computes

$$\begin{aligned} - \frac{\mathrm{d}}{\mathrm{d}t} \langle \mathbf{A},\mathscr {P}_t^{(m)} \mathbf{A} \rangle _{L^2_\mathrm{KMS} (\sigma \otimes \tau _m)}\bigg |_{t=0} = - \frac{1}{m} \sum _{i,j=1}^m \langle A_{ij}, \mathscr {L}A_{ij}\rangle _{L^2_\mathrm{KMS}(\sigma )} = \frac{1}{m} \sum _{i,j=1}^m\mathscr {E}(A_{ij}, A_{ij})\ . \end{aligned}$$

Thus, we define $\mathscr {E}^{(m)}$ on $\big (\mathcal {A}\otimes {{\mathbb {M}}}_m({{\mathbb {C}}}), \langle \cdot ,\cdot \rangle _{L^2_\mathrm{KMS}(\sigma \otimes \tau _m)}\big )$ by

3.8 $$\begin{aligned} \mathscr {E}^{(m)} (\mathbf{A},\mathbf{A}) = \frac{1}{m} \sum _{i,j=1}^m\mathscr {E}(A_{ij}, A_{ij}) \end{aligned}$$

where $\mathbf{A}$ is given by (3.6). In view of Corollary 3.6, $\mathscr {E}$ is completely Dirichlet if and only if for each $m\in {{\mathbb {N}}}$, $\mathscr {E}^{(m)}$ is Dirichlet.

A QMS $(\mathscr {P}_t)_t$ is not only completely positive; it also satisfies $\mathscr {P}_t{\mathbf{1}}= {\mathbf{1}}$ for all *t*. This too may be expressed in terms of the Dirichlet form $\mathscr {E}$: A Dirichlet form $\mathscr {E}$ is *conservative* in case $\mathscr {E}(A, {\mathbf{1}}) = 0$ for all $A \in \mathcal {A}$, and one readily sees that this is equivalent to the condition that $\mathscr {P}_t{\mathbf{1}}= {\mathbf{1}}$ for all *t*.

### Moreau Decomposition with Respect to the Cone of Positive Matrices

Let ${{\mathbb {H}}}_n({{\mathbb {C}}})$ denote the set of self-adjoint $n \times n$ matrices, which contains a distinguished pointed cone $\mathscr {P}$, namely the cone of positive semidefinite matrices *A*. If we equip ${{\mathbb {H}}}_n({{\mathbb {C}}})$ with the Hilbert–Schmidt inner product $\langle X,Y\rangle = {{\,\mathrm{Tr}\,}}[X Y]$, then $\mathscr {P}$ is self-dual: for $X\in {{\mathbb {H}}}_n({{\mathbb {C}}})$, $\langle X,A\rangle \ge 0$ for all $A\in \mathscr {P}$ if and only if $\langle v,Xv\rangle \ge 0$ for all $v \in {{\mathbb {C}}}^n$, as one sees by considering rank one projections and using the spectral theorem.

The next result characterizes the Moreau decomposition in $({{\mathbb {H}}}_n({{\mathbb {C}}}),\langle {\cdot ,\cdot }\rangle )$ in spectral terms. For $X\in {{\mathbb {H}}}_n({{\mathbb {C}}})$, there is the *spectral decomposition* $X = X_{(+)} - X_{(-)}$ where

3.9 $$\begin{aligned} X_{(+)} = X{\mathbf{1}}_{(0,\infty )}(X) \quad \mathrm{and}\quad X_{(-)} = -X{\mathbf{1}}_{(-\infty ,0)}(X) \ . \end{aligned}$$

#### Theorem 3.7

(Moreau decomposition for Hilbert–Schmidt) Let $\mathcal {H}$ be ${{\mathbb {H}}}_n({{\mathbb {C}}})$ equipped with the Hilbert–Schmidt inner product, and let $\mathscr {P}$ be the cone of positive semidefinite matrices. Then the spectral decomposition of $X\in \mathcal {H}$ coincides with the decomposition of *X* into its positive and negative parts with respect to $\mathscr {P}$.

#### Proof

Let $X \in {{\mathbb {H}}}_n({{\mathbb {C}}})$, and let $X = X_+-X_-$ be the decomposition determined by $\mathscr {P}$. Then, for *v* in the range of $X_+$, we have $X_+ - \epsilon |v\rangle \langle v| \in \mathscr {P}$ for all sufficiently small $\epsilon > 0$. Therefore,

$$\begin{aligned} \Vert X_-\Vert ^2 = \Vert X - X_+ \Vert ^2 \le \Vert X - (X_+ - \epsilon |v\rangle \langle v|)\Vert ^2 = \Vert X_-\Vert ^2 -2\epsilon \langle v, X_-v\rangle + \epsilon ^2\Vert v\Vert ^2\ . \end{aligned}$$

It follows that $\langle v, X_-v\rangle \le 0$, but since $X_-\in \mathscr {P}$, this yields $\langle v, X_-v\rangle = 0$. Hence the range of $X_+$ lies in the null-space of $X_-$, so that $X_-X_+ =0$. Taking the adjoint, we find that $X_+ X_- =0$. Therefore, $X_-$ and $X_+$ commute with each other, and hence with *X*. Thus, the projectors onto the ranges of $X_+$ and $X_-$ are both spectral projectors of *X*. Since $X = X_+ - X_-$ it follows that $X_+ = X_{(+)}$ and $X_- = X_{(-)}$. $\square$

The situation is more interesting for other inner products on ${{\mathbb {H}}}_n({{\mathbb {C}}})$. Let $\sigma$ be an invertible density matrix. For $s\in [0,1]$, let $\langle \cdot , \cdot \rangle _s$ be the inner product on ${{\mathbb {M}}}_n({{\mathbb {C}}})$ given by $\langle A, B\rangle _s = {{\,\mathrm{Tr}\,}}[ A^*\sigma ^s B \sigma ^{1-s}]$.

#### Theorem 3.8

Let $\sigma$ be an invertible $n\times n$ density matrix that is not a multiple of the identity. Then the cone $\mathscr {P}$ of positive matrices in ${{\mathbb {H}}}_n({{\mathbb {C}}})$ is self-dual with respect to the inner product $\langle \cdot , \cdot \rangle _s$ determined by $\sigma$ if and only if $s=\frac{1}{2}$.

#### Proof

Let $X\in {{\mathbb {H}}}_n({{\mathbb {C}}})$ and $A\in \mathscr {P}$. Then $\langle X,A\rangle _s = {{\,\mathrm{Tr}\,}}[ X\sigma ^s A \sigma ^{1-s}] = {{\,\mathrm{Tr}\,}}[ (\sigma ^{1-s} X\sigma ^s) A ]$. Therefore, $\langle X,A\rangle _s \ge 0$ for all $A\in \mathscr {P}$ if and only if $\sigma ^{1-s} X\sigma ^s \in \mathscr {P}$. If $\sigma ^{1-s} X\sigma ^s \in \mathscr {P}$, then $\sigma ^{1-s} X\sigma ^s$ is self-adjoint, and hence $\sigma ^{1-s} X\sigma ^s = \sigma ^{s} X\sigma ^{1-s}$, or, what is the same, $[\sigma ^{1-2s},X] = 0$. Let $X := |v \rangle \langle v|$ with *v* chosen *not* to be an eigenvector of $\sigma$. Then for $s\ne \frac{1}{2}$, $[\sigma ^{1-2s},X] \ne 0$. Therefore, $X\in \mathscr {P}$, but $X\notin \mathscr {P}^\circ$. Hence, $\mathscr {P}$ is not self-dual when $\mathcal {H}$ is equipped with the inner product $\langle \cdot , \cdot \rangle _s$ for $s\ne \frac{1}{2}$.

One the other hand,

$$\begin{aligned} \langle X,A\rangle _{1/2} = {{\,\mathrm{Tr}\,}}[ X\sigma ^{1/2} A \sigma ^{1/2}] = {{\,\mathrm{Tr}\,}}[ (\sigma ^{1/4} X\sigma ^{1/4}) (\sigma ^{1/4}A\sigma ^{1/4}) ]\ . \end{aligned}$$

Since $\sigma$ is invertible, as *A* ranges over $\mathscr {P}$, $\sigma ^{1/4}A\sigma ^{1/4}$ ranges over $\mathscr {P}$, and so $\langle X,A\rangle _{1/2}\ge 0$ for all $A\in \mathscr {P}$ if and only if $\sigma ^{1/4} X\sigma ^{1/4} \in \mathscr {P}$. Again, since $\sigma$ is invertible, this is the case if and only if $X\in \mathscr {P}$. Hence, $\mathscr {P}$ is self-dual for $\langle \cdot , \cdot \rangle _{1/2}$, the KMS inner product. $\square$

The Moreau decomposition for the KMS scalar product can easily be obtained from Theorem 3.7 by a unitary transformation.

#### Theorem 3.9

(Moreau decomposition for KMS) Let $\sigma$ be an invertible $n\times n$ density matrix and let $X\in {{\mathbb {H}}}_n({{\mathbb {C}}})$. Then, with respect to the KMS norm on ${{\mathbb {H}}}_n({{\mathbb {C}}})$,

3.10 $$\begin{aligned} \Vert X - \sigma ^{-1/4}(\sigma ^{1/4}X\sigma ^{1/4})_{(+)} \sigma ^{-1/4}\Vert _{L^2_\mathrm{KMS}(\sigma )} \le \Vert X - A\Vert _{L^2_\mathrm{KMS}(\sigma )} \end{aligned}$$

for all $A\in \mathscr {P}$. Consequently, the positive part of *X* in the decomposition according to $\mathscr {P}$, $X_+$, is given by

3.11 $$\begin{aligned} X_+ = \sigma ^{-1/4}(\sigma ^{1/4}X\sigma ^{1/4})_{(+)} \sigma ^{-1/4}\ . \end{aligned}$$

#### Proof

The map $Y \mapsto \sigma ^{1/4}Y\sigma ^{1/4}$ is unitary from ${{\mathbb {H}}}_n({{\mathbb {C}}})$ equipped with the KMS inner product to ${{\mathbb {H}}}_n({{\mathbb {C}}})$ equipped with the Hilbert–Schmidt inner product. That is,

$$\begin{aligned} \Vert X -A\Vert _{L^2_\mathrm{KMS}(\sigma )}^2 = {{\,\mathrm{Tr}\,}}[ \sigma ^{1/4}X\sigma ^{1/4} - \sigma ^{1/4}A\sigma ^{1/4}]^2 \end{aligned}$$

for $X, A \in {{\mathbb {H}}}_n({{\mathbb {C}}})$. By Theorem 3.7, $\min \{ {{\,\mathrm{Tr}\,}}[ \sigma ^{1/4}X\sigma ^{1/4} - B]^2\ :\ B\in \mathscr {P}\}$ is achieved at $B = (\sigma ^{1/4}X\sigma ^{1/4})_{(+)}$. $\square$

We conclude the section by extending the results above to an arbitrary $*$-subalgebra $\mathcal {A}$ of ${{\mathbb {M}}}_n({{\mathbb {C}}})$. Let $\sigma$ be an invertible $n\times n$ density matrix belonging to $\mathcal {A}$.

#### Lemma 3.10

Let $\mathcal {H}$ be $\mathcal {A}_{h}$ equipped with the KMS inner product induced by $\sigma$, and let $\mathscr {P}$ be the positive matrices in ${{\mathbb {M}}}_n({{\mathbb {C}}})$, and let $\mathscr {P}_\mathcal {A}:= \mathscr {P}\cap \mathcal {A}$. Then $\mathscr {P}_\mathcal {A}$ is self-dual in $\mathcal {H}$.

#### Proof

Let $X \in \mathscr {P}_\mathcal {A}$. For any $A \in \mathscr {P}_\mathcal {A}$ we have $\sigma ^{1/2}A\sigma ^{1/2} \ge 0$, hence $\langle {X,A}\rangle _{L^2_\mathrm{KMS}(\sigma )} = {{\,\mathrm{Tr}\,}}[X \sigma ^{1/2}A\sigma ^{1/2}] \ge 0$, which shows that $X \in \mathscr {P}_\mathcal {A}^\circ$.

Conversely, suppose that $X \in \mathcal {A}_h$ belongs to $\mathscr {P}_\mathcal {A}^\circ$. For every $A \in \mathscr {P}_\mathcal {A}$ we then have ${{\,\mathrm{Tr}\,}}[X \sigma ^{1/2} A \sigma ^{1/2}] = \langle {X,A}\rangle _{L^2_\mathrm{KMS}(\sigma )} \ge 0$. Since $\sigma$ is invertible, it follows that ${{\,\mathrm{Tr}\,}}[X B] \ge 0$ for every $B \in \mathscr {P}_\mathcal {A}$. Therefore, the spectrum of *X* is non-negative, which implies that *X* belongs to $\mathscr {P}$ and hence to $\mathscr {P}_\mathcal {A}$. $\square$

#### Lemma 3.11

Let *X* be a self-adjoint element of $\mathcal {A}$. Then the decomposition of *X* with respect to $\mathscr {P}_\mathcal {A}$ is given by $X = X_+ - X_-$ where

$$\begin{aligned} X_+ := \sigma ^{-1/4}(\sigma ^{1/4}X\sigma ^{1/4})_{(+)}\sigma ^{-1/4}\quad \mathrm{and}\quad X_- := \sigma ^{-1/4}(\sigma ^{1/4}X\sigma ^{1/4})_{(-)}\sigma ^{-1/4}\ . \end{aligned}$$

#### Proof

Let *X* be a self-adjoint element of $\mathcal {A}$. Then by Theorem 3.9, $\min \{\Vert X - A\Vert _{L^2_\mathrm{KMS}(\sigma )}\ : A \in \mathscr {P}\}$ is achieved at $A = \sigma ^{-1/4}(\sigma ^{1/4}X\sigma ^{1/4})_{(+)}\sigma ^{-1/4}$, and since this belongs to $\mathcal {A}$, this same choice of *A* also achieves the minimum in $\min \{\Vert X - A\Vert _{L^2_\mathrm{KMS}(\sigma )}\ : A \in \mathscr {P}_\mathcal {A}\}$. $\square$

## Construction of Dirichlet Forms on a Finite-Dimensional von Neumann Algebra

Motivated by the results in Sects. 2 and 3 we introduce a general framework in which various gradient flow structures can be studied naturally. This setting unifies and extends several previous approaches to gradient flows, in particular for reversible Markov chains on finite spaces [32, 35], the fermionic Fokker-Planck equation [8], and Lindblad equations with detailed balance [10, 36]

While the results in Sect. 2 show that the general QMS satisfying the $\sigma$-DBC can be represented in terms of a Dirichlet form specified in terms of derivations, our applications require us to work with representations for the generator $\mathscr {L}$ in terms of “partial derivative operators” $\partial _j$ that are not simply derivations. The reason is that, to obtain functional inequalities and sharp rates of convergence to equilibrium, it will be important to obtain commutation relations of the form $[\partial _j, \mathscr {L}] = -a \partial _j$ for $a \in {{\mathbb {R}}}$. We shall demonstrate that such commutation relations may hold for the general class of representations introduced in this section, but not for the simpler representation in terms of derivations discussed in Sect. 2.

Our starting point is a finite-dimensional von Neumann algebra $\mathcal {A}$ which we may regard as a subalgebra of ${{\mathbb {M}}}_n({{\mathbb {C}}})$ for some $n\in {{\mathbb {N}}}$. On account of the finite-dimensionality of $\mathcal {A}$, there is always a tracial positive linear functional $\tau$ on $\mathcal {A}$: One choice is the normalized trace $\tau [A] = n^{-1}{{\,\mathrm{Tr}\,}}[A]$. However, if $\mathcal {A}$ is commutative (hence isomorphic to $\ell _n^\infty$), there will be many other tracial positive linear functionals — any positive measure on $\{1,\ldots , n\}$ specifies such a positive linear functional. In what follows, $\tau$ will denote any faithful positive linear functional on $\mathcal {A}$ that is tracial; i.e., such that $\tau [AB] = \tau [BA]$ for all $A,B\in \mathcal {A}$. Since $\tau$ is faithful, every state $\sigma$ on $\mathcal {A}$ can be represented as $\sigma (A) = \tau [\sigma A]$, where on the right side $\sigma \in \mathcal {A}\subseteq {{\mathbb {M}}}_n({{\mathbb {C}}})$ is the $n\times n$ density matrix belonging to $\mathcal {A}$ determined by the state $\sigma$.

The basic operation in terms of which we shall construct completely Dirichlet forms on $\mathcal {A}$ has several components.

Let $\mathcal {B}$ be another finite-dimensional von Neumann algebra with tracial state $\tau _\mathcal {B}$. A unital $*$-homomorphism $\ell$ from $(\mathcal {A},\tau )$ to $(\mathcal {B},\tau _\mathcal {B})$ is ($\tau ,\tau _\mathcal {B}$)-*compatible* in case for all $A\in \mathcal {A}$,

4.1 $$\begin{aligned} \tau _{\mathcal {B}}[\ell (A)] = \tau [A]\ . \end{aligned}$$

Equivalently, $\ell$ is $(\tau ,\tau _\mathcal {B})$-compatible in case its adjoint $\ell ^\dagger :L^2(\mathcal {B},\tau _\mathcal {B}) \rightarrow L^2(\mathcal {A},\tau )$ satisfies $\ell ^\dagger ({\mathbf{1}}_\mathcal {B}) = {\mathbf{1}}_\mathcal {A}$.

Let $0 \ne V\in \mathcal {B}$, and let $\ell$ and *r* be a pair of $(\tau ,\tau _\mathcal {B})$-compatible unital $*$-homomorphisms from $\mathcal {A}$ into $\mathcal {B}$. Then define the operator $\partial _V: \mathcal {A}\rightarrow \mathcal {B}$ by

4.2 $$\begin{aligned} \partial _V A := Vr(A) - \ell (A)V\ . \end{aligned}$$

If $\mathcal {B}= \mathcal {A}$ and both $\ell$ and *r* are the identity, this reduces to (2.11). The following Leibniz rule shows that $\partial _V$ is an $(\ell , {r})$-skew derivation.

### Lemma 4.1

(Leibniz rule for $\partial _V$) For $A, B \in \mathcal {A}$ we have

4.3 $$\begin{aligned} \partial _V (AB) = (\partial _V A ) {r}(B) + \ell (A) \partial _V B \ . \end{aligned}$$

### Proof

Since $\ell$ and ${r}$ are algebra homomorphisms,

$$\begin{aligned} \partial _V (AB)&= V {r}(AB) - \ell (AB) V \\&= \big (V {r}(A) - \ell (A) V\big ) {r}(B) + \ell (A)\big ( V {r}(B) - \ell (B) V\big ) = (\partial _VA) {r}(B) + \ell (A) \partial _V B\ , \end{aligned}$$

which is the desired identity. $\square$

### Remark 4.2

Since $\ell$ and ${r}$ are algebra $*$-homomorphisms, it follows that

4.4 $$\begin{aligned} \ell ^\dagger \big (\ell (A_1) B \ell (A_2)\big ) = A_1 \ell ^\dagger (B) A_2 \quad \text {and} \quad {r}^\dagger \big ({r}(A_1) B {r}(A_2)\big ) = A_1 {r}^\dagger (B) A_2 \end{aligned}$$

for all $A_1, A_2 \in \mathcal {A}$ and $B \in \mathcal {B}$. Moreover, $\ell ^\dagger (B)^* = \ell ^\dagger (B^*)$ and ${r}^\dagger (B)^* = {r}^\dagger (B^*)$ for all $B \in \mathcal {B}$.

Let $\sigma \in \mathcal {A}$ be the density matrix (with respect to $\tau$) of a faithful state on $\mathcal {A}$. Since $\ell$ and *r* are $(\tau ,\tau _\mathcal {B})$-compatible, $\ell (\sigma )$ and $r(\sigma )$ are density matrices (with respect to $\tau _\mathcal {B}$ on $\mathcal {B}$). The inner product that we use on $\mathcal {B}$ is a KMS inner product based on both $\ell (\sigma )$ and $r(\sigma )$ defined in terms of the *relative modular operator* $\Delta _{\ell (\sigma ),r(\sigma )}$:

4.5 $$\begin{aligned} \Delta _{\ell (\sigma ),r(\sigma )}(B) := \ell (\sigma ) B r(\sigma )^{-1}\ . \end{aligned}$$

It is easily verified that $\Delta _{\ell (\sigma ),r(\sigma )}$ is a positive operator on $L^2(\mathcal {B},\tau _\mathcal {B})$, and hence we may define an inner product on $\mathcal {B}$ through

$$\begin{aligned} \begin{aligned} \langle B_1,B_2\rangle _{L^2_\mathrm{KMS}(\mathcal {B},\ell (\sigma ),{r}(\sigma ))}&:= \langle B_1r (\sigma )^{1/2}, \Delta _{\ell (\sigma ),r(\sigma )}^{1/2}(B_2{r}(\sigma )^{1/2})\rangle _{L^2(\mathcal {B},\tau _\mathcal {B})}\\&= \tau _\mathcal {B}[ B_1^* \ell (\sigma ^{1/2}) B_2 {r}(\sigma ^{1/2})] \ . \end{aligned} \end{aligned}$$

Given a faithful state $\sigma$ on $\mathcal {A}$, $V\in \mathcal {B}$, and *two* pairs $(\ell ,{r})$ and $(\ell _*,{r}_*)$ of ($\tau ,\tau _\mathcal {B}$)-compatible $*$-homomorphisms of $\mathcal {A}$ into $\mathcal {B}$, define $\partial _V$ by (4.2), and define

$$\begin{aligned} \partial _{V^*} = V^*r_*(A) - \ell _*(A)V^* \end{aligned}$$

in accordance with (4.2), but using $V^*$, $\ell _*$ and ${r}_*$. Then define a sesquilinear form $\mathscr {E}$ on $\mathcal {A}$ by

4.6 $$\begin{aligned} \mathscr {E}(A_1,A_2) = \langle \partial _V A_1,\partial _V A_2\rangle _{L^2_\mathrm{KMS}(\mathcal {B},\ell (\sigma ),{r}(\sigma ))} + \langle \partial _{V^*} A_1,\partial _{V^*} A_2\rangle _{L^2_\mathrm{KMS}(\mathcal {B},\ell _*(\sigma ),{r}_*(\sigma ))}\ . \end{aligned}$$

Our immediate goal in this section is to determine conditions on *V*, $(\ell ,{r})$ and $(\ell _*,{r}_*)$ under which $\mathscr {E}$ is a conservative completely Dirichlet form on $\mathcal {A}$ equipped with the KMS inner product induced by $\sigma$.

It is first of all necessary that the operator $\mathscr {L}$ determined by $\mathscr {E}$ through $\mathscr {E}(A_1,A_2) = -\langle B,\mathscr {L}A\rangle _{L^2_\mathrm{KMS}(\sigma )}$ be real; i.e., $(\mathscr {L}(A))^* = \mathscr {L}A^*$. Since $\langle A_1, A_2\rangle _{L^2_\mathrm{KMS}(\sigma )} = \langle A_2^*,A_1^*\rangle _{L^2_\mathrm{KMS}(\sigma )}$ for all $A_1,A_2\in \mathcal {A}$, it is easily seen that $\mathscr {L}$ is real if and only if $\mathscr {E}(A_1,A_2) = \mathscr {E}(A_2^*,A_1^*)$ for all $A_1,A_2\in \mathcal {A}$.

### Lemma 4.3

Under the condition that for all $A_1, A_2 \in \mathcal {A}$,

4.7 $$\begin{aligned} \tau _\mathcal {B}[ V^* \ell (A_1) V {r}(A_2) ] = \tau _\mathcal {B}[ V^* {r}_{*} (A_1) V \ell _{*} (A_2) ] \ , \end{aligned}$$

we have $\mathscr {E}(A_1, A_2) = \mathscr {E}(A_2^*, A_1^*)$ for all $A_1, A_2\in \mathcal {A}$.

### Remark 4.4

One can satisfy (4.7) in a trivial way by taking $\ell$, *r*, $\ell _*$ and ${r}_*$ each to be the identity. Almost as trivially, one may take $\ell _* = {r}$ and ${r}_* = \ell$. However, we shall see that one can also satisfy (4.7) with $\ell _* = \ell$ and ${r}_* = {r}= I_\mathcal {B}$ with a non-trivial $*$-homomorphism $\ell$; see the discussion in the next section on the Clifford Dirichlet form. Other non-trivial realizations of (4.7) arise in practice.

### Proof of Lemma 4.3

We compute

4.8 $$\begin{aligned} \langle \partial _V A_1,\partial _V A_2\rangle _{L^2_\mathrm{KMS}(\mathcal {B},\ell (\sigma ),{r}(\sigma ))}= & {} \tau _\mathcal {B}[{r}(A_1^*)V^*\ell (\sigma ^{1/2})V{r}(A_2){r}(\sigma ^{1/2})] \end{aligned}$$

4.9 $$\begin{aligned}&+ \tau _\mathcal {B}[V^*\ell (A_1^*)\ell (\sigma ^{1/2})\ell (A_2)V{r}(\sigma ^{1/2})]\nonumber \\&- \tau _\mathcal {B}[{r}(A_1^*)V^*\ell (\sigma ^{1/2})\ell (A_2)V{r}(\sigma ^{1/2})]\nonumber \\&- \tau _\mathcal {B}[V^*\ell (A_1^*)\ell (\sigma ^{1/2})V{r}(A_2){r}(\sigma ^{1/2})] . \end{aligned}$$

By cyclicity of the trace $\tau _\mathcal {B}$, the homomorphism property of $\ell$ and ${r}$, and (4.7),

$$\begin{aligned} \tau _\mathcal {B}[{r}(A_1^*)V^*\ell (\sigma ^{1/2})V{r}(A_2){r}(\sigma ^{1/2})]= & {} \tau _\mathcal {B}[{r}(A_2\sigma ^{1/2}A_1^*)V^*\ell (\sigma ^{1/2})V] \\= & {} \tau _\mathcal {B}[\ell _*(A_2\sigma ^{1/2}A_1^*)V^*{r}_*(\sigma ^{1/2})V]\\= & {} \tau _\mathcal {B}[V\ell _*(A_2)\ell _*(\sigma ^{1/2})\ell _*(A_1^*)V^*{r}_*(\sigma ^{1/2})] \ . \end{aligned}$$

This shows that the quantity in (4.8) is what we obtain from the quantity in (4.9) if we replace $\ell$ by $\ell _*$, ${r}$ by ${r}_*$, *V* by $V^*$, $A_1$ by $A_2^*$, and $A_2$ by $A_1^*$. Similar computations then yield the identity

$$\begin{aligned} \langle \partial _V A_1,\partial _V A_2\rangle _{L^2_\mathrm{KMS}(\mathcal {B},\ell (\sigma ),{r}(\sigma ))} = \langle \partial _{V^*} A_2^*,\partial _{V^*} A_1^*\rangle _{L^2_\mathrm{KMS}(\mathcal {B},\ell _*(\sigma ),{r}_*(\sigma ))} \ , \end{aligned}$$

and this implies $\mathscr {E}(A_1,A_2) = \mathscr {E}(A_2^*,A_1^*)$. $\square$

Thus, the condition (4.7) suffices to ensure that the sesquilinear form $\mathscr {E}$ defined in (4.6) is real. In the rest of this section, we suppose that this condition is satisfied, and then since $\mathscr {E}$ is real, it suffices to consider its bilinear restriction to $\mathcal {A}_h$.

One further condition is required to ensure that $\mathscr {E}$ be a Dirichlet form on $\mathcal {A}_h$, and we shall see that under this same condition $\mathscr {E}$ is actually a completely Dirichlet form. The assumption is that *V* (resp. $V^*$) is an eigenvector of the relative modular operator $\Delta _{\ell (\sigma ),r(\sigma )}$ (resp. $\Delta _{\ell _*(\sigma ),r_*(\sigma )}$). Since the relative modular operator is positive, there exist $\omega , \omega _*\in {{\mathbb {R}}}$ such that

4.10 $$\begin{aligned} \Delta _{\ell (\sigma ),r(\sigma )}V = e^{-\omega } V \quad \text {and} \quad \Delta _{\ell _*(\sigma ),r_*(\sigma )}V^* = e^{-\omega _*} V^* \ . \end{aligned}$$

There are several equivalent formulations of this condition that will be useful.

### Lemma 4.5

The first condition in (4.10) is equivalent to the condition

4.11 $$\begin{aligned} \partial _V \log \sigma = \omega V \ , \end{aligned}$$

and to the condition that for all $t \in {{\mathbb {R}}}$,

4.12 $$\begin{aligned} \Delta ^t_{\ell (\sigma ),r(\sigma )} V = e^{- t \omega } V \ . \end{aligned}$$

Moreover, (4.10) implies that

4.13 $$\begin{aligned} \omega _* = - \omega . \end{aligned}$$

### Proof

Note that $( \Delta ^t_{\ell (\sigma ),r(\sigma )})_{t\in {{\mathbb {R}}}}$ is a group of linear operators on $\mathcal {B}$, and the generator $\mathscr {G}$ of this group is given by $\mathscr {G}B = \ell (\log \sigma ) B - B {r}(\log \sigma )$, thus $\mathscr {G}V = - \partial _V \log \sigma$. The equivalences thus follow from basic spectral theory.

Using (4.7) with $A_1 = \sigma$ and $A_2 = \sigma ^{-1}$, and two applications of (4.12), we obtain

$$\begin{aligned} e^{-\omega } \tau _\mathcal {B}[ V^* V]&= \tau _\mathcal {B}[ V^* \ell (\sigma ) V {r}(\sigma ^{-1}) ] = \tau _\mathcal {B}[ V \ell _*(\sigma ^{-1}) V^* {r}_*(\sigma ) ] = e^{\omega _*} \tau _\mathcal {B}[ V V^*] \ . \end{aligned}$$

Since $V \ne 0$, this yields (4.13). $\square$

We are now ready to state the main result of this section.

### Theorem 4.6

Let $\sigma$ be a faithful state on $\mathcal {A}$. Let $V\in \mathcal {B}$ and two pairs $(\ell ,{r})$ and $(\ell _*,{r}_*)$ of $(\tau ,\tau _\mathcal {B})$-compatible $*$-homomorphisms be given. Suppose also that (4.7) is satisfied, and suppose that *V* (resp. $V^*$) is an eigenvector of the relative modular operator $\Delta _{\ell (\sigma ),r(\sigma )}$ (resp. $\Delta _{\ell _*(\sigma ),r_*(\sigma )}$) satisfying (4.10). Then the sesquilinear form $\mathscr {E}: \mathcal {A}\times \mathcal {A}\rightarrow {{\mathbb {C}}}$ given by (4.6) defines a conservative completely Dirichlet form on $L_\mathrm{KMS}^2(\mathcal {A}_h,\sigma )$.

### Proof

To explain the crucial role of the assumption that *V* is an eigenvector of the relative modular operator, so that (4.10) is satisfied, we fix $V, W \in \mathcal {B}$ and (temporarily) define the operators $\partial , \partial _*: \mathcal {A}\rightarrow \mathcal {B}$ by $\partial A := V {r}(A) - \ell (A) W$ and $\partial _* A := V^* {r}_*(A) - \ell _*(A) W^*$, and set

$$\begin{aligned} \mathscr {E}(A_1,A_2) = \langle \partial A_1,\partial A_2\rangle _{L^2_\mathrm{KMS}(\mathcal {B},\ell (\sigma ),{r}(\sigma ))} + \langle \partial _* A_1,\partial _* A_2\rangle _{L^2_\mathrm{KMS}(\mathcal {B},\ell _*(\sigma ),{r}_*(\sigma ))}\ . \end{aligned}$$

We will show:

1. If $W = e^{\omega /2} \Delta ^{1/2}_{\ell (\sigma ),r(\sigma )} V$ and $W^* = e^{\omega _*/2} \Delta ^{1/2}_{\ell _*(\sigma ),r_*(\sigma )} V^*$ for some $\omega , \omega _* \in {{\mathbb {R}}}$, then $\mathscr {E}$ defines a Dirichlet form on $L_\mathrm{{KMS}}^2(\mathcal {A}_h,\sigma )$.
2. If, in addition, (4.10) holds, then $\mathscr {E}({\mathbf{1}},A) = 0$ for all $A \in \mathcal {A}_h$, hence $\mathscr {E}$ is conservative.

Consider the unitary transformation $\mathcal {U}: L_\mathrm{KMS}^2(\mathcal {A},\sigma ) \rightarrow L^2(\mathcal {A}, \tau )$ given by $\mathcal {U}A := \sigma ^{1/4} A \sigma ^{1/4}$. For brevity we write $\mathcal {T}B := \Delta ^{1/4}_{\ell (\sigma ),r(\sigma )} B = \ell (\sigma ^{1/4}) B {r}(\sigma ^{-1/4})$, and likewise, $\mathcal {T}_* B := \Delta ^{1/4}_{\ell _*(\sigma ),r_*(\sigma )} B = \ell _*(\sigma ^{1/4}) B {r}_*(\sigma ^{-1/4})$.

For $A \in \mathcal {A}_h$ we need to show that $\mathscr {E}(A_+, A_-) \le 0$. For $A_1, A_2 \in \mathcal {A}$ we have

4.14 $$\begin{aligned}&\langle \partial A_1, \partial A_2\rangle _{L^2_\mathrm{KMS}(\mathcal {B},\ell (\sigma ),{r}(\sigma ))} \nonumber \\&\quad = \tau _\mathcal {B}\Big [ {r}(\sigma ^{1/4}) \Big ( {r}(A_1^* ) V^* - W^* \ell (A_1^*) \Big ) \ell (\sigma ^{1/2}) \Big (V {r}(A_2) - \ell (A_2) W \Big ) {r}(\sigma ^{1/4}) \Big ] \nonumber \\&\quad = \tau _\mathcal {B}\Big [ \Big ( {r}(\mathcal {U}A_1^* ) (\mathcal {T}V )^* - (\mathcal {T}^{-1} W )^* \ell (\mathcal {U}A_1^*) \Big ) \Big ( ( \mathcal {T}V ) {r}(\mathcal {U}A_2) - \ell (\mathcal {U}A_2) \mathcal {T}^{-1} W \Big ) \Big ] \nonumber \\&\quad = \tau _\mathcal {B}\Big [ (\mathcal {T}V ) {r}(\mathcal {U}A_2 \mathcal {U}A_1^* ) (\mathcal {T}V )^* - (\mathcal {T}V )^* \ell (\mathcal {U}A_2) \mathcal {T}^{-1} W {r}(\mathcal {U}A_1^* ) \nonumber \\&\qquad - {r}(\mathcal {U}A_2) (\mathcal {T}^{-1} W )^* \ell (\mathcal {U}A_1^*) (\mathcal {T}V ) + (\mathcal {T}^{-1} W )^* \ell (\mathcal {U}A_1^* \mathcal {U}A_2) \mathcal {T}^{-1} W \Big ] \ . \end{aligned}$$

For $A \in \mathcal {A}_h$ we have $A_\pm = \mathcal {U}^{-1}(\mathcal {U}A)_{(\pm )}$ by Lemma 3.11, thus

$$\begin{aligned} \mathcal {U}A_+ \mathcal {U}A_- = (\mathcal {U}A)_{(+)}(\mathcal {U}A)_{(-)} = 0 \quad \text {and} \quad \mathcal {U}A_- \mathcal {U}A_+ = (\mathcal {U}A)_{(-)}(\mathcal {U}A)_{(+)} = 0 \ . \end{aligned}$$

We obtain

4.15 $$\begin{aligned} \begin{aligned} \langle \partial A_+, \partial A_-\rangle _{L^2_\mathrm{KMS}(\mathcal {B},\ell (\sigma ),{r}(\sigma ))}&= - \tau _\mathcal {B}\Big [ (\mathcal {T}V )^* \ell (\mathcal {U}A_-) \mathcal {T}^{-1} W {r}(\mathcal {U}A_+ ) \\&\qquad + (\mathcal {T}^{-1} W )^* \ell (\mathcal {U}A_+) (\mathcal {T}V ) {r}(\mathcal {U}A_-) \Big ] \ . \end{aligned} \end{aligned}$$

Since ${r}(\mathcal {U}A_{\pm }) \ge 0$, it follows that $\langle \partial A_+, \partial A_-\rangle _{L^2_\mathrm{KMS}(\mathcal {B},\ell (\sigma ),{r}(\sigma ))}\le 0$ if we can show that

4.16 $$\begin{aligned} (\mathcal {T}V)^* \ell (\mathcal {U}A_-) \mathcal {T}^{-1} W \ge 0 \quad \text {and} \quad (\mathcal {T}^{-1} W)^* \ell (\mathcal {U}A_+) (\mathcal {T}V ) \ge 0 \ . \end{aligned}$$

To show this, we make the assumption that $W = e^{\omega /2} \Delta _{\ell (\sigma ),r(\sigma )}^{1/2} V$ for some $\omega \in {{\mathbb {R}}}$. Equivalently, this means that $\mathcal {T}^{-1} W = e^{\omega /2} \mathcal {T}V$, and since $\ell (\mathcal {U}A_\pm ) \ge 0$, we obtain (4.16). This proves that $\langle \partial A_+, \partial A_-\rangle _{L^2_\mathrm{KMS}(\mathcal {B},\ell (\sigma ),{r}(\sigma ))} \le 0$.

An entirely analogous argument shows that $\langle \partial _* A_+, \partial _* A_- \rangle _{L^2_\mathrm{KMS}(\mathcal {B},\ell _*(\sigma ),{r}_*(\sigma ))} \le 0$, and this proves that $\mathscr {E}(A_+,A_-)$ is a Dirichlet form.

Observe now that $\partial {\mathbf{1}}= V - W$ and $\partial _* {\mathbf{1}}= V^* - W^*$. Thus, to conclude that $\partial {\mathbf{1}}= \partial _* {\mathbf{1}}= 0$, we need to assume that *V* is an eigenvector of $\Delta _{\ell (\sigma ),r(\sigma )}$ with eigenvalue $e^{-\omega }$, and that $V^*$ is an eigenvector of $\Delta _{\ell _*(\sigma ),r_*(\sigma )}$ with eigenvalue $e^{-\omega _*}$. It immediately follows that $\mathscr {E}({\mathbf{1}},A) = 0$ for all $A \in \mathcal {A}_h$, hence $\mathscr {E}$ is conservative.

It remains to prove that under the given conditions, $\mathscr {E}$ is completely Dirichlet. Let ${{\,\mathrm{Tr}\,}}$ be the standard trace on ${{\mathbb {M}}}_m({{\mathbb {C}}})$. Let $\mathbf{H}$ be a self-adjoint element of $\mathcal {A}\otimes {{\mathbb {M}}}_m({{\mathbb {C}}})$, and let $\mathbf{H}_+$ and $\mathbf{H}_-$ be the elements of its decomposition $\mathbf{H} = \mathbf{H}_+ - \mathbf{H}_-$ in $L^2_\mathrm{KMS}(\sigma \otimes {{\,\mathrm{Tr}\,}})$, where $\mathbf{H}_+$ and $\mathbf{H}_-$ are positive and such that $\langle \mathbf{H}_+,\mathbf{H}_-\rangle _{L^2_\mathrm{KMS}(\sigma \otimes {{\,\mathrm{Tr}\,}})} =0$.

Let ${\varvec{\sigma }} = \sum _{j=1}^m \sigma \otimes E_{jj}$ and write $\widetilde{\mathbf{H}} = \varvec{\sigma }^{1/4} \mathbf{H} \varvec{\sigma }^{1/4}$ for brevity. By Theorem 3.9, $\mathbf{H}_+ = \varvec{\sigma }^{-1/4} \widetilde{\mathbf{H}}_{(+)} \varvec{\sigma }^{-1/4}$, hence $[\mathbf{H}_+]_{ij} = \sigma ^{-1/4} [\widetilde{\mathbf{H}}_{(+)}]_{ij} \sigma ^{-1/4}$. It follows that

$$\begin{aligned} \sum _{i,j =1}^m \tau \big [ \mathcal {U}( [\mathbf{H}_+]_{ij} ) \mathcal {U}( [\mathbf{H}_-]_{ij} )\big ] = \langle {\widetilde{\mathbf{H}}_{(+)} , \widetilde{\mathbf{H}}_{(-)} }\rangle _{L^2(\tau \otimes {{\,\mathrm{Tr}\,}})} = 0 \ . \end{aligned}$$

Using this identity, (4.15) with $V = W$ yields

$$\begin{aligned}&\sum _{i, j = 1}^m \langle \partial [\mathbf{H}_+]_{ij}, \partial [\mathbf{H}_-]_{ij}\rangle _{L^2_\mathrm{KMS}(\mathcal {B},\ell (\sigma ),{r}(\sigma ))} \\&\quad = - \sum _{i, j = 1}^m \tau _\mathcal {B}\Big [ V^* \ell (\mathcal {U}[\mathbf{H}_-]_{ij}) V {r}(\mathcal {U}[\mathbf{H}_+]_{ij}) + V^* \ell (\mathcal {U}[\mathbf{H}_+]_{ij}) V {r}(\mathcal {U}[\mathbf{H}_-]_{ij}) \Big ] \\&\quad = - \sum _{i, j = 1}^m \tau _\mathcal {B}\Big [ V^* \ell ([\widetilde{\mathbf{H}}_{(-)}]_{ij}) V {r}([\widetilde{\mathbf{H}}_{(+)}]_{ij}) + V^* \ell ([\widetilde{\mathbf{H}}_{(+)}]_{ij}) V {r}([\widetilde{\mathbf{H}}_{(-)}]_{ij}) \Big ] \ \\&\quad = - \tau _\mathcal {B}\otimes {{\,\mathrm{Tr}\,}}\Big [ (V \otimes {\mathbf{1}}_m)^* {\ell } (\widetilde{\mathbf{H}}_{(-)}) (V \otimes {\mathbf{1}}_m) {{r}} (\widetilde{\mathbf{H}}_{(+)}) + (V \otimes {\mathbf{1}}_m)^* {\ell } (\widetilde{\mathbf{H}}_{(+)}) (V \otimes {\mathbf{1}}_m) {{r}} (\widetilde{\mathbf{H}}_{(-)}) \Big ], \end{aligned}$$

where ${\mathbf{1}}_m$ denotes the identity matrix in ${{\mathbb {M}}}_m({{\mathbb {C}}})$, and in the last line, we simply write $\ell$ and ${r}$ to denote their canonical extensions $\ell \otimes I$ and ${r}\otimes I$. Since ${{r}} (\widetilde{\mathbf{H}}_{(\pm )}) \ge 0$ and $(V \otimes I)^* {\ell } (\widetilde{\mathbf{H}}_{(\mp )}) (V \otimes {\mathbf{1}}_m) \ge 0$, it is now evident that the right-hand side is non-positive. An analogous argument applies if we replace $\partial$ by $\partial _*$, and therefore,

$$\begin{aligned} \mathscr {E}^{(m)}(\mathbf{H}_+, \mathbf{H}_-) = \sum _{i,j = 1}^m \mathscr {E}([\mathbf{H}_+]_{ij}, [\mathbf{H}_-]_{ij}) \le 0 \ . \end{aligned}$$

In summary, this proves that $\mathscr {E}^{(m)}$ is a Dirichlet form for all $m\in {{\mathbb {N}}}$, and hence that $\mathscr {E}$ is completely Dirichlet. $\square$

Evidently, the sum of a finite set of conservative completely Dirichlet forms on $\mathcal {A}$ is a conservative completely Dirichlet form. Thus, we may construct a large class of conservative completely Dirichlet forms by taking sums of forms of the type considered in Theorem 4.6. In the remainder of this section, we consider such a conservative, completely Dirichlet form and the associated QMS $\mathscr {P}_t$.

It will be convenient going forward to streamline our notation. In the rest of this section we are working in the framework specified as follows:

### Definition 4.7

Let $\mathcal {A}$ be a finite-dimensional von Neumann algebra $\mathcal {A}$ endowed with a faithful tracial positive linear functional $\tau$. A *differential structure* on $\mathcal {A}$ consists of the following:

1. A finite index set $\mathcal {J}$, and for each $j\in \mathcal {J}$, a finite dimensional von Neumann algebra $\mathcal {B}_j$ endowed with a faithful tracial positive linear functional $\tau _j$.
2. For each $j\in \mathcal {J}$, a pair $(\ell _j,{r}_j)$ of unital $*$-homomorphisms from $\mathcal {A}$ to $\mathcal {B}_j$ such that for each $A\in \mathcal {A}$ and each $j\in \mathcal {J}$, $\tau _j(\ell _j(A)) = \tau _j({r}_j(A)) = \tau (A)$, and a non-zero $V_j\in \mathcal {B}_j$.
3. It is further required that for each $j\in \mathcal {J}$, there is a unique $j^*$ such that $V_j^* = V_{j^*}$, hence $\{ V_j \}_{j \in \mathcal {J}} = \{ V_j^* \}_{j \in \mathcal {J}}$ and $\mathcal {B}_{j^*} = \mathcal {B}_j$. Moreover, for $j\in \mathcal {J}$ and $A_1, A_2 \in \mathcal {A}$,
  4.17 $$\begin{aligned} \tau _j[ V_j^* \ell _j (A_1) V_j {r}_j(A_2) ] = \tau _j[ V_j^* {r}_{j^*}(A_1) V_j \ell _{j^*} (A_2) ] \ . \end{aligned}$$
4. An invertible density matrix $\sigma \in {{\mathfrak {P}}}_+$, such that, for each $j\in \mathcal {J}$, $V_j$ is an eigenvector of the relative modular operator $\Delta _{\ell _j(\sigma ),{r}_j(\sigma )}$ on $\mathcal {B}_j$ with
  4.18 $$\begin{aligned} \Delta _{\ell _j(\sigma ),{r}_j(\sigma )}(V_j) = e^{-\omega _j}V_j \end{aligned}$$
  for some $$\omega _j \in {{\mathbb {R}}}$$.

Then for each $j\in \mathcal {J}$, we define the linear operator $\partial _j: \mathcal {A}\rightarrow \mathcal {B}_j$ by

4.19 $$\begin{aligned} \partial _{j} A := V_j {r}_j(A) - \ell _j(A) V_j \end{aligned}$$

for $A \in \mathcal {A}$, and set

$$\begin{aligned} \nabla A := (\partial _j A)_{j \in \mathcal {J}} \in \mathcal {B}\ , \qquad \mathcal {B}=\prod _{j \in \mathcal {J}} \mathcal {B}_j\ . \end{aligned}$$

We refer to $\nabla A$ as the gradient of *A*, or derivative of *A*, with respect to the differential structure on $\mathcal {A}$ defined above. We will denote the differential structure by the triple $(\mathcal {A}, \nabla , \sigma )$.

For $s \in [0,1]$ we endow $\mathcal {B}_j$ with the inner product

$$\begin{aligned} \langle {B_1, B_2}\rangle _{s,j} := \tau _j[B_1^* \ell _j(\sigma ^s) B_2 {r}_j(\sigma ^{1-s})] \ . \end{aligned}$$

The most relevant case for our purposes is $s = \frac{1}{2}$, in which case we write

$$\begin{aligned} \langle {B_1, B_2}\rangle _{L_{\mathrm{KMS}, j}^{2}(\sigma )} := \langle {B_1, B_2}\rangle _{1/2,j} \ . \end{aligned}$$

It follows immediately from Theorem 4.6 that

4.20 $$\begin{aligned} \mathscr {E}(A_1,A_2) := \sum _{j \in \mathcal {J}} \langle {\partial _{j} A_1, \partial _{j} A_2}\rangle _{L_{\mathrm{KMS}, j}^{2}(\sigma )} \end{aligned}$$

is a conservative completely Dirichlet form on $L^2_\mathrm{KMS}(\mathcal {A}_h, \sigma )$.

### Remark 4.8

As we have seen earlier in this section, *(3)* ensures that the sesquilinear form $\mathscr {E}$ defined by (4.20) is real and leads to the symmetry condition (4.13), and then *(4)* ensures that $\mathscr {E}$ is completely Dirichlet.

Having the gradient $\nabla$ at our disposal, we can define a corresponding divergence operator by trace duality. For $\mathbf {B}= (B_j)_{j\in \mathcal {J}} \in \mathcal {B}$ we shall use the notation

4.21 $$\begin{aligned} {{\,\mathrm{div}\,}}\mathbf {B}= - \sum _{j\in \mathcal {J}} \partial _j^\dagger B_j \ . \end{aligned}$$

### Proposition 4.9

Let $s \in [0,1]$. The adjoint of the differential operator $\partial _j : (\mathcal {A},\langle {\cdot ,\cdot }\rangle _s) \rightarrow (\mathcal {B}_j,\langle {\cdot ,\cdot }\rangle _{s,j})$ is given by

4.22 $$\begin{aligned} \partial _{j,\sigma }^{\dagger ,(s)} B = e^{-s\omega _j} {r}_j^\dagger (V_j^*B) - e^{(1-s)\omega _j} \ell _j^\dagger (B V_j^*) \ . \end{aligned}$$

In particular, the adjoint of the operator $\partial _j : L_\mathrm{KMS }^2(\mathcal {A},\sigma ) \rightarrow L_{\mathrm{KMS},j}^2(\mathcal {B}_j,\sigma )$ is given by

4.23 $$\begin{aligned} \partial _{j,\sigma }^\dagger B = e^{-\omega _j/2} {r}_j^\dagger (V_j^*B) - e^{\omega _j/2} \ell _j^\dagger (B V_j^*) \end{aligned}$$

for $$B \in \mathcal {B}_j$$.

### Proof

For $A \in \mathcal {A}$ we obtain using (4.4) and (4.12),

$$\begin{aligned}&\langle {\partial _j A , B}\rangle _{s,j} \\&\quad = \tau _j\big [ \big (V_j {r}_j(A) - \ell _j(A) V_j\big )^* \ell _j(\sigma ^{s}) B {r}_j(\sigma ^{1-s})\big ] \\&\quad = \tau _j\big [ {r}_j(A)^*V_j^* \ell _j(\sigma ^{s}) B {r}_j(\sigma ^{1-s}) - \ell _j(A)^* \ell _j(\sigma ^{s}) B {r}_j(\sigma ^{1-s}) V_j^* \big ] \\&\quad = \tau \big [ A^* {r}_j^\dagger \big (V_j^* \ell _j(\sigma ^{s}) B {r}_j(\sigma ^{1-s})\big ) - A^* \ell _j^\dagger \big ( \ell _j(\sigma ^{s}) B {r}_j(\sigma ^{1-s}) V_j^*\big ) \big ] \\&\quad = \tau \Big [ A^* \sigma ^{s} \Big ( {r}_j^\dagger \big ( {r}_j(\sigma ^{-s})V_j^* \ell _j(\sigma ^{s}) B \big ) - \ell _j^\dagger \big ( B {r}_j(\sigma ^{1-s}) V_j^* \ell _j(\sigma ^{s-1})\big ) \Big ) \sigma ^{1-s} \Big ] \\&\quad = \tau \big [ A^* \sigma ^{s} \big ( e^{-s\omega _j} {r}_j^\dagger (V_j^* B) - e^{(1-s)\omega _j} \ell _j^\dagger (B V_j^*) \big ) \sigma ^{1-s} \big ] \\&\quad = \langle {A, e^{-s\omega _j} {r}_j^\dagger (V_j^* B) - e^{(1-s)\omega _j} \ell _j^\dagger (B V_j^*) }\rangle _s \ , \end{aligned}$$

which proves (4.22). $$\square $$

The following result provides an explicit expression for $\mathscr {L}$.

### Proposition 4.10

The operator $\mathscr {L}$ associated to the Dirichlet form (4.20) is given by

$$\begin{aligned} \mathscr {L}A&= \sum _{j \in \mathcal {J}} e^{-\omega _j/2} {r}_j^\dagger \Big ( - {r}_j(A) V_j^* V_j + 2 V_j^* \ell _j(A) V_j - V_j^* V_j {r}_j(A) \Big )\\&= \sum _{j \in \mathcal {J}} e^{\omega _j/2} \ell _j^\dagger \Big (V_j {r}_j(A) V_j^* - \ell _j(A) V_j V_j^* \Big ) - e^{-\omega _j/2} {r}_j^\dagger \Big (V_j^* V_j {r}_j(A) - V_j^*\ell _j(A) V_j\Big ) \end{aligned}$$

for $A \in \mathcal {A}$. Its Hilbert space adjoint with respect to $L^2(\mathcal {A}, \tau )$ is given by

$$\begin{aligned} \mathscr {L}^\dagger \rho&= \sum _{j \in \mathcal {J}} e^{-\omega _j/2} \Big ( - {r}_j^\dagger \big ({r}_j(\rho ) V_j^* V_j \big ) + 2 \ell _j^\dagger \big (V_j {r}_j(\rho ) V_j^*\big ) - {r}_j^\dagger \big ( V_j^* V_j {r}_j(\rho ) \big ) \Big )\\&= \sum _{j \in \mathcal {J}} e^{\omega _j/2} \Big ( {r}_j^\dagger \big (V_j^* \ell _j(\rho ) V_j\big ) - \ell _j^\dagger \big (\ell _j(\rho ) V_j V_j^* \big ) \Big )\\&\quad - e^{-\omega _j/2} \Big ( {r}_j^\dagger \big (V_j^* V_j {r}_j(\rho ) \big ) - \ell _j^\dagger \big ( V_j {r}_j(\rho ) V_j^* \big ) \Big ) \end{aligned}$$

for $$\rho \in \mathcal {A}$$.

### Proof

Using Proposition 4.9 we obtain

$$\begin{aligned} \mathscr {L}A&= - \sum _{j \in \mathcal {J}} \partial _{j,\sigma }^\dagger \partial _j A \\&= - \sum _{j \in \mathcal {J}} e^{-\omega _j/2} {r}_j^\dagger \big (V_j^*\partial _j A\big ) - e^{\omega _j/2} \ell _j^\dagger \big ((\partial _j A) V_j^*\big )\\&= - \sum _{j \in \mathcal {J}} e^{-\omega _j/2} {r}_j^\dagger \big (V_j^* V_j {r}_j(A) - V_j^*\ell _j(A) V_j\big ) - e^{\omega _j/2} \ell _j^\dagger \big (V_j {r}_j(A) V_j^* - \ell _j(A) V_j V_j^* \big ) \ , \end{aligned}$$

which yields the second expression for $\mathscr {L}$. The first expression is obtained using (4.17) and the fact that $\omega _{j^*} = - \omega _j$. The formulas for $\mathscr {L}^\dagger$ follow by direct computation. $\square$

The following result is an immediate consequence.

### Proposition 4.11

We have

$$\begin{aligned} {{\,\mathrm{\mathsf {Ker}}\,}}(\mathscr {L}) = {{\,\mathrm{\mathsf {Ker}}\,}}(\nabla ) \quad \text {and} \quad {{\,\mathrm{\mathsf {Ran}}\,}}(\mathscr {L}^\dagger ) = {{\,\mathrm{\mathsf {Ran}}\,}}({{\,\mathrm{div}\,}}) \ . \end{aligned}$$

### Proof

The identity $\mathscr {L}A = - \sum _{j \in \mathcal {J}} \partial _{j,\sigma }^\dagger \partial _j A$ implies that ${{\,\mathrm{\mathsf {Ker}}\,}}(\nabla ) \subseteq {{\,\mathrm{\mathsf {Ker}}\,}}(\mathscr {L})$. The reverse inclusion follows from the identity $- \langle {\mathscr {L}A, A}\rangle _{L_\mathrm{KMS}^{2}(\sigma )} = \sum _{j \in \mathcal {J}} \langle {\partial _j A, \partial _j A}\rangle _{L_{\mathrm{KMS}, j}^{2}(\sigma )}$. The identification of the ranges is a consequence of duality. $\square$

### Proposition 4.12

For $s \in [0,1]$ and $A_1, A_2 \in \mathcal {A}$ we have the identity

$$\begin{aligned} - \langle {\mathscr {L}A_1, A_2}\rangle _s = \sum _{j \in \mathcal {J}} e^{(s-\frac{1}{2})\omega _j} \langle {\partial _j A_1, \partial _j A_2}\rangle _{s,j} \ . \end{aligned}$$

Consequently, the operator $\mathscr {L}$ is self-adjoint with respect to $\langle {\cdot , \cdot }\rangle _s$ for all $s \in [0,1]$, and in particular, the detailed balance condition holds in the sense of Definition 2.3.

### Proof

This follows from a direct computation using (4.22). $\square$

## Examples

We provide a number of examples of conservative completely Dirichlet forms defined in the context of a differential structure on a finite-dimensional von Neumann algebra $\mathcal {A}$ equipped with a faithful state $\sigma$.

### Generators of Quantum Markov Semigroups in Lindblad Form

We have seen in Sect. 2 that generators of quantum Markov semigroups satisfying detailed balance (see Theorem 2.4) naturally fit into the framework of Sect. 4 by taking $\mathcal {A}= \mathcal {B}_j = B(\mathscr {H})$ and $\ell _j = {r}_j = I_\mathcal {A}$.

The framework also includes quantum Markov semigroups on subalgebras $\mathcal {A}$ of $B(\mathscr {H})$. In this case we set $\mathcal {B}_j = B(\mathscr {H})$, so that the situation in which $V_j \notin \mathcal {A}$ is covered. Such a situation also arises naturally in the following example.

### Classical Reversible Markov Chains in the Lindblad Framework

For $n \ge 2$, Let $\{e_1, \ldots , e_n\}$ be an orthonormal basis of ${{\mathbb {R}}}^n$ and set $E_{k p} = \,|e_k\rangle \langle e_p|\,$. Note that $E_{k p}E_{r s} = \delta _{p r} E_{ks}$ and $E_{k p}^* = E_{p k}$. We consider the algebra $\mathcal {A}\subseteq {{\mathbb {M}}}_n({{\mathbb {C}}})$ consisting of all operators that are diagonal in the basis given by the $e_i$’s:

$$\begin{aligned} \mathcal {A}= \bigg \{ \sum _{i = 1}^n \psi _i E_{ii} \ : \ \psi _1, \ldots , \psi _n \in {{\mathbb {C}}}\bigg \} \ . \end{aligned}$$

Furthermore, for each *k*, *p*, we set $\mathcal {B}_{k p} = {{\mathbb {M}}}_n({{\mathbb {C}}})$, and we endow $\mathcal {A}$ and $\mathcal {B}_{k p}$ with the usual normalized trace given by $\tau (B) = \frac{1}{n} \sum _i \langle {B e_i, e_i}\rangle$. Let $\ell _{k p} = {r}_{k p}$ be the canonical embedding from $\mathcal {A}$ into $\mathcal {B}_{k p}$. It then follows that $\ell _{k p}^\dagger (B) = {r}_{k p}^\dagger (B) = \sum _{i} \langle {Be_i,e_i}\rangle E_{ii}$.

For $k \ne p$, let $q_{k p} \ge 0$ be the transition rate of a continuous-time Markov chain on $\{1, \ldots , n\}$. We set $V_{k p} = 2^{-1/2}(q_{k p} q_{p k})^{1/4} E_{k p}$ so that $V_{k p}^* = V_{p k}$. Moreover, it is immediate to see that the identity in (4.17) holds. Fix positive weights $\pi _1, \ldots , \pi _n$. It then follows that $\sigma = \sum _i \pi _i E_{ii}$ satisfies (4.18) with $\omega _{k p} = \log (\pi _p /\pi _k)$.

By Proposition 4.10, the operator $\mathscr {L}$ associated to the Dirichlet form (4.20) is given by

$$\begin{aligned} \mathscr {L}A&= \frac{1}{2} \sum _{k \ne p} \sqrt{\frac{q_{k p} q_{p k} \pi _k}{\pi _p}} \Big ( E_{k p}^*[A, E_{k p}] + [E_{k p}^*, A] E_{k p} \Big ) \end{aligned}$$

for $A \in \mathcal {A}$. Assume now that $\pi _1, \ldots , \pi _n$ satisfy the classical detailed balance condition, i.e., $\pi _k q_{kp} = \pi _p q_{p k}$ for all *k*, *p*. Then we have

$$\begin{aligned} \mathscr {L}A = \frac{1}{2} \sum _{k \ne p} q_{p k} \Big ( E_{k p}^*[A, E_{k p}] + [E_{k p}^*, A] E_{k p} \Big ) \ . \end{aligned}$$

More explicitly,

$$\begin{aligned} \mathscr {L}\Big (\sum _i \psi _i E_{ii}\Big ) = \sum _{k,p} q_{k p} (\psi _p - \psi _k) E_{kk} \ . \end{aligned}$$

Hence, under the identification $(\psi _1, \ldots , \psi _n) \leftrightarrow \sum _{i = 1}^n \psi _i E_{ii}$, the operator $\mathscr {L}$ corresponds to the operator $\mathscr {L}_\mathrm{M}$ given by $(\mathscr {L}_\mathrm{M} \psi )_k = \sum _{p} q_{kp} (\psi _p - \psi _k)$, which is the generator of the continuous-time Markov chain on $\{1, \ldots , n\}$ with transition rates from *k* to *p* given by $q_{kp}$.

### Another Approach to Reversible Markov Chains

Let us now give an alternative way to put reversible Markov chains in the framework of this paper, which corresponds to the construction in [32, 33]. As above, let $q_{kp} \ge 0$ be the transition rate of a continuous-time Markov chain on $\{1, \ldots , n\}$, and assume that the positive weights $\pi _1, \ldots , \pi _n$ satisfy the detailed balance condition $\pi _k q_{kp} = \pi _p q_{p k}$. Let $\mathcal {J}:= \{ (k,p) \ : \ q_{kp} > 0 \}$ be the edge set of the associated graph. We consider the (non-)commutative probability spaces $(\mathcal {A},\tau )$ and $(\mathcal {B}_{kp}, \tau _{kp})$ given by

$$\begin{aligned} \mathcal {A}:= \ell _n^\infty \ , \quad \tau (A) := \sum _{i=1}^n A_i \pi _i\ , \qquad \mathcal {B}_{kp} = {{\mathbb {C}}}\ , \quad \tau _{kp}(B) := \frac{B}{2} \pi _k q_{kp} \ . \end{aligned}$$

The operators $\partial _{kp}$ are determined by $V_{kp} = 1$, $\ell _{kp} (A) = A_k$, and ${r}_{kp} (A) = A_p$ for $A \in \ell _n^\infty$. It follows that $\ell _{kp}^\dagger (B) = \frac{B}{2} q_{kp} e_k$ and ${r}_{kp}^\dagger (B) = \frac{B}{2} q_{pk} e_p$, where $e_k$ is the *k*’th unit vector in $\ell _n^\infty$. Therefore,

$$\begin{aligned} \partial _{kp} A = A_p - A_k \quad \text {and} \quad \partial _{kp}^\dagger B = \frac{B}{2} ( q_{pk} e_p - q_{kp} e_k ) \ . \end{aligned}$$

Moreover, as $\sigma = {\mathbf{1}}$ satisfies (4.18) with $\omega _{kp} = 0$, it is readily checked that this defines a differentiable structure in the sense of Definition 4.7. Using Proposition 4.10, we infer that the operator $\mathscr {L}$ is given by

$$\begin{aligned} (\mathscr {L}A)_k&= \sum _{p} q_{kp} (A_p - A_k), \end{aligned}$$

so that $\mathscr {L}$ is indeed the generator of the continuous time Markov chain with transition rates $q_{kp}$.

### The Discrete Hypercube

For a given Markov chain generator, there are different ways to write the generator in the framework of this paper, and it is often useful to represent $\mathscr {L}$ using set $\mathcal {J}$ that is smaller than in Example 5.3; see also [21]. We illustrate this for the simple random walk on the discrete hypercube $\mathcal {Q}^n = \{-1, 1\}^n$. Set $\mathcal {J}= \{1, \ldots , n\}$, and let $s_j:\mathcal {Q}^n \rightarrow \mathcal {Q}^n$ define the *j*-th coordinate swap defined by $s_j (x_1, \ldots , x_n) = (x_1, \ldots , - x_j, \ldots , x_n)$.

Consider the (non-)commutative probability spaces $(\mathcal {A},\tau )$ and $(\mathcal {B}_j, \tau _j)$ determined by

$$\begin{aligned} \mathcal {A}:= \ell ^\infty (\mathcal {Q}^n)\ , \quad \tau (A) := 2^{-n}\sum _{x \in \mathcal {Q}^n} A(x) \ , \qquad \mathcal {B}_{j} = \mathcal {A}\ , \quad \tau _{j} := \tau \ . \end{aligned}$$

Furthermore, set $\sigma = {\mathbf{1}}$ and $\omega _j = 0$. We define $V_{j} = 1$, $\ell _{j} = I$, and ${r}_j A(x) = A(s_j x)$, so that $\partial _j A(x) = A(s_j x) - A(x)$. This defines a differential structure with $\sigma = {\mathbf{1}}$. It follows that ${r}_j^\dagger = {r}_j$ and

$$\begin{aligned} \partial _j A(x) = \partial _j^\dagger A(x) = A(s_j x) - A(x)\ . \end{aligned}$$

It follows that

$$\begin{aligned} \mathscr {L}A(x) = 2\sum _{j=1}^n (A(s_j x) - A(x))\ , \end{aligned}$$

which is the discrete Laplacian on $\mathcal {Q}^n$ that generates the simple random walk.

### The Fermionic Ornstein–Uhlenbeck Equation

A non-commutative example in which it is advantageous to work with $\ell _j$ not equal to the identity, is the Fermionic Ornstein–Uhlenbeck operator, for which a non-commutative transport metric was constructed in [8]. Let $(Q_1, \ldots , Q_n)$ be self-adjoint operators on a finite-dimensional Hilbert space satisfying the *canonical anti-commutation relations* (CAR):

$$\begin{aligned} Q_i Q_j + Q_j Q_i = 2 \delta _{ij}\ . \end{aligned}$$

The *Clifford algebra* $\mathfrak {C}^n$ is the $2^n$-dimensional algebra generated by $\{Q_j\}_{j=1}^n$. Let $\Gamma : \mathfrak {C}^n\rightarrow \mathfrak {C}^n$ be the principle automorphism on $\mathfrak {C}^n$, i.e., the unique algebra homomorphism satisfying $\Gamma (Q_j) = - Q_j$ for all *j*. Let $\tau$ be the canonical trace on $\mathfrak {C}^n$, determined by $\tau (Q_1^{\alpha _1} \cdots Q_n^{\alpha _n}) := \delta _{0,|{\varvec{\alpha }}|}$ for all $\mathbf {A}= (\alpha _j)_j \in \{0,1\}^n$, where $|{\varvec{\alpha }}| := \sum _j \alpha _j$. We then set $\mathcal {J}= \{1, \ldots , n\}$, $\mathcal {A}:= \mathcal {B}_{j} := \mathfrak {C}^n$, and $\tau _{j} := \tau$. Furthermore we set $V_{j} = Q_j$, $\ell _{j} = \Gamma$, and ${r}_{j} = I$. Then $\ell _j^\dagger = \Gamma$, and the operators $\partial _j$ and $\partial _j^\dagger$ are skew-derivations given by

$$\begin{aligned} \partial _j A = Q_j A - \Gamma (A) Q_j\ , \qquad \partial _j^\dagger A = Q_j A + \Gamma (A) Q_j\ . \end{aligned}$$

Taking $\sigma = {\mathbf{1}}$ and $\omega _j = 0$ we obtain

$$\begin{aligned} \mathscr {L}A = 2\sum _{j=1}^n (Q_j A Q_j - A)\ , \end{aligned}$$

which implies that $\mathscr {L}= - 4\mathcal {N}$, where $\mathcal {N}$ is the *fermionic number operator* (see [8, 9] for more details).

### The Depolarizing Channel

This is one of the simplest non-commutative examples. Given a non-commutative probability space $(\mathcal {A}, \tau )$ and $\gamma > 0$, the generator is defined by

5.1 $$\begin{aligned} \mathscr {L}A = \gamma \big (\tau [A] {\mathbf{1}}- A\big ) \ . \end{aligned}$$

In the case where $\mathcal {A}= \mathcal {B}_j = {{\mathbb {M}}}_2({{\mathbb {C}}})$ and $\tau$ is the usual trace, this operator can be written in Lindblad form using the Pauli matrices

$$\begin{aligned} \sigma _x = \left[ \begin{array}{ll}0 &{}\quad 1 \\ 1 &{}\quad 0\end{array}\right] \ ,\qquad \sigma _y = \left[ \begin{array}{ll}0 &{}\quad -i \\ i &{}\quad 0\end{array}\right] \ ,\qquad \sigma _z = \left[ \begin{array}{ll}1 &{}\quad 0 \\ 0 &{}\quad -1\end{array}\right] \ . \end{aligned}$$

We set $V_j = \sqrt{\gamma } \sigma _j$ and $\ell _j = r_j = I_\mathcal {A}$, so that the differential operators $\partial _x, \partial _y$ and $\partial _z$ are the commutators

$$\begin{aligned} \partial _j A = \sqrt{\gamma } [\sigma _j, A] \end{aligned}$$

for $j \in \{ x, y, z\}$. This yields a differentiable structure with $\sigma = {\mathbf{1}}$ and $\omega _j = 0$, and a direct computation shows that $\mathscr {L}$ is indeed given by (5.1).

## Non-commutative Functional Calculus

Let $\mathcal {A}$ be a finite-dimensional $C^*$-algebra. Let $A, B \in \mathcal {A}$ be self-adjoint with spectral decompositions

6.1 $$\begin{aligned} A = \sum _i \lambda _i A_i\quad \text { and } \quad B = \sum _k \mu _k B_k \end{aligned}$$

for some eigenvalues $\lambda _i, \mu _k \in {{\mathbb {R}}}$ and spectral projections $A_i, B_k \in \mathcal {A}$ satisfying $A_i A_k = \delta _{ik} A_i$, $B_i B_k = \delta _{ik} B_i$, and $\sum _i A_i = \sum _k B_k = {\mathbf{1}}_{\mathcal {A}}$. For a function $\theta : {{\,\mathrm{sp}\,}}(A) \times {{\,\mathrm{sp}\,}}(B) \rightarrow {{\mathbb {R}}}$ we define $\theta (A,B) \in \mathcal {A}\times \mathcal {A}$ to be the *double operator sum*

6.2 $$\begin{aligned} \theta (A,B) = \sum _{i,k} \theta (\lambda _i,\mu _k) A_i \otimes B_k\ . \end{aligned}$$

### Remark 6.1

A systematic theory of infinite-dimensional generalizations of $\theta (A,B)$ has been developed under the name of *double operator integrals*, see, e.g., [5, 43].

Double operator sums are compatible with the usual functional calculus, in the sense that

6.3 $$\begin{aligned} \theta (f(A), g(B)) = (\theta \circ (f,g))(A,B) \end{aligned}$$

for all $f : {{\,\mathrm{sp}\,}}(A) \rightarrow {{\mathbb {R}}}$, $g : {{\,\mathrm{sp}\,}}(B) \rightarrow {{\mathbb {R}}}$ and $\theta : {{\mathbb {R}}}\times {{\mathbb {R}}}\rightarrow {{\mathbb {R}}}$. Moreover, recalling that the contraction operator has been defined in (2.16), we have

6.4 $$\begin{aligned} \theta _2(A,B) \# \big ( \theta _1(A,B) \# C\big ) = (\theta _2 \cdot \theta _1)(A,B) \# C \end{aligned}$$

The straightforward proof of these identities is left to the reader.

Let $\mathcal {I}\subseteq {{\mathbb {R}}}$ be an interval. Of particular relevance for our purposes is the special case where $\theta = \delta f : \mathcal {I}\times \mathcal {I}\rightarrow {{\mathbb {R}}}$ is the *discrete derivative* of a differentiable function $f : \mathcal {I}\rightarrow {{\mathbb {R}}}$, defined by

6.5 $$\begin{aligned} \delta f(\lambda ,\mu ) := \left\{ \begin{array}{ll} \displaystyle \frac{f(\lambda ) - f(\mu )}{\lambda -\mu }\ , \quad &{} \lambda \ne \mu \ ,\\ f'(\lambda )\ , \quad &{}\lambda = \mu \ . \end{array} \right. \end{aligned}$$

Using the contraction operator we can write the following useful chain rule:

6.6 $$\begin{aligned} f(A) - f(B) = \delta f(A,B) \# (A - B)\ . \end{aligned}$$

We can also formulate a chain rule for the operator $\partial _V$ defined in (4.2), which plays a crucial role in the sequel.

### Proposition 6.2

(Chain rule for $\partial _V$) Let $A \in \mathcal {A}_h$. For any function $f : {{\,\mathrm{sp}\,}}(A) \rightarrow {{\mathbb {R}}}$ we have

6.7 $$\begin{aligned} \partial _V f(A) = \delta f(\ell (A),{r}(A)) \# \partial _V A\ . \end{aligned}$$

### Proof

Let $A = \sum _i \lambda _i A_i$ be the spectral decomposition with eigenvalues $\lambda _i \in {{\mathbb {R}}}$ and spectral projections $A_i \in \mathcal {A}$ satisfying $A_i A_k = \delta _{ik} A_i$ and $\sum _i A_i = {\mathbf{1}}_\mathcal {A}$. Since $\ell ({\mathbf{1}}_\mathcal {A}) = {r}({\mathbf{1}}_\mathcal {A}) = {\mathbf{1}}_{\mathcal {A}}$ by assumption, it follows that $\sum _i \ell (A_i) = \sum _i {r}(A_i) = {\mathbf{1}}_{\mathcal {B}}$ for all *j*. Therefore,

$$\begin{aligned} \partial _V A&= \sum _i \lambda _i \Big (V {r}(A_i) - \ell (A_i) V\Big ) \\&= \sum _{i,k} (\lambda _k - \lambda _i) \ell (A_i) V {r}(A_k)\ . \end{aligned}$$

Consequently, since $\ell (A_p) \ell (A_i) = \ell (A_p A_i) = \delta _{pi}\ell (A_i)$ and ${r}(A_k) {r}(A_m) = \delta _{km}{r}(A_k)$,

6.8 $$\begin{aligned} \begin{aligned} \delta f(\ell (A),{r}(A)) \# \partial A&= \sum _{i,k,p,m} \delta f(\lambda _p,\lambda _m) (\lambda _k - \lambda _i) \ell (A_p A_i) V {r}(A_k A_m) \\&= \sum _{i,k} \delta f(\lambda _i,\lambda _k) (\lambda _k - \lambda _i) \ell (A_i) V {r}(A_k) \\&= \sum _{i,k} \big (f(\lambda _k) - f(\lambda _i) \big ) \ell (A_i) V {r}(A_k) \\&= \partial _V f(A) \ . \end{aligned} \end{aligned}$$

$$\square $$

### Remark 6.3

Note that the function *f* is not required to be differentiable in Proposition 6.2. In this case, $\delta f$ is not defined on the diagonal, but the second line in (6.8) shows that its diagonal value is irrelevant.

The following well-known chain rule can also be formulated in terms of $\delta f$.

### Proposition 6.4

Let $A: \mathcal {I}\rightarrow \mathcal {A}_{h}$ be differentiable on an interval $\mathcal {I}\subseteq {{\mathbb {R}}}$ and let *f* be a real-valued function on an interval containing ${{\,\mathrm{sp}\,}}(A(t))$ for all $t \in \mathcal {I}$. Then:

6.9 $$\begin{aligned} \frac{\mathrm {d}}{\mathrm {d}t}f(A(t))&= \delta f\big (A(t), A(t)\big ) \# A'(t)\ , \end{aligned}$$

6.10 $$\begin{aligned} \frac{\mathrm {d}}{\mathrm {d}t}\tau \big [f(A(t))\big ]&= \tau \big [f'(A(t))A'(t)\big ]\ . \end{aligned}$$

### Proof

The first assertion follows by passing to the limit in (6.6). The second identity follows easily using the definition of $\delta f$ and the cyclicity of the trace. $\square$

### Example 6.5

We illustrate the proposition above with a well-known computation that will be useful below. For $\rho , \sigma \in {{\mathfrak {P}}}_+(\mathcal {A})$ and $\nu \in \mathcal {A}_h$ with $\tau [\nu ] = 0$, set $\rho _t := \rho + t \nu$. It follows from (6.10) that

6.11 $$\begin{aligned} \partial _t {{\,\mathrm{Ent}\,}}_\sigma (\rho _t) = \tau [\nu (\log \rho _t - \log \sigma )] \ . \end{aligned}$$

Since $\delta \log (r,s) = \frac{\log r - \log s}{r - s} = \int _0^\infty (x + r)^{-1} (x + s)^{-1} \; \mathrm {d}x$, we have $\delta \log (R,S) = \int _0^\infty (x + R)^{-1} \otimes (x + S)^{-1} \; \mathrm {d}x$. Thus, (6.9) yields

6.12 $$\begin{aligned} \partial _t^2 {{\,\mathrm{Ent}\,}}_\sigma (\rho _t) = \int _0^\infty \tau \Big [\nu \frac{1}{x + \rho _t} \nu \frac{1}{x + \rho _t} \Big ] \; \mathrm {d}x \ . \end{aligned}$$

We finish this subsection with some useful properties of the sesquilinear form $(A,B)\mapsto \langle { A, \varphi (R,S) \# B }\rangle _{L^2(\tau )}$ on $\mathcal {A}$.

### Lemma 6.6

Let $R, S \in \mathcal {A}$ be self-adjoint and let $\varphi : {{\,\mathrm{sp}\,}}(R) \times {{\,\mathrm{sp}\,}}(S) \rightarrow {{\mathbb {R}}}_+$ be given. Then, for all $A \in \mathcal {A}$,

$$\begin{aligned} \langle { A, \varphi (R,S) \# A }\rangle _{L^2(\tau )} \ge 0\ . \end{aligned}$$

### Proof

Using the spectral decompositions $R = \sum _i \lambda _i R_i$ and $S = \sum _k \mu _k S_k$ we may write

$$\begin{aligned} \langle { A, \varphi (R,S) \# A }\rangle _{L^2(\tau )}&= \sum _{i,k} \varphi (\lambda _i,\mu _k) \tau [A^* R_i A S_k] \ . \end{aligned}$$

Since $\tau [A^* R_i A S_k] = \tau [(R_i A S_k)^*(R_i A S_k)] \ge 0$ the result follows. $\square$

### Proposition 6.7

Let $R, S \in \mathcal {A}$ be self-adjoint and suppose that $\varphi : {{\,\mathrm{sp}\,}}(R) \times {{\,\mathrm{sp}\,}}(S) \rightarrow {{\mathbb {R}}}$ is strictly positive. Then the sequilinear form

$$\begin{aligned} (A,B)\mapsto \langle { A, \varphi (R,S) \# B }\rangle _{L^2(\tau )} \end{aligned}$$

defines a scalar product on $\mathcal {A}$.

### Proof

Consider the spectral decompositions $R = \sum _i \lambda _i R_i$ and $S = \sum _k \mu _k S_k$. Using basic properties of the trace, we obtain

$$\begin{aligned} \overline{ \tau [A^* R_i B S_k ]} = \tau [(A^* R_i B S_k)^*] = \tau [ S_k B^* R_i A] = \tau [ B^* R_i A S_k] \ , \end{aligned}$$

and therefore, since $\varphi$ is real-valued,

$$\begin{aligned} \overline{ \langle { A, \varphi (R,S) \# B }\rangle _{L^2(\tau )}}&= \sum _{i,k} \varphi (\lambda _i, \mu _k) \overline{ \tau [A^* R_i B S_k ]} \\&= \sum _{i,k} \varphi (\lambda _i, \mu _k) \tau [ B^* R_i A S_k] = \langle { B, \varphi (R,S) \# A }\rangle _{L^2(\tau )}\ . \end{aligned}$$

Moreover, since $\varphi$ is strictly positive on the finite set ${{\,\mathrm{sp}\,}}(R) \times {{\,\mathrm{sp}\,}}(S)$, we have $\varphi \ge \varepsilon$ for some $\varepsilon > 0$. Thus Lemma 6.6 implies that $\langle { A, \varphi (R,S) \# A }\rangle \ge \varepsilon \Vert A \Vert _{L^2(\tau )}^2$. It follows that $\langle { A, \varphi (R,S) \# A }\rangle \ge 0$, with equality if and only if $A = 0$. $\square$

### Higher Order Expressions

In the sequel we will use versions of Propositions 6.2 and 6.4 for higher order derivatives, for which we need to introduce more notation. For $x = (x_1, \ldots , x_n) \in {{\mathbb {R}}}^n$ and $1 \le i \le m \le n$ we will use the shorthand notation $x_i^m = (x_i, x_{i+1}, \ldots , x_{m-1}, x_m)$. For a function $\varphi : {{\mathbb {R}}}^n \rightarrow {{\mathbb {R}}}$ and $j = 1, \ldots , n$ we consider the discrete derivative $\delta _j \varphi : {{\mathbb {R}}}^{n+1} \rightarrow {{\mathbb {R}}}$ defined by

6.13 $$\begin{aligned} \delta _j \varphi (x_1^{j-1}, (x_j, {{\widetilde{x}}}_j), x_{j+1}^n) := \delta \varphi (x_1^{j-1}, \cdot , x_{j+1}^n) (x_j, {\widetilde{x}}_j)\ , \end{aligned}$$

where $\delta$ denotes the discrete derivative given by (6.5). Iterating this procedure, one arrives at expressions that can be naturally encoded using rooted planar binary trees. Indeed, for a given function $\theta : {{\mathbb {R}}}\times {{\mathbb {R}}}\rightarrow {{\mathbb {R}}}$ and $x, y \in {{\mathbb {R}}}$, we writeThe left and right child in this tree correspond to the variables *x* and *y* in $\theta (x,y)$ respectively. More complicated trees are then constructed by iteratively replacing one of the children $\bullet$ by . This will correspond to discrete differentiation with respect to the respective variables, e.g.,6.146.156.16The middle expressions are valid whenever the variables are distinct. If some of the variables are equal, finite differences are to be interpreted as derivatives. For instance, if $x = y \ne z$ in (6.16), we haveIf $x = y = z$ in (6.16), then the formula above becomesThe functional calculus (6.2) generalizes naturally to functions of several variables. Let $A^{(1)}, \ldots , A^{(n)}$ be self-adjoint elements in $\mathcal {A}$ with spectral decompositions

$$\begin{aligned} A^{(k)} = \sum _i \lambda _i^{(k)} A_i^{(k)} \end{aligned}$$

for some eigenvalues $\lambda _i^{(k)} \in {{\mathbb {R}}}$ and spectral projections $A_i^{(k)} \in \mathcal {A}$ with $\sum _i A_i^{(k)} = {\mathbf{1}}_{\mathcal {A}}$. For a function $\theta : {{\,\mathrm{sp}\,}}(A^{(1)}) \times \cdots \times {{\,\mathrm{sp}\,}}(A^{(n)}) \rightarrow {{\mathbb {R}}}$ we define $\theta (A_1, \ldots ,A_n) \in \mathcal {A}^{\otimes n}$ to be the *multiple operator sum*

6.17 $$\begin{aligned} \theta (A_1, \ldots , A_n) = \sum _{i_1, \ldots , i_n} \theta \big (\lambda _{i_1}^{(1)},\ldots , \lambda _{i_n}^{(n)}\big ) A_{i_1}^{(1)} \otimes \cdots \otimes A_{i_n}^{(n)}\ . \end{aligned}$$

In the sequel we shall apply this definition to $\delta \theta$ in order to define expressions such as $\delta \theta (A,B)$. The tree notation is useful when considering generalizations of the contraction operation (2.16) to higher order tensor products. Each of the nodes that is a parent can be used to indicate the position at which an operator for contraction is inserted: e.g., we writewhere the fractions at the right-hand side are to be understood in the sense of limits if the denominator vanishes. These expressions appear naturally in the following chain rule that will be useful in Sect. 7.

#### Proposition 6.8

Let $A, B : \mathcal {I}\rightarrow \mathcal {A}_{h}$ be differentiable on an interval $\mathcal {I}\subseteq {{\mathbb {R}}}$, and let $\theta : {{\mathbb {R}}}\times {{\mathbb {R}}}\rightarrow {{\mathbb {R}}}$ be differentiable. Then:

#### Proof

We have $\partial _t \theta (A_t, B_t) = \partial _s|_{s = t} \theta (A_s, B_t) + \partial _s|_{s = t} \theta (A_t, B_s)$. Since we can write $\theta (A_t,B_s) = \sum _{k} \theta (A_t,\mu _{s,k})\otimes F_{s,k}$, where $B_s = \sum _{k} \mu _{s,k} F_{s,k}$ denotes the spectral decomposition of $B_s$, the result follows by applying (6.9) from Proposition 6.4 twice. $\square$

Higher order derivatives can also be naturally expressed in terms of trees, but since this will not be needed in the sequel, we will not go into details here.

## Riemannian Structures on the Space of Density Matrices

In this section we shall analyze a large class of Riemannian metrics on the space of density matrices. Throughout the section we fix a differentiable structure $(\mathcal {A}, \nabla , \sigma )$ in the sense of Definition 4.7. The generator of the associated quantum Markov semigroup $(\mathscr {P}_t)_t$ will be denoted by $\mathscr {L}$.

### Riemannian Structures on Density Matrices

Consider the ${{\mathbb {R}}}$-linear subspace

$$\begin{aligned} \mathcal {A}_0 := {{\,\mathrm{\mathsf {Ran}}\,}}(\mathscr {L}^\dagger ) \cap \mathcal {A}_h \ . \end{aligned}$$

We shall study Riemannian structures on relatively open subsets of ${{\mathfrak {P}}}_+$, the set of all strictly positive elements in ${{\mathfrak {P}}}$. These subsets are of the form

$$\begin{aligned} \mathscr {M}_\rho := \Big ( \rho + \mathcal {A}_0 \Big ) \cap {{\mathfrak {P}}}_+ \ , \end{aligned}$$

where $\rho \in {{\mathfrak {P}}}_+$. At each point of $\mathscr {M}_\rho$, the tangent space of $\mathscr {M}_\rho$ is thus naturally given by $\mathcal {A}_0$.

#### Remark 7.1

Of special interest is the *ergodic* case, i.e., the case where ${{\,\mathrm{\mathsf {Ker}}\,}}(\mathscr {L}) = {{\,\mathrm{lin}\,}}\{{\mathbf{1}}\}$. In this case we have $\mathcal {A}_0 = \{ A \in \mathcal {A}_h : \tau [A] = 0 \}$, and therefore $\mathscr {M}_\rho = {{\mathfrak {P}}}_+$ for all $\rho \in {{\mathfrak {P}}}_+$.

In order to define a Riemannian structure, we shall fix for each $j \in \mathcal {J}$ a function $\theta _j : [0,\infty ) \times [0,\infty ) \rightarrow {{\mathbb {R}}}$ satisfying the following properties:

#### Assumption 7.2

For $j \in \mathcal {J}$ the functions $\theta _j : [0,\infty ) \times [0,\infty ) \rightarrow {{\mathbb {R}}}$ are continuous. Moreover, on $(0,\infty ) \times (0,\infty )$, the function $\theta _j$ is $C^\infty$ and strictly positive, and we have the symmetry condition

7.1 $$\begin{aligned} \theta _j(r,s) = \theta _{j^*}(s,r) \ . \end{aligned}$$

Recalling the definition of the double operator sum in (6.2), we will use the shorthand notation

7.2 $$\begin{aligned} {\widehat{\rho }}_j&= \theta _j(\ell _j(\rho ), {r}_j(\rho )) \in \mathcal {B}_j \otimes \mathcal {B}_j\ , \qquad {\widehat{\rho }}= ({\widehat{\rho }}_j )_{j \in \mathcal {J}}\ \quad \text {for } \rho \in {{\mathfrak {P}}}\ , \end{aligned}$$

7.3 $$\begin{aligned} {\check{\rho }}_j&= \frac{1}{\theta _j}(\ell _j(\rho ), {r}_j(\rho )) \in \mathcal {B}_j \otimes \mathcal {B}_j\,,\qquad {\check{\rho }}= ({\check{\rho }}_j)_{j \in \mathcal {J}} \ \quad \text {for } \rho \in {{\mathfrak {P}}}_+ \ . \end{aligned}$$

Let us now define the class of quantum transport metrics that we are interested in. For $\rho \in {{\mathfrak {P}}}$, we define the operator $\mathscr {K}_\rho : \mathcal {A}\rightarrow \mathcal {A}$ by

7.4 $$\begin{aligned} \mathscr {K}_\rho A := - {{\,\mathrm{div}\,}}({\widehat{\rho }} \# \nabla A) = \sum _{j \in \mathcal {J}} \partial _j^\dagger ({\widehat{\rho }}_j \# \partial _j A) \ , \end{aligned}$$

where we use the vector notation ${\widehat{\rho }} \# \nabla A = ( {\widehat{\rho }}_j \# \partial _j A )_{j \in \mathcal {J}}$ and we recall that the divergence operator has been defined in (4.21). To define the Riemannian metric we need a lemma concerning the unique solvability of the continuity equation in the class of “gradient vector fields”. Therefore we need to identify the kernel and the range of the linear operator $\mathscr {K}_\rho$.

#### Lemma 7.3

(Mapping properties of $\mathscr {K}_\rho$) For $\rho \in {{\mathfrak {P}}}_+$ the operator $\mathscr {K}_\rho$ is non-negative and self-adjoint on $L^2(\mathcal {A},\tau )$. Moreover, we have

7.5 $$\begin{aligned} {{\,\mathrm{\mathsf {Ran}}\,}}(\mathscr {K}_\rho ) = {{\,\mathrm{\mathsf {Ran}}\,}}(\mathscr {L}^\dagger ) = {{\,\mathrm{\mathsf {Ran}}\,}}({{\,\mathrm{div}\,}})\ , \qquad {{\,\mathrm{\mathsf {Ker}}\,}}(\mathscr {K}_\rho ) = {{\,\mathrm{\mathsf {Ker}}\,}}(\mathscr {L}) = {{\,\mathrm{\mathsf {Ker}}\,}}(\nabla )\ . \end{aligned}$$

Furthermore, $\mathscr {K}_\rho$ is real, i.e., for $A \in \mathcal {A}$ we have $(\mathscr {K}_\rho A)^* = \mathscr {K}_\rho A^*$.

#### Proof

For $A, B \in \mathcal {A}$, Lemma 6.7 yields

$$\begin{aligned} \langle {\mathscr {K}_\rho A, B}\rangle _{L^2(\tau )}&= \sum _{j \in \mathcal {J}} \langle { {\widehat{\rho }}_j \# \partial _j A,\partial _j B }\rangle _{L^2(\tau _j)} = \sum _{j \in \mathcal {J}} \langle {\partial _j A, {\widehat{\rho }}_j \# \partial _j B }\rangle _{L^2(\tau _j)} = \langle {A, \mathscr {K}_\rho B}\rangle _{L^2(\tau )} \ , \end{aligned}$$

hence $\mathscr {K}_\rho$ is self-adjoint on $L^2(\mathcal {A}, \tau )$.

The identities ${{\,\mathrm{\mathsf {Ker}}\,}}(\mathscr {L}) = {{\,\mathrm{\mathsf {Ker}}\,}}(\nabla )$ and ${{\,\mathrm{\mathsf {Ran}}\,}}(\mathscr {L}^\dagger ) = {{\,\mathrm{\mathsf {Ran}}\,}}({{\,\mathrm{div}\,}})$ have already been proved in Proposition 4.11. Clearly, ${{\,\mathrm{\mathsf {Ker}}\,}}(\nabla ) \subseteq {{\,\mathrm{\mathsf {Ker}}\,}}(\mathscr {K}_\rho )$. To prove the opposite inclusion, we note that since $\rho \in {{\mathfrak {P}}}_+$, there exists $c > 0$ with $\theta _j|_{{{\,\mathrm{sp}\,}}(\rho )} \ge c > 0$ for all $j \in \mathcal {J}$. Lemma 6.6 implies that

$$\begin{aligned} \langle { A, \mathscr {K}_\rho A}\rangle _{L^2(\mathcal {A},\tau )} = \sum _{j \in \mathcal {J}} \langle {\partial _j A, {\widehat{\rho }}_j \# \partial _j A}\rangle _{L^2(\mathcal {B}_j,\tau _j)} \ge c \sum _{j \in \mathcal {J}} \Vert \partial _j A \Vert _{L^2(\mathcal {B}_j,\tau _j)}^2 \ , \end{aligned}$$

from which we infer that ${{\,\mathrm{\mathsf {Ker}}\,}}(\mathscr {K}_\rho )\subseteq {{\,\mathrm{\mathsf {Ker}}\,}}(\nabla )$. This proves the second identity in (7.5), and the nonnegativity of $\mathscr {K}_\rho$ follows as well. The first identity in (7.5) follows using elementary linear algebra, since the self-adjointness of $\mathscr {K}_\rho$ in $L^2(\mathcal {A}, \tau )$ yields

$$\begin{aligned} {{\,\mathrm{\mathsf {Ran}}\,}}(\mathscr {K}_\rho ) = ( {{\,\mathrm{\mathsf {Ker}}\,}}(\mathscr {K}_\rho ) )^\perp = ({{\,\mathrm{\mathsf {Ker}}\,}}(\nabla ))^\perp = {{\,\mathrm{\mathsf {Ran}}\,}}({{\,\mathrm{div}\,}})\ . \end{aligned}$$

To prove that $\mathscr {K}_\rho$ preserves self-adjointness, we consider the spectral decomposition $\rho = \sum _k \lambda _k E_k$, and write $\theta _j^{km} := \theta _j(\lambda _k, \lambda _m)$ for brevity. We have

7.6 $$\begin{aligned}&\langle {B,\mathscr {K}_\rho A^*}\rangle _{L^2(\tau )}\nonumber \\&\quad = \sum _{j \in \mathcal {J}} \tau _j[(\partial _j B)^* {\widehat{\rho }}_j \# \partial _j A^*]\nonumber \\&\quad = \sum _{j \in \mathcal {J}}\sum _{k,m} \theta _j^{km} \tau _j\Big [\Big ( V_j {r}_j(B) - \ell _j(B) V_j \Big )^* \ell _j(E_k) \Big (V_j {r}_j(A^*) - \ell _j(A^*) V_j\Big ) {r}_j(E_m)\Big ]\nonumber \\&\quad = \sum _{j \in \mathcal {J}}\sum _{k,m} \theta _j^{km}\tau _j \Big [ {r}_j(B^*) V_j^* \ell _j(E_k) V_j {r}_j(A^*) {r}_j(E_m) - V_j^*\ell _j(B^*) \ell _j(E_k) V_j {r}_j(A^*) {r}_j(E_m)\nonumber \\&\qquad - {r}_j(B^*) V_j^* \ell _j(E_k) \ell _j(A^*) V_j {r}_j(E_m) + V_j^* \ell _j(B^*) \ell _j(E_k) \ell _j(A^*) V_j {r}_j(E_m) \Big ] \nonumber \\&\quad = \sum _{j \in \mathcal {J}}\sum _{k,m} \theta _j^{km}\tau _j \Big [ V_j^* \ell _j(E_k) V_j {r}_j(A^* E_m B^*) - V_j^* \ell _j(B^* E_k) V_j {r}_j(A^* E_m) \nonumber \\&\qquad - V_j^* \ell _j(E_k A^*) V_j {r}_j(E_m B^*) + V_j^* \ell _j(B^* E_k A^*) V_j {r}_j(E_m) \Big ]. \end{aligned}$$

On the other hand,

$$\begin{aligned}&\langle {B,(\mathscr {K}_\rho A)^*}\rangle _{L^2(\tau )}\\&\quad = \sum _{j \in \mathcal {J}} \tau _j[(\partial _j B^*) ({\widehat{\rho }}_j \# \partial _j A)^*]\\&\quad = \sum _{j \in \mathcal {J}}\sum _{k,m} \theta _j^{km} \tau _j\Big [\Big ( V_j {r}_j(B^*) - \ell _j(B^*) V_j \Big ) {r}_j(E_m) \Big ( V_j {r}_j(A) - \ell _j(A) V_j \Big )^*\ell _j(E_k)\Big ]\\&\quad = \sum _{j \in \mathcal {J}}\sum _{k,m} \theta _j^{km} \tau _j\Big [ V_j {r}_j(B^*) {r}_j(E_m) {r}_j(A^*)V_j^*\ell _j(E_k) - V_j {r}_j(B^*) {r}_j(E_m) V_j^* \ell _j(A^*) \ell _j(E_k) \\&\qquad - \ell _j(B^*) V_j {r}_j(E_m) {r}_j(A^*) V_j^*\ell _j(E_k) + \ell _j(B^*) V_j {r}_j(E_m) V_j^*\ell _j(A^*) \ell _j(E_k)\Big ] \\&\quad = \sum _{j \in \mathcal {J}}\sum _{k,m} \theta _j^{km} \tau _j\Big [ V_j^* \ell _j(E_k) V_j {r}_j(B^* E_m A^*) - V_j^* \ell _j(A^* E_k)V_j {r}_j(B^* E_m) \\&\qquad - V_j^* \ell _j(E_k B^*) V_j {r}_j(E_m A^*) + V_j^* \ell _j(A^* E_k B^*) V_j {r}_j(E_m) \Big ] \ . \end{aligned}$$

Thus, using (4.17), and then changing *j* by $j^*$ and using that $\theta _j^{km} = \theta _{j^*}^{mk}$ by Assumption 7.2, we obtain

$$\begin{aligned}&\langle {B,(\mathscr {K}_\rho A)^*}\rangle _{L^2(\tau )} \\&\quad = \sum _{j \in \mathcal {J}}\sum _{k,m} \theta _j^{km} \tau _j\Big [ V_j \ell _{j^*}(B^* E_m A^*) V_j^* {r}_{j^*}(E_k) - V_j \ell _{j^*}(B^* E_m) V_j^* {r}_{j^*}(A^* E_k) \\&\qquad - V_j \ell _{j^*}(E_m A^*) V_j^* {r}_{j^*}(E_k B^*) + V_j \ell _{j^*}(E_m) V_j^* {r}_{j^*}(A^* E_k B^*) \Big ] \\&\quad = \sum _{j \in \mathcal {J}}\sum _{k,m} \theta _j^{mk} \tau _j \Big [ V_j^* \ell _{j}(B^* E_m A^*) V_j {r}_{j}(E_k) - V_j^* \ell _{j}(B^* E_m) V_j {r}_{j}(A^* E_k) \\&\qquad - V_j^* \ell _{j}(E_m A^*) V_j {r}_{j}(E_k B^*) + V_j^* \ell _{j}(E_m) V_j {r}_{j}(A^* E_k B^*) \Big ] \end{aligned}$$

which coincides with (7.6) after interchanging *m* and *k*. $\square$

The following result expressing the unique solvability of the continuity equation is now an immediate consequence.

#### Corollary 7.4

For $\rho \in {{\mathfrak {P}}}_+$, the linear mapping $\mathscr {K}_\rho$ is a bijection on $\mathcal {A}_0$ that depends smoothly $(C^\infty )$ on $\rho$.

#### Proof

It follows from Lemma 7.3 that $\mathscr {K}_\rho$ maps $\mathcal {A}_0$ into itself. Since the restriction of a self-adjoint operator to its range is injective, the result follows. Smooth dependence on $\rho$ follows from the smoothness of $\theta$. $\square$

The following elementary variational characterization is of interest.

#### Proposition 7.5

Fix $\rho \in {{\mathfrak {P}}}_+$ and $\nu \in \mathcal {A}_0$. Among all vector fields $\mathbf {B}\in \mathcal {B}$ satisfying the continuity equation

7.7 $$\begin{aligned} \nu + {{\,\mathrm{div}\,}}({\widehat{\rho }}\# \mathbf {B}) = 0 \end{aligned}$$

there is a unique one that is a gradient. Moreover, among all vector fields $\mathbf {B}$ solving (7.7), this vector field is the unique minimizer of the “kinetic energy functional” $\mathscr {E}_\rho$ given by

$$\begin{aligned} \mathscr {E}_\rho (\mathbf {B}) = \sum _{j \in \mathcal {J}} \langle {{\widehat{\rho }}_j \# B_j, B_j}\rangle _{L^2(\tau _j)} \ . \end{aligned}$$

#### Proof

Existence of a gradient vector $\mathbf {B}$ field solving (7.7) follows from Corollary 7.4. To prove uniqueness, suppose that ${{\,\mathrm{div}\,}}({\widehat{\rho }}\# \nabla A) = - \nu = {{\,\mathrm{div}\,}}({\widehat{\rho }}\# \nabla {\widetilde{A}})$ for some $A, {\widetilde{A}} \in \mathcal {A}$. This means that $\mathscr {K}_\rho A = \mathscr {K}_\rho {\widetilde{A}}$, hence Lemma 7.3 yields $\nabla A = \nabla {\widetilde{A}}$. The remaining part follows along the lines of the proof of [8, Theorem 3.17]. $\square$

We are now ready to define a class of Riemannian metrics that are the main object of study in this paper.

#### Definition 7.6

(*Quantum transport metric*) Fix $\rho \in {{\mathfrak {P}}}_+$ and let $\theta = (\theta _j)_j$ satisfy Assumption 7.2. The associated *quantum transport metric* is the Riemannian metric on $\mathscr {M}_\rho$ induced by the operator $\mathscr {K}_\rho$, i.e., for ${{\dot{\rho }}}_1, {\dot{\rho }}_2 \in \mathcal {A}_0$,

$$\begin{aligned} \langle {{\dot{\rho }}_1, {\dot{\rho }}_2}\rangle _\rho = \langle {\mathscr {K}_\rho ^{-1} {\dot{\rho }}_1, {\dot{\rho }}_2}\rangle _{L^2(\tau )} \ , \end{aligned}$$

or, more explicitly,

7.8 $$\begin{aligned} \begin{aligned} \langle {{\dot{\rho }}_1, {\dot{\rho }}_2}\rangle&= \langle { \nabla A_1, {\widehat{\rho }}\# \nabla A_2 }\rangle _{L^2(\tau )}\\&= \sum _j \big \langle { \partial _j A_1, \theta _j\big (\ell _j(\rho ), {r}_j(\rho )\big ) \# \partial _j A_2 }\big \rangle _{L^2(\tau _j)} \quad \text { for } \rho \in {{\mathfrak {P}}}_+\ , \end{aligned} \end{aligned}$$

where, for $i = 1,2$, $A_i$ is the unique solution in $\mathcal {A}_0$ to the continuity equation

$$\begin{aligned} {\dot{\rho }}_i + {{\,\mathrm{div}\,}}({\widehat{\rho }}\# \nabla A_i) = 0 \ . \end{aligned}$$

It follows from Lemma 7.3 and Corollary 7.4 that $\mathscr {K}_\rho$ indeed induces a Riemannian metric on $\mathscr {M}_\rho$.

### Gradient Flows of Entropy Functionals

In this section we shall show that various evolution equations of interest can be interpreted as gradient flow equations with respect to suitable quantum transport metrics introduced in Sect. 7.1.

We consider the operator $\mathscr {K}_\rho : \mathcal {A}_0 \rightarrow \mathcal {A}_0$ given by

$$\begin{aligned} \mathscr {K}_\rho A := \sum _{j \in \mathcal {J}} \partial _j^\dagger ({\widehat{\rho }}_j \# \partial _j A) \ , \end{aligned}$$

where ${\widehat{\rho }}_j = \theta _j(\ell _j(\rho ), {r}_j(\rho ))$ is defined in terms of a well-chosen function $\theta _j$ that depends on the context and will be determined below.

#### Theorem 7.7

(Gradient flow structure for the relative entropy) Consider the operator $\mathscr {K}_\rho$ defined using the functions $\theta _j$ given by $\theta _j(r,s) := \Lambda (e^{\omega _j/2}r, e^{-\omega _j/2}s)$, where $\Lambda (r,s) = \frac{r-s}{\log r - \log s}$ is the logarithmic mean. Then we have the identity

$$\begin{aligned} \mathscr {L}^\dagger \rho = - \mathscr {K}_\rho \mathrm {D}{{\,\mathrm{Ent}\,}}_{\sigma }(\rho ) \end{aligned}$$

for all $\rho \in {{\mathfrak {P}}}_+$, thus the gradient flow equation for the relative von Neumann entropy functional ${{\,\mathrm{Ent}\,}}_\sigma$ with respect to the Riemannian metric on $\mathscr {M}_\sigma$ induced by $(\mathscr {K}_\rho )_{\rho \in \mathscr {M}_\sigma }$ is the Kolmogorov forward equation $\partial _t \rho = \mathscr {L}^\dagger \rho$.

This result generalises the gradient flow structure from [10, 36] as described in Sect. 2. The proof relies on the following version of the chain rule.

#### Lemma 7.8

(Chain rule for the logarithm) Define $\theta _j(r,s) := \Lambda (e^{\omega _j/2}r, e^{-\omega _j/2}s)$, where $\Lambda (r,s) = \frac{r-s}{\log r - \log s}$ is the logarithmic mean. Then, for all $\rho \in {{\mathfrak {P}}}_+$ we have

7.9 $$\begin{aligned} e^{-\omega _j/2}V_j {r}_j(\rho ) - e^{\omega _j/2}\ell _j(\rho ) V_j&= \widehat{\rho _{j}}\# \partial _{j}(\log \rho - \log \sigma ) \ . \end{aligned}$$

#### Proof

Using (4.18) we infer that

$$\begin{aligned} \partial _{j}( \log \rho - \log \sigma ) = V_j \log \big (e^{-\omega _j/2} {r}_j(\rho )\big ) - \log \big (e^{\omega _j/2} \ell _j(\rho )\big ) V_j \ . \end{aligned}$$

We consider the spectral decomposition $\rho = \sum _k \lambda _k E_k$ as before, and observe that

$$\begin{aligned} {\widehat{\rho }}_{j} = \theta _j\big (\ell _j(\rho ), {r}_j(\rho )\big ) = \sum _{k,m} \Lambda \big (e^{\omega _j/2} \lambda _k, e^{-\omega _j/2} \lambda _m\big ) \ell _j(E_k) \otimes {r}_j(E_m) \ . \end{aligned}$$

Using this identity, we obtain

$$\begin{aligned}&\widehat{\rho _{j}}\# \big ( \partial _{j}( \log \rho - \log \sigma ) \big ) \\&\quad = \sum _{k, m, p} \Lambda \big (e^{\omega _j/2} \lambda _k, e^{-\omega _j/2} \lambda _m\big ) \ell _j(E_k) \\&\qquad \times \Big ( \log (e^{-\omega _j/2} \lambda _p) V_j {r}_j(E_p) - \log (e^{\omega _j/2} \lambda _p) \ell _j(E_p) V_j \Big ) {r}_j(E_m)\\&\quad = \sum _{k, m} \Lambda (e^{\omega _j/2} \lambda _k, e^{-\omega _j/2} \lambda _m) \Big ( \log (e^{-\omega _j/2} \lambda _m) - \log (e^{\omega _j/2} \lambda _k) \Big ) \ell _j(E_k) V_j {r}_j(E_m)\\&\quad = \sum _{k, m} \big ( e^{-\omega _j/2} \lambda _m - e^{\omega _j/2} \lambda _k \big ) \ell _j(E_k) V_j {r}_j(E_m)\\&\quad = e^{-\omega _j/2} V_j {r}_j(\rho ) - e^{\omega _j/2} \ell _j(\rho ) V_j\ , \end{aligned}$$

which yields (7.9). $$\square $$

#### Proof of Theorem 7.7

Since $\mathrm {D}{{\,\mathrm{Ent}\,}}_{\sigma }(\rho ) = \log \rho - \log \sigma$, the chain rule from Lemma 7.8 yields, using Proposition 4.9,

$$\begin{aligned} \mathscr {K}_\rho \mathrm {D}{{\,\mathrm{Ent}\,}}_{\sigma }(\rho )&= \sum _{j \in \mathcal {J}} \partial _{j}^\dagger \big ( \widehat{\rho _{j}}\# \partial _{j} (\log \rho - \log \sigma ) \big ) \\&= \sum _{j \in \mathcal {J}} \partial _{j}^\dagger \big ( e^{-\omega _j/2}V_j {r}_j(\rho ) - e^{\omega _j/2}\ell _j(\rho ) V_j \big ) \\&= \sum _{j \in \mathcal {J}} e^{-\omega _j/2} \Big \{{r}_j^\dagger \big (V_j^* V_j {r}_j(\rho ) \big ) - \ell _j^\dagger \big ( V_j {r}_j(\rho ) V_j^*\big ) \Big \} \\&\quad - e^{\omega _j/2} \Big \{{r}_j^\dagger \big (V_j^* \ell _j(\rho ) V_j \big ) - \ell _j^\dagger \big ( \ell _j(\rho ) V_j V_j^*\big ) \Big \} \ , \end{aligned}$$

which equals the expression for $- \mathscr {L}^\dagger \rho$ given in Proposition 4.10. $\square$

Let us now consider the special case where $\sigma = {\mathbf{1}}$. Then (4.10) reduces to $\omega _j = 0$ for all $j \in \mathcal {J}$, and we will be able to formulate a natural nonlinear generalization of Theorem 7.7. Let $f \in C^2((0,\infty ); {{\mathbb {R}}})$ be strictly convex, and consider the functional $\mathcal {F}: {{\mathfrak {P}}}_+ \rightarrow {{\mathbb {R}}}$ given by

$$\begin{aligned} \mathcal {F}(\rho ) = \tau [f(\rho )]\ , \end{aligned}$$

where $f(\rho )$ is interpreted in the sense of functional calculus. Let $\varphi \in C^1((0,\infty );{{\mathbb {R}}})$ be strictly increasing, and consider the operator $\mathscr {K}_\rho$ as defined before, with $\theta _j = \theta$ given by

7.10 $$\begin{aligned} \theta (\lambda ,\mu ) = \left\{ \begin{array}{ll} \frac{\varphi (\lambda ) - \varphi (\mu )}{f'(\lambda ) - f'(\mu )}, &{} \text {if } \lambda \ne \mu ,\\ \frac{\varphi '(\lambda )}{f''(\lambda )}, &{} \text {otherwise}. \end{array} \right. \end{aligned}$$

The following result is a non-commutative analogue of a seminal result by Otto [38], which states that the porous medium equation is the gradient flow equation for the Rényi entropy in with respect to the 2-Kantorovich metric.

#### Theorem 7.9

(Gradient flow structures with general entropy functionals) Consider a differentiable structure with $\sigma = {\mathbf{1}}$, and let $\theta _j$ be given by (7.10). Then we have the identity

7.11 $$\begin{aligned} \mathscr {L}\varphi (\rho ) = \mathscr {L}^\dagger \varphi (\rho ) = - \mathscr {K}_\rho \mathrm {D}\mathcal {F}(\rho ) \end{aligned}$$

for $\rho \in {{\mathfrak {P}}}_+$, thus the gradient flow equation for $\mathcal {F}$ with respect to the Riemannian metric on $\mathscr {M}_{\mathbf{1}}$ induced by $(\mathscr {K}_\rho )_{\rho \in \mathscr {M}_{\mathbf{1}}}$ is given by

$$\begin{aligned} \partial _t\rho = \mathscr {L}\varphi (\rho ) \ . \end{aligned}$$

#### Proof

The first identity in (7.11) follows immediately from the construction of $\mathscr {L}$ since $\sigma = {\mathbf{1}}$. The chain rule (6.10) implies that the derivative of $\mathcal {F}$ is given by

$$\begin{aligned} \mathrm {D}\mathcal {F}(\rho ) = f'(\rho ) \ . \end{aligned}$$

Recalling (6.5), we note that $\theta _j$ is defined to satisfy the identity $\theta \cdot \delta f' = \delta \varphi$. Using (6.4), (7.10), and the chain rule from Proposition 6.2 we infer that

$$\begin{aligned} {\widehat{\rho }}_j \# \partial _j \mathrm {D}\mathcal {F}(\rho )&= \theta \big (\ell _j(\rho ), {r}_j(\rho )\big ) \#\Big ( \delta f'\big (\ell _j(\rho ), {r}_j(\rho )\big ) \# \partial _j\rho \Big ) \\&= \delta \varphi \big (\ell _j(\rho ), {r}_j(\rho )\big ) \# \partial _j\rho \\&= \partial _j\varphi (\rho ) \ . \end{aligned}$$

We obtain

$$\begin{aligned} \mathscr {K}_\rho \mathrm {D}\mathcal {F}(\rho )&= \sum _{j \in \mathcal {J}} \partial _{j}^\dagger \big ( \widehat{\rho _{j}}\# \partial _{j} \mathrm {D}\mathcal {F}(\rho ) \big ) = \sum _{j \in \mathcal {J}} \partial _{j}^\dagger \partial _j\varphi (\rho ) = - \mathscr {L}\varphi (\rho )\; , \end{aligned}$$

which is the desired identity. $\square$

#### Remark 7.10

The result remains true if *f* is required to be strictly concave and $\varphi$ is required to be strictly decreasing. Note that $\theta$ is positive in this case, so that $(\mathscr {K}_\rho )_\rho$ induces a Riemannian metric.

#### Remark 7.11

This result contains various known results as special cases. Take $f(\lambda ) = \lambda \log \lambda$ and $\varphi (r) = r$. Then the functional $\mathcal {F}$ is the von Neumann entropy $\mathcal {F}(\rho ) = \tau [\rho \log \rho ]$, and we recover the special case of Theorem 7.7 with $\sigma = {\mathbf{1}}$. It also contains the gradient flow structure for the fermionic Fokker-Planck equation from [8]. In the special case where $\mathscr {L}$ is the generator of a reversible Markov chain, we recover the gradient flow structure for discrete porous medium equations obtained in [19].

#### Remark 7.12

In some situations the expression for ${\widehat{\rho }}_j = \theta (\ell _j(\rho ), {r}_j(\rho ))$ can be simplified. If $f(\lambda ) = \lambda \log \lambda$ and $\varphi (\lambda ) = \lambda$, it follows that $\theta (\lambda ,\mu ) = \frac{\lambda - \mu }{\log \lambda - \log \mu }$ is the *logarithmic mean*. The integral representation $\theta (\lambda ,\mu ) = \int _0^1 \lambda ^{1-s}\mu ^s \; \mathrm {d}s$ allows one to express ${\widehat{\rho }}_j$ in terms of the functional calculus for $\ell _j(\rho )$ and ${r}_j(\rho )$:

$$\begin{aligned} {\widehat{\rho }}_j = \theta (\ell _j(\rho ), {r}_j(\rho )) = \int _0^1 \ell _j(\rho )^{1-s} \otimes {r}_j(\rho )^s \; \mathrm {d}s\ . \end{aligned}$$

More generally, take $m \in {{\mathbb {R}}}{\setminus } \{0,1\}$, and set $\varphi (\lambda ) = \lambda ^m$ and $f(\lambda ) = \frac{1}{m-1}\lambda ^m$. We shall consider the *power difference means* defined by

$$\begin{aligned} \theta _m(\lambda ,\mu ) := \frac{m-1}{m}\frac{\lambda ^m - \mu ^m}{\lambda ^{m-1} - \mu ^{m-1}}\ , \end{aligned}$$

with the convention that $\theta _m(\lambda , \lambda ) = \lambda$. A systematic study of the operator means associated to these functions has been carried out in [25]. Various classical means are contained as special cases:

$$\begin{aligned} \displaystyle \theta _{m}(\lambda ,\mu )= \left\{ \begin{array}{ll} \displaystyle \frac{2\lambda \mu }{\lambda +\mu }\ , &{} m = -1\text { (harmonic mean)}\ ,\\ \displaystyle \frac{\lambda \mu (\log \lambda - \log \mu )}{\lambda - \mu }\ , &{} m \rightarrow 0\ ,\\ \displaystyle \sqrt{\lambda \mu }\ , &{} m = \frac{1}{2} \text { (geometric mean)}\ ,\\ \displaystyle \frac{\lambda - \mu }{\log \lambda - \log \mu }\ ,&{} m \rightarrow 1 \text { (logarithmic mean)}\ ,\\ \displaystyle \frac{\lambda +\mu }{2}\ ,&{} m = 2 \text { (arithmetic mean)}\ .\\ \end{array} \right. \end{aligned}$$

The following integral representation holds:

7.12 $$\begin{aligned} \theta _m(\lambda ,\mu )= \int _0^1 \Big ( (1-\alpha ) \lambda ^{m-1} + \alpha \mu ^{m-1} \Big )^{\frac{1}{m-1}} \; \mathrm {d}\alpha \ . \end{aligned}$$

If $m = 2$ and $\ell _j = {r}_j = I_\mathcal {A}$, one has ${\widehat{\rho }}_j \# A = \frac{1}{2}(\rho A + A \rho )$, which corresponds to the anti-commutator case studied in [12].

Another special case is obtained by taking $\varphi (\lambda ) = \lambda$ and $f(\lambda ) = \lambda ^2/2$, which yields $\theta (\lambda , \mu ) \equiv 1$, so that $\mathscr {K}_\rho = -\mathscr {L}$ for all $\rho$, and $\mathcal {F}(\rho ) = \frac{1}{2}\tau [\rho ^2] = \frac{1}{2} \Vert \rho \Vert _{L^2(\tau )}^2$. In this case, the distance associated to $\mathscr {K}_\rho$ may be regarded as a non-commutative analogue of the Sobolev $H^{-1}$-metric.

### Geodesics

As before we consider the operator $\mathscr {K}_\rho : \mathcal {A}_0 \rightarrow \mathcal {A}_0$ given by

$$\begin{aligned} \mathscr {K}_\rho A := \partial _j^\dagger ({\widehat{\rho }}_j \# \partial _j A) \ . \end{aligned}$$

For fixed ${\bar{\rho }} \in {{\mathfrak {P}}}_+$ we will compute the geodesic equations associated to the Riemannian structure on $\mathscr {M}_{{\bar{\rho }}}$ induced by the operator $(\mathscr {K}_\rho )_\rho$. The Riemannian distance $d_\mathscr {K}$ is given by

$$\begin{aligned} d_\mathscr {K}({\widetilde{\rho }}_0, {\widetilde{\rho }}_1)^2&= \inf _{\rho , A} \bigg \{ \int _0^1 \langle {\mathscr {K}_{\rho _t} A_t, A_t}\rangle _{L^2(\mathcal {A},\tau )} \; \mathrm {d}t \ : \ \partial _t \rho _t = \mathscr {K}_{\rho _t} A_t\ , \rho _0 = {\widetilde{\rho }}_0\ , \rho _1 = {\widetilde{\rho }}_1 \bigg \} \\&= \inf _{\rho , A} \bigg \{ \int _0^1 \langle {{\widehat{\rho }}_t \# \nabla A_t, \nabla A_t}\rangle _{L^2(\mathcal {A},\tau )} \; \mathrm {d}t \,:\,\\&\qquad \partial _t \rho _t + {{\,\mathrm{div}\,}}({\widehat{\rho }}_t\# \nabla A_t)= 0\,, \rho _0 = {\widetilde{\rho }}_0\ , \rho _1 = {\widetilde{\rho }}_1 \bigg \}\,, \end{aligned}$$

where the infimum runs over smooth curves $\{\rho _t\}_{t \in [0,1]}$ in $\mathscr {M}_{{\bar{\rho }}}$ and $\{A_t\}_{t \in [0,1]}$ in $\mathcal {A}_0$ satisfying the stated conditions.

The geodesic equations are the Euler–Lagrange equations associated to this constrained minimization problem, given by

7.13 $$\begin{aligned} \begin{aligned} \partial _s \rho _s&= \mathscr {K}_{\rho _s} A_s \\ \partial _s A_s&= - \frac{1}{2} \langle {\mathrm {D}\mathscr {K}_{\rho _s} A_s, A_s}\rangle _{L^2(\mathcal {A}, \tau )} \ . \end{aligned} \end{aligned}$$

Note that the latter equation is equivalent to

$$\begin{aligned} \partial _s \tau [A_s B]&= - \frac{1}{2} \langle {\mathrm {D}_B \mathscr {K}_{\rho _s} A_s, A_s}\rangle _{L^2(\mathcal {A}, \tau )} \end{aligned}$$

for $B \in \mathcal {A}_0$, where $\mathrm {D}_B \mathscr {K}_{\rho } = \lim _{\varepsilon \rightarrow 0} \varepsilon ^{-1}\big (\mathscr {K}_{\rho + \varepsilon B} - \mathscr {K}_{\rho }\big )$ denotes the directional derivative.

#### Proposition 7.13

(Geodesic equations) The geodesic equations for $(\rho _s, A_s)_s$ are given by

7.14 $$\begin{aligned} \partial _s \rho _s + {{\,\mathrm{div}\,}}({\widehat{\rho }}_s \# \nabla A_s)&= 0\ , \end{aligned}$$

7.15 $$\begin{aligned} \partial _s A_s + \Phi (\rho _s, A_s)&= 0 \ , \end{aligned}$$

whereHere, $\rho = \sum _k \lambda _k E_k$ denotes the spectral decomposition of $\rho$.

#### Remark 7.14

In the sequel we will use (7.15) in the weak formulation:

7.16 $$\begin{aligned} \begin{aligned} \partial _s \tau [A_sB]&= - \sum _{j \in \mathcal {J}} \tau _j\Big [(\partial _j A_s)^* \mathcal {N}_{\rho ,B}^{(\eta ),j} \# (\partial _j A_s) \Big ] \ , \end{aligned} \end{aligned}$$

for all $B \in \mathcal {A}$ and $\eta = 1,2$, where

#### Remark 7.15

If $\theta _j(r,s) := \Lambda (e^{\omega _j/2}r, e^{-\omega _j/2}s)$ where $\Lambda$ is the logarithmic mean, the expression above can be simplified. In this case we have the integral representation


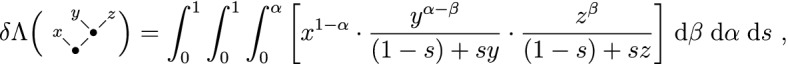


so that


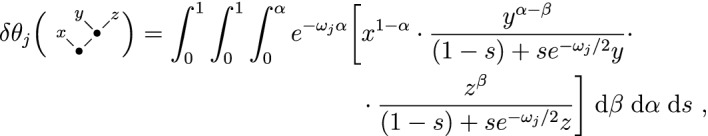


which implies that

$$\begin{aligned} \Phi (\rho , A)&= \sum _{j \in \mathcal {J}}\int _0^1 \int _0^1 \int _0^\alpha e^{\omega _j \alpha } \bigg [ \frac{\rho ^{\alpha - \beta }}{(1-s)I + s e^{\omega _j/2}\rho } \ell _j^\dagger \Big ( (\partial _j A) {r}_j(\rho ^{1-\alpha }) (\partial _j A)^* \Big ) \\&\quad \times \frac{\rho ^\beta }{(1-s)I + s e^{\omega _j/2}\rho } \bigg ] \; \mathrm {d}\beta \; \mathrm {d}\alpha \; \mathrm {d}s \\&= \sum _{j \in \mathcal {J}}\int _0^1 \int _0^1 \int _0^\alpha e^{-\omega _j\alpha } \bigg [ \frac{\rho ^{\alpha - \beta }}{(1-s)I + s e^{-\omega _j/2} \rho } {r}_j^\dagger \Big ( (\partial _j A)^* \ell _j(\rho ^{1-\alpha }) \partial _j A \Big ) \\&\quad \times \frac{\rho ^\beta }{(1-s)I + s e^{-\omega _j/2} \rho } \bigg ] \; \mathrm {d}\beta \; \mathrm {d}\alpha \; \mathrm {d}s \ . \end{aligned}$$

#### Proof of Proposition 7.13

Proposition 6.8 yieldsand thereforeSince *A* is self-adjoint, it follows using (7.1) and (4.17) thatThis implies the equality of the two sums in (7.16), and it also follows that7.17which yields the weak formulation (7.16) in view of (7.13). To obtain (7.15), we compute using (4.4),whereAn analogous computation shows thatWe thus obtain

$$\begin{aligned} \langle {\mathrm {D}\mathscr {K}_{\rho } A, A}\rangle _{L^2(\mathcal {A}, \tau )} = 2 \Phi (\rho , A) \ , \end{aligned}$$

hence the result follows from the Euler–Lagrange equations (7.13). $\square$

We will use the geodesic equations to compute the Hessian of some interesting functionals on $\mathscr {M}_\rho$. Note that the Hessian is obtained from the formula

$$\begin{aligned} {{\,\mathrm{Hess}\,}}_\mathscr {K}\mathscr {E}(\rho _0)[A_0, A_0] := \partial _s^2\big |_{s=0} \mathscr {E}(\rho _s) \end{aligned}$$

for $A_0 \in \mathcal {A}_0$, where $(\rho _s, A_s)_s$ evolves according to the geodesic equations (7.13) with initial conditions $\rho \big |_{s = 0} = \rho _0$ and $\partial _s \big |_{s = 0} \rho _s = \mathscr {K}_{\rho _0} A_0$.

#### Proposition 7.16

For ${\bar{\rho }} \in {{\mathfrak {P}}}_+$, let $\mathscr {E}: \mathscr {M}_{{\bar{\rho }}} \rightarrow {{\mathbb {R}}}$ be a smooth functional, and let us write $\mathscr {M}(\rho ) := \mathscr {K}_\rho \mathrm {D}\mathcal {F}(\rho )$ for the Riemannian gradient of $\mathcal {F}$ induced by $(\mathscr {K}_\rho )_\rho$. Then, the Hessian of $\mathscr {E}$ is given by

7.18 $$\begin{aligned} {{\,\mathrm{Hess}\,}}_\mathscr {K}\mathscr {E}(\rho )[A, A] = \tau [ A \mathrm {D}_{\mathscr {K}_{\rho } A} \mathscr {M}(\rho ) ] - \tau \Big [(\nabla A)^* \mathcal {N}_{\rho ,\mathscr {M}(\rho )}^{(\eta )} \# (\nabla A) \Big ] \end{aligned}$$

for $A \in \mathcal {A}_0$ and $\eta = 1, 2$, where $\mathrm {D}_{B} \mathscr {M}(\rho ) = \lim _{\varepsilon \rightarrow 0} \varepsilon ^{-1}\big (\mathscr {M}(\rho + \varepsilon B) - \mathscr {M}(\rho )\big )$ denotes the directional derivative. In particular, if $\mathscr {M}(\rho ) = -\mathscr {L}^\dagger \rho$ (as is the case in setting of Theorem 7.7, where $\mathscr {E}(\rho ) = {{\,\mathrm{Ent}\,}}_\sigma (\rho )$), we have

7.19 $$\begin{aligned} {{\,\mathrm{Hess}\,}}_\mathscr {K}\mathscr {E}(\rho )[A, A] = - \tau [( \nabla \mathscr {L}A)^* {\widehat{\rho }}\# \nabla A] + \tau \Big [(\nabla A)^* \mathcal {N}_{\rho ,\mathscr {L}^\dagger \rho }^{(\eta )} \# (\nabla A) \Big ] \ . \end{aligned}$$

#### Proof

Let $(\rho _s,A_s)_s$ satisfy the geodesic equations (7.14)–(7.15). Then:

$$\begin{aligned} \partial _s \mathscr {E}(\rho _s)&= \tau [ \mathrm {D}\mathscr {E}(\rho _s) \partial _s \rho _s ] = \tau [ \mathrm {D}\mathscr {E}(\rho _s) \mathscr {K}_{\rho _s} A_s ] = \tau [ A_s \mathscr {K}_{\rho _s} \mathrm {D}\mathscr {E}(\rho _s) ] = \tau [ A_s \mathscr {M}(\rho _s) ] \ . \end{aligned}$$

Thus, by (7.16),

$$\begin{aligned} \partial _s^2 \mathscr {E}(\rho _s)&= \tau [ A_s \partial _s \mathscr {M}(\rho _s) ] +\tau [ (\partial _s A_s) \mathscr {M}(\rho _s) ] \\&= \tau [ A_s \mathrm {D}_{\mathscr {K}_{\rho _s} A_s} \mathscr {M}(\rho _s) ] - \sum _{j \in \mathcal {J}} \tau _j\Big [(\partial _j A_s)^* \mathcal {N}_{\rho _s,\mathscr {M}(\rho _s)}^{(\eta ),j} \# (\partial _j A_s) \Big ] \ , \end{aligned}$$

for $\eta = 1, 2$, which proves (7.18).

If $\mathscr {M}(\rho ) = -\mathscr {L}^\dagger \rho$ we have $\mathrm {D}_{B} \mathscr {M}(\rho ) = - \mathscr {L}^\dagger B$, hence the expression above simplifies to

$$\begin{aligned} \partial _s^2 \mathscr {E}(\rho _s)&= - \tau [ A_s \mathscr {L}^\dagger \mathscr {K}_{\rho _s} A_s ] + \sum _{j \in \mathcal {J}} \tau _j\Big [(\partial _j A_s)^* \mathcal {N}_{\rho _s,\mathscr {L}^\dagger \rho _s}^{(\eta ),j} \# (\partial _j A_s) \Big ]\\&= - \tau [( \nabla \mathscr {L}A_s)^* {\widehat{\rho }}_s \# \nabla A_s] + \sum _{j \in \mathcal {J}} \tau _j\Big [(\partial _j A_s)^* \mathcal {N}_{\rho _s,\mathscr {L}^\dagger \rho _s}^{(\eta ),j} \# (\partial _j A_s) \Big ] \ . \end{aligned}$$

$$\square $$

#### Remark 7.17

In the setting of the theorem above, we remark that the following equivalent expression holds as well:

$$\begin{aligned} {{\,\mathrm{Hess}\,}}_\mathscr {K}\mathscr {E}(\rho )[A, A] = \tau [ A \mathrm {D}_{ \mathscr {K}_{\rho } A} \mathscr {M}(\rho ) ] - \tau [ \Phi (\rho , A) \mathscr {M}(\rho ) ] \ . \end{aligned}$$

## Preliminaries on Quasi-entropies

In this section we collect some known results on trace functionals that will be useful in the study of quantum transport metrics. Special cases of the results in this section already played a key role in the proof of functional inequalities in [10].

Let $\mathcal {A}$ be a finite-dimensional von Neumann algebra endowed with a positive tracial linear functional $\tau$. We consider the mapping $\mathcal {J}_{\theta ,p} : \mathcal {A}_+ \times \mathcal {A}_+ \times \mathcal {A}\rightarrow {{\mathbb {R}}}$ given by

$$\begin{aligned} \qquad \mathcal {J}_{\theta ,p}(R,S;A) := \big \langle {A, \theta ^{-p}(R,S) \# A }\big \rangle = \sum _{k,\ell } \theta ^{-p}(\lambda _k, \mu _\ell ) \tau \big [A^* E_{k}^R A E_{\ell }^S \big ]\ , \end{aligned}$$

where $\theta : (0,\infty ) \times (0,\infty ) \rightarrow (0,\infty )$ and $p \in {{\mathbb {R}}}$, and $R = \sum _k \lambda _k E_k^R$ and $S = \sum _\ell \mu _\ell E_\ell ^S$ denotes the spectral decomposition. The main cases of interest to us are $p = \pm 1$.

In this section we shall assume that the function $\theta$ is 1*-homogeneous*, i.e., $\theta (\lambda r,\lambda s) = \lambda \theta (r,s)$ for all $\lambda , r, s > 0$. Clearly, this assumption is satisfied if and only if there exists a function $f : (0,\infty ) \rightarrow (0,\infty )$ such that $\theta (r,s) = s f(r/s)$ for all $r,s > 0$, in which case we have $f(r) = \theta (r,1)$. To simplify notation, we write $k(r) = 1/f(r)$.

### Remark 8.1

(Relation to the relative modular operator) It is instructive to see how the definition of $\theta (R,S)$ can be formulated in terms of the relative modular operator, if $\theta$ is 1-homogeneous. Given $S \in \mathcal {A}_+$, let ${\mathsf {L}}_S$ and ${\mathsf {R}}_S$ denote the left- and right-multiplication operators defined by $\mathsf {L}_S(A) = S A$ and $\mathsf {R}_S(A) = A S$. Then the relative modular operator $\Delta _{R,S} : \mathcal {A}\rightarrow \mathcal {A}$ defined by $\Delta _{R,S} A = RAS^{-1}$ can be expressed as $\Delta _{R,S} = \mathsf {L}_R \circ \mathsf {R}_{S^{-1}} = \mathsf {R}_{S^{-1}} \circ \mathsf {L}_R$. Let $\{\xi _k\}$ (resp. $\{\eta _\ell \}$) be an orthonormal basis of ${{\mathbb {C}}}^n$ consisting of eigenvectors of *R* (resp. *S*), let $\{\lambda _k\}$ (resp. $\{\mu _\ell \}$) be the corresponding eigenvalues, and set $E_{k\ell } := \,|\xi _k\rangle \langle \eta _\ell |\,$. It follows that $\Delta _{R,S}(E_{k \ell }) = \frac{\lambda _k}{\mu _\ell } E_{k \ell }$, hence the $E_{k\ell }$’s form a complete basis of eigenvectors of $\Delta _{R,S}$. Moreover, the $E_{k\ell }$’s are orthonormal with respect to the Hilbert–Schmidt inner product $\langle {A,B}\rangle _{L^2({{\,\mathrm{Tr}\,}})} = {{\,\mathrm{Tr}\,}}[A^* B]$ on ${{\mathbb {M}}}_n({{\mathbb {C}}})$. Consequently, the spectral decomposition of $\Delta _{R,S}$ is given by

$$\begin{aligned} \Delta _{R,S} = \sum _{k, \ell } \frac{\lambda _k}{\mu _\ell } \,|E_{k\ell }\rangle \langle E_{k\ell }|\, \ , \end{aligned}$$

and for functions $f : (0,\infty ) \rightarrow {{\mathbb {R}}}$ we find $f(\Delta _{R,S})(A) = \sum _{k, \ell }f (\lambda _k / \mu _\ell ) \langle {E_{k\ell }, A}\rangle _{L^2({{\,\mathrm{Tr}\,}})} E_{k\ell }$. Note that

$$\begin{aligned} \langle {E_{k\ell }, A}\rangle _{L^2({{\,\mathrm{Tr}\,}})} E_{k \ell } = \sum _m \langle {E_{k\ell }\eta _m, A\eta _m }\rangle E_{k \ell } = \langle {\xi _k, A\eta _\ell }\rangle E_{k \ell } = E_{k}^R A E_{\ell }^S \ , \end{aligned}$$

where $E_{k}^R = \,|\xi _k\rangle \langle \xi _k|\,$ and $E_{\ell }^S = \,|\eta _\ell \rangle \langle \eta _\ell |\,$. It follows that

$$\begin{aligned} f(\Delta _{R,S})(A) = \sum _{k, \ell } f(\lambda _k / \mu _\ell ) E_{k}^R A E_{\ell }^S \ , \end{aligned}$$

and therefore, since $f(r/s) s = \theta (r,s)$,

$$\begin{aligned} \theta (R,S) \# A = \big (\mathsf {R}_S \circ f(\Delta _{R,S}) \big )(A) \ . \end{aligned}$$

### Example 8.2

Let us recall our main examples of interest. A central role is played by the *tilted logarithmic mean* $\theta _{1,\beta }$ given by

$$\begin{aligned} \theta _{1,\beta }(r,s)&= \int _0^1 (e^{-\beta /2}r)^{1-\alpha } (e^{\beta /2}s)^\alpha \; \mathrm {d}\alpha = \frac{e^{-\beta /2}r - e^{\beta /2}s}{- \beta + \log r - \log s} \ , \quad \\ f_{1,\beta }(r)&= \frac{e^{-\beta /2} r - e^{\beta /2}}{-\beta + \log r} \ , \end{aligned}$$

for $\beta \in {{\mathbb {R}}}$. More generally, in view of Remark 7.12 we are interested in the class of power difference quotients $\theta _m$ given by $f_{m,\beta }(r) = \theta _{m,\beta }(r,1)$, where

$$\begin{aligned} \theta _{m,\beta }(r,s)&= \int _0^1 \Big ( (1-\alpha )(e^{-\beta /2}r)^{m-1} + \alpha (e^{\beta /2}s)^{m-1} \Big )^{\frac{1}{m-1}} \; \mathrm {d}\alpha \\&= \frac{m-1}{m}\frac{(e^{-\beta /2}r)^m - (e^{\beta /2}s)^m}{(e^{-\beta /2}r)^{m-1} - (e^{\beta /2}s)^{m-1}}. \end{aligned}$$

Consider the mapping $\Upsilon _{f,p} : \mathcal {A}_+\times \mathcal {A}_+ \times \mathcal {A}\rightarrow {{\mathbb {R}}}$ given by

$$\begin{aligned} \Upsilon _{f,p}(R,S;A) := \mathcal {J}_{\theta ,p}(R,S;A) = \big \langle {A, \theta ^{-p}(R,S) \# A }\big \rangle \end{aligned}$$

Our goal is to characterize its convexity and contractivity properties in terms of *f* and *m*. For this purpose we recall that a function $f : (0,\infty ) \rightarrow (0,\infty )$ is said to be *operator monotone*, whenever $f(A) \le f(B)$ for all positive matrices $A \le B$ in all dimensions. Each operator monotone function is continuous, non-decreasing and concave. We set $f(0) := \inf _{t > 0} f(t)$.

The following result has been obtained in [27, Theorem 2.1]. The implication “$(2) \Rightarrow (1)$”, as well as the reverse implication for fixed $p =1$ had already been proved in [26].

### Theorem 8.3

(Characterization of convexity of $\Upsilon _{f,p}$) Let $f : (0,\infty ) \rightarrow (0,\infty )$ be a function and let $p \in {{\mathbb {R}}}{\setminus } \{0 \}$. The following assertions are equivalent.

1. The function $\Upsilon _{f,p}$ is jointly convex in its three variables;
2. The function *f* is operator monotone and $p \in (0,1]$.

Applying this result to the functions $f = f_{m,\beta }$, we obtain the following result.

### Corollary 8.4

(Characterization of convexity of $\Upsilon _{f,p}$ for power difference quotients) For $m \in {{\mathbb {R}}}{\setminus } \{0\}$ and $\beta \in {{\mathbb {R}}}$, let $f = f_{m,\beta }$ and $\theta = \theta _{m,\beta }$ be as in Example 8.2. Then, the associated mapping $\Upsilon _{f,p}$ is jointly convex if and only if $m \in [-1,2]$, $p \in (0,1]$, and $\beta \in {{\mathbb {R}}}$. In particular, the mapping

$$\begin{aligned} (R,S,A) \mapsto \big \langle {A^*, \theta _{1,\beta }^{-1}(R,S) \# A }\big \rangle = \tau \bigg [\int _0^\infty A^* \frac{1}{x + e^{-\beta /2}R} A \frac{1}{x + e^{\beta /2}S} \; \mathrm {d}x \bigg ] \end{aligned}$$

is jointly convex for all $\beta \in {{\mathbb {R}}}$.

### Proof

Since $f_{m,\beta }(s) = e^{\beta /2} f_{m,0}(e^{-\beta }s)$, the operator monotonicity of $f_{m,\beta }$ does not depend on $\beta$. It has been proved in [25, Proposition 4.2], that $f_{m,0}$ is operator monotone if and only if $m \in [-1,2]$. Hence, the first assertion follows from Theorem 8.3. The second assertion is the special case $m = p = 1$, noting that

$$\begin{aligned} \frac{1}{\theta _{1,\beta }(r,s)} = \int _0^\infty \frac{1}{x + e^{-\beta /2}r} \, \frac{1}{x + e^{\beta /2}s} \; \mathrm {d}x \ . \end{aligned}$$

$$\square $$

### Remark 8.5

In the case where $\theta = \theta _{1,\beta }$, the operator monotonicity of $f_{1,\beta }$ can be checked elementarily, by writing $f_{1,\beta }(r) = \int _0^1 e^{-\beta (1/2-\alpha )} r^\alpha \; \mathrm {d}\alpha$, and applying the Löwner-Heinz Theorem (e.g., [7, Theorem 2.6]), which asserts that the function $r \mapsto r^\alpha$ is operator monotone for $\alpha \in [0,1]$.

The following result is proved in [26, Theorem 5].

### Theorem 8.6

(Contractivity of $\Theta _{f,p}$ under CPTC maps) Suppose that $f : (0,\infty ) \rightarrow (0,\infty )$ is operator monotone. Then, for any $R, S\in \mathcal {A}_+$ and $A \in \mathcal {A}$, and for any completely positive and trace preserving map $\mathcal {T}: \mathcal {A}\rightarrow \mathcal {A}$, we have

8.1 $$\begin{aligned} \Upsilon _{f,1}\big (\mathcal {T}(R),\mathcal {T}(S);\mathcal {T}(A)\big ) \le \Upsilon _{f,1}(R,S;A) \ . \end{aligned}$$

In the case where $f = f_{m, \beta }$ as in Example 8.2, we obtain the following result.

### Corollary 8.7

(Contractivity of $\Theta _{f,p}$ for power difference quotients) Let $m \in [-1,2]$ and $\beta \in {{\mathbb {R}}}$, and let $f = f_{m,\beta }$ and $\theta = \theta _{m,\beta }$ be as in Example 8.2. Then, for any $R, S\in \mathcal {A}_+$ and $A \in \mathcal {A}$, and for any completely positive and trace preserving map $\mathscr {P}: \mathcal {A}\rightarrow \mathcal {A}$, (8.1) holds. In particular, for $m = 1$ we obtain

$$\begin{aligned}&\tau \bigg [\int _0^\infty \mathcal {T}(A)^* \frac{1}{x + e^{-\beta /2}\mathcal {T}(R)} \mathcal {T}(A) \frac{1}{x + e^{\beta /2}\mathcal {T}(S)} \; \mathrm {d}x \bigg ] \\&\quad \le \tau \bigg [\int _0^\infty A^* \frac{1}{x + e^{-\beta /2}R} A \frac{1}{x + e^{\beta /2}S} \; \mathrm {d}x \bigg ] \ . \end{aligned}$$

### Proof

This follows from Theorem 8.6, as the operator monotonicity of $f_{m,p}$ had already been noted in Corollary 8.4. $\square$

## The Riemannian Distance

Fix a differentiable structure $(\mathcal {A}, \nabla , \sigma )$ in the sense of Definition 4.7 and a collection of functions $(\theta _j)_j$ satisfying Assumption 7.2. For simplicity we restrict ourselves to the ergodic case, so that $\mathscr {M}_\rho = {{\mathfrak {P}}}_+$ for all $\rho \in {{\mathfrak {P}}}_+$.

In this section we study basic properties of the Riemannian distance $\mathscr {W}$ associated to the operators $(\mathscr {K}_\rho )_\rho$ defined in (7.4). For $\rho _0, \rho _1 \in {{\mathfrak {P}}}_+$ this distance is given by

9.1 $$\begin{aligned} \begin{aligned} \mathscr {W}(\rho _0, \rho _1)^2&= \inf _{} \bigg \{ \int _0^1 \langle {\mathscr {K}_{\rho _t} A_t, A_t}\rangle \; \mathrm {d}t \ : \ \partial _t \rho _t = \mathscr {K}_{\rho _t}A_t\ , \rho _t|_{t=0} = \rho _0\ , \rho _t|_{t=1} = \rho _1 \bigg \} \\&= \inf _{} \bigg \{ \int _0^1 \tau \big [(\nabla A_t)^* {\widehat{\rho }}_t \# \nabla A_t \big ] \; \mathrm {d}t \ : \ \\&\qquad \partial _t \rho _t + {{\,\mathrm{div}\,}}({\widehat{\rho }}_t\# \nabla A_t)= 0\ , \ \rho _t|_{t=0,1} = \rho _{0,1} \bigg \}\ , \end{aligned} \end{aligned}$$

where the infimum runs over smooth curves $(\rho _t)_{t \in [0,1]}$ in ${{\mathfrak {P}}}_+$ and $(A_t)_{t \in [0,1]}$ in $\mathcal {A}_0$ satisfying the stated conditions.

In the classical theory of optimal transport, it is a useful fact that the following equivalent formulations hold for the 2-Kantorovich distance on ${{\mathbb {R}}}^n$:

9.2 $$\begin{aligned} W_2(\rho _0, \rho _1)^2&= \inf _{} \bigg \{ \int _0^1 |\nabla \psi _t(x)|^2 \; \mathrm {d}\rho _t(x) \; \mathrm {d}t \ : \ \partial _t \rho _t + {{\,\mathrm{div}\,}}(\rho _t \nabla \psi _t) = 0 \ , \rho _t|_{t=0,1} = \rho _{0,1} \bigg \} \nonumber \\&= \inf _{} \bigg \{ \int _0^1 \frac{|P_t(x)|^2}{\rho _t(x)} \; \mathrm {d}x \; \mathrm {d}t \ : \ \partial _t \rho _t + {{\,\mathrm{div}\,}}P_t = 0 \ , \rho _t|_{t=0,1} = \rho _{0,1} \bigg \} \ . \end{aligned}$$

The latter formulation has the advantage that the minimisation problem is convex, due to the convexity of the function $(p,r) \mapsto \frac{|p|^2}{r}$ on ${{\mathbb {R}}}^n \times (0,\infty )$.

Using the convexity results presented in Sect. 8 we will show that an analogous result holds in the non-commutative setting. We use the shorthand notation

$$\begin{aligned} \langle {\mathbf {B}, \mathbf {C}}\rangle _\rho = \sum _j \tau _j[B_j^* ({\widehat{\rho }}_j \# C_j) ] \ , \qquad \langle {\mathbf {B}, \mathbf {C}}\rangle _{-1,\rho } = \sum _j \tau _j[B_j^* ({\check{\rho }}_j \# C_j)] \ , \end{aligned}$$

to denote the scalar products that will frequently appear below. The corresponding norms are given by $\Vert \mathbf {B}\Vert _\rho = \sqrt{ \langle {\mathbf {B}, \mathbf {B}}\rangle _\rho }$ and $\Vert \mathbf {B}\Vert _{-1, \rho } = \sqrt{ \langle {\mathbf {B}, \mathbf {B}}\rangle _{-1,\rho }}$. It will occasionally be convenient to write

$$\begin{aligned} \mathscr {A}(\rho ; \mathbf {B}, \mathbf {C}) = \langle {\mathbf {B}, \mathbf {C}}\rangle _{-1,\rho } \quad \text {and} \quad \mathscr {A}(\rho , \mathbf {B}) = \Vert \mathbf {B}\Vert _{-1, \rho }^2 \ . \end{aligned}$$

We start with a non-commutative analogue of (9.2).

### Lemma 9.1

For $\rho _0, \rho _1 \in {{\mathfrak {P}}}_+$ we have

9.3 $$\begin{aligned} \mathscr {W}(\rho _0, \rho _1)^2&= \inf _{} \bigg \{ \int _0^1 \Vert \mathbf {B}_t \Vert _{-1, \rho _t}^2 \; \mathrm {d}t \ : \ \partial _t \rho _t + {{\,\mathrm{div}\,}}\mathbf {B}_t = 0\ , \ \rho _t|_{t=0,1} = \rho _{0,1} \bigg \} \ , \end{aligned}$$

where the infimum runs over all smooth curves $(\rho _t)_{t \in [0,1]}$ in ${{\mathfrak {P}}}_+$ and $(\mathbf {B}_t)_{t \in [0,1]}$ in $\mathcal {B}$.

### Proof

Any admissible curve $(A_t)$ in (9.1) yields an admissible curve $(\mathbf {B}_t)$ in (9.3) given by $\mathbf {B}_t = {\widehat{\rho }}_t \nabla A_t$, that satisfies $\Vert \nabla A_t\Vert _{\rho _t} = \Vert \mathbf {B}_t \Vert _{-1, \rho _t}$. This implies the inequality “$\ge$” in (9.3).

To prove the reverse inequality, we take an admissible curve $(\rho _t, \mathbf {B}_t)_t$ in (9.3). We consider the linear space of gradient vector fields $\mathscr {G}= \{\nabla A \ : A \in \mathcal {A}_0 \}$, and let $\mathcal {D}_t \subseteq \mathscr {G}$ denote its orthogonal complement in $\mathcal {B}$ with respect to the scalar product product $\langle {\cdot , \cdot }\rangle _{\rho _t}$. Consider the orthogonal decomposition

$$\begin{aligned} {\check{\rho }}_j \# \mathbf {B}_t = \nabla A_t + D_t \in \mathscr {G}\oplus \mathcal {D}_t \ . \end{aligned}$$

Since $\langle {\nabla {\widetilde{A}}, D_t}\rangle _{\rho _t} = 0$ for all ${\widetilde{A}} \in \mathcal {A}_0$, it follows that ${{\,\mathrm{div}\,}}({\widehat{\rho }}\# D_t) = 0$. Therefore, $\partial _t \rho _t + {{\,\mathrm{div}\,}}({\widehat{\rho }}_t\# \nabla A_t) = 0$. Moreover,

$$\begin{aligned} \tau \big [(\nabla A_t)^* {\widehat{\rho }}_t \# \nabla A_t \big ] = \Vert \nabla A_t \Vert _{\rho _t}^2 \le \Vert {\check{\rho }}_j \# \mathbf {B}_t \Vert _{\rho _t}^2 = \Vert \mathbf {B}_t \Vert _{-1,\rho _t}^2 \ , \end{aligned}$$

which yields the inequality “$\le$” in (9.3). $\square$

### Proposition 9.2

(Extension of the distance to the boundary) Suppose that $\theta _j(a,b) \ge C\min \{a,b\}^p$ for some $C > 0$ and $p<2$. Then the distance function $\mathscr {W}: {{\mathfrak {P}}}_+ \times {{\mathfrak {P}}}_+ \rightarrow {{\mathbb {R}}}$ extends continuously to a metric on ${{\mathfrak {P}}}$.

### Proof

Let $\rho _0, \rho _1 \in {{\mathfrak {P}}}$ and let $\{\rho _0^n\}_n, \{\rho _1^n\}_n$ be sequences in ${{\mathfrak {P}}}_+$ satisfying $\tau \big [ |\rho _i^n -\rho _i|^2 \big ] \rightarrow 0$ as $n \rightarrow \infty$ for $i = 0, 1$. We claim that the sequence $\{ \mathscr {W}(\rho _0^n, \rho _1^n) \}_n$ is Cauchy.

To prove this, it suffices to show that $\mathscr {W}(\rho _i^n, \rho _i^m) \rightarrow 0$ as $n, m\rightarrow \infty$ for $i=0,1$, since

$$\begin{aligned} |\mathscr {W}(\rho _0^n, \rho _1^n) - \mathscr {W}(\rho _0^m, \rho _1^m)| \le \mathscr {W}(\rho _0^n, \rho _0^m) + \mathscr {W}(\rho _1^n, \rho _1^m) \ . \end{aligned}$$

Fix $\varepsilon \in (0,1)$, and set ${\widetilde{\rho }} := (1- \varepsilon ) \rho _0 + \varepsilon {\mathbf{1}}$. Take $N \ge 1$ so large that $\tau \left[ |\rho _0^n - \rho _0|^2 \right] \le \varepsilon ^2$ whenever $n \ge N$. For $n \ge N$ we consider the linear interpolation $\rho ^n_t = (1-t) \rho _0^n + t {\widetilde{\rho }}$. Then ${\dot{\rho }}_t^n = {\widetilde{\rho }} - \rho _0^n$ for all $t \in (0,1)$. Since $\mathscr {K}_{\mathbf{1}}$ is invertible on $\mathcal {A}_0$ by Lemma 7.3 and ergodicity, we may define $A := \mathscr {K}_{\mathbf{1}}^{-1} ({\widetilde{\rho }} - \rho _0^n) \in \mathcal {A}_0$, and we have ${\dot{\rho }}_t^n = {{\,\mathrm{div}\,}}(\nabla A)$. Since $\rho ^n_t \ge t \varepsilon {\mathbf{1}}$ for $t \in [0,1]$, we have $\frac{1}{\theta _j}\big |_{{{\,\mathrm{sp}\,}}(\rho ^n_t)} \le C (t\varepsilon )^{-p}$, and thus $\tau [ (\nabla A)^* {\check{\rho }}_t^n \# \nabla A] \le C (t\varepsilon )^{-p} \tau [ |\nabla A|^2 ]$ by Lemma 6.6. It follows that

$$\begin{aligned} \mathscr {W}(\rho _0^n, {\widetilde{\rho }})&\le \int _0^1 \sqrt{ \tau [ (\nabla A)^* {\check{\rho }}_t^n \# \nabla A] } \; \mathrm {d}t \le C \varepsilon ^{-p/2} \Vert \nabla A\Vert _{L^2(\tau )} \ , \end{aligned}$$

since $p<2$. Using the boundedness of $\nabla \circ \mathscr {K}_{\mathbf{1}}^{-1}$ we obtain

$$\begin{aligned} C^{-1} \Vert \nabla A\Vert _{L^2(\tau )}&\le \Vert {\widetilde{\rho }} - \rho _0^n \Vert _{L^2(\tau )} \\&= \Vert \rho _0 - \rho _0^n \Vert _{L^2(\tau )} + \varepsilon \Vert {\mathbf{1}}- \rho _0 \Vert _{L^2(\tau )} \\&\le \varepsilon \big (1 + \Vert {\mathbf{1}}- \rho _0 \Vert _{L^2(\tau )}\big ) \ . \end{aligned}$$

We infer that $\mathscr {W}(\rho _0^n, {\widetilde{\rho }}) \le C \varepsilon ^{1-p/2}$ for some $C < \infty$ depending on $\rho _0$. It follows that $\mathscr {W}(\rho _0^n, \rho _0^m) \le C \varepsilon ^{1-p/2}$ for $n, m \ge N$. Since $p < 2$, this proves the claim.

We can thus extend $\mathscr {W}$ to ${{\mathfrak {P}}}$ by setting $\mathscr {W}(\rho _0, \rho _1) = \lim _{n \rightarrow \infty }\mathscr {W}(\rho _0^n, \rho _1^n)$. It immediately follows that $\mathscr {W}$ is symmetric and the triangle inequality extends to ${{\mathfrak {P}}}$. The fact that $\mathscr {W}(\rho _0, \rho _1) \ne 0$ whenever $\rho _0$ and $\rho _1$ are distinct, follows from Proposition 9.4 below. $\square$

Our next aim is to prove Proposition 9.4 below, which yields a lower bound on the distance $\mathscr {W}$ in terms of a non-commutative analogue of the 1-Kantorovich metric. To formulate the result, we use the notation

$$\begin{aligned} \Vert \mathbf {B}\Vert _{\mathcal {B},2} := \sqrt{\frac{1}{2}\sum _j \Vert \ell _j^\dagger ( B_j B_j^*) + {r}_j^\dagger (B_j^* B_j) \Vert _{\mathcal {A}} } \end{aligned}$$

for $$\mathbf {B}= (B_j)_{j \in \mathcal {J}} \in \mathcal {B}$$.

### Lemma 9.3

There exists $M < \infty$ such that $\Vert \mathbf {B}\Vert _\rho \le M \Vert \mathbf {B}\Vert _{\mathcal {B},2}$ for all $\rho \in {{\mathfrak {P}}}_+$ and $\mathbf {B}\in \mathcal {B}$. If $\theta _j(r,s) \le \frac{1}{2}(r+s)$ for all $r,s > 0$, then this estimate holds with $M = 1$:

$$\begin{aligned} \Vert \mathbf {B}\Vert _\rho \le \Vert \mathbf {B}\Vert _{\mathcal {B},2} \ . \end{aligned}$$

### Proof

Recalling that $\Vert \cdot \Vert _{\mathcal {B}_j}$ denotes the norm on $\mathcal {B}_j$, we define

$$\begin{aligned} M_j := \sup \Big \{ \tau _j[ \, | {\widehat{\rho }}_j \# B | \, ] \ : \rho \in {{\mathfrak {P}}}_+ \ , \ \Vert B\Vert _{\mathcal {B}_j} \le 1 \Big \} \ , \quad \text {and} \quad {\widetilde{M}} := \sup _{j \in \mathcal {J}} M_j \ . \end{aligned}$$

Since our setting is finite-dimensional, ${\widetilde{M}}$ is finite and all norms on $\mathcal {B}$ are equivalent. Thus, for a suitable constant $M < \infty$, it follows that

$$\begin{aligned} \Vert \mathbf {B}\Vert _{\rho }^2&= \sum _j \tau _j[B_j^* {\widehat{\rho }}_j \# B_j ] \le \sum _j \Vert B_j^*\Vert _{\mathcal {B}_j}\tau _j[ | {\widehat{\rho }}_j \# B_j |] \le {\widetilde{M}} \sum _j \Vert B_j\Vert _{\mathcal {B}_j}^2 \le M \Vert \mathbf {B}\Vert _{\mathcal {B},2}^2 \ , \end{aligned}$$

which proves the first statement.

Suppose now that $\theta _j(r,s) \le \frac{1}{2}(r+s)$. Since $\rho$ is positive and the operators $\ell _j$ and ${r}_j$ preserve positivity, we obtain using Lemma 6.6,

$$\begin{aligned} \Vert \mathbf {B}\Vert _{\rho }^2&= \sum _j \tau _j[B_j^* {\widehat{\rho }}_j \# B_j ] \\&\le \frac{1}{2}\sum _j \tau _j[ \ell _j(\rho ) B_j B_j^* + {r}_j(\rho ) B_j^* B_j ] \\&= \frac{1}{2}\sum _j \tau \big [\rho \big (\ell _j^\dagger ( B_j B_j^*) + {r}_j^\dagger (B_j^* B_j) \big ) \big ] \\&\le \frac{1}{2}\sum _j \Vert \ell _j^\dagger ( B_j B_j^*) + {r}_j^\dagger (B_j^* B_j) \Vert _{\mathcal {A}} \ , \end{aligned}$$

which yields the result. $\square$

For $\rho _0, \rho _1 \in {{\mathfrak {P}}}$ we set

9.4 $$\begin{aligned} W_1(\rho _0, \rho _1) := \sup \bigg \{ \tau [(\rho _1 - \rho _0)A] : A \in \mathcal {A}\ , \Vert \nabla A\Vert _{\mathcal {B},2} \le 1 \bigg \} \ . \end{aligned}$$

By analogy with the dual Kantorovich formulation of the commutative 1-Kantorovich metric $W_1$ in terms of Lipschitz functions, this metric can be seen as a non-commutative analogue of $W_1$. The following result generalizes a result from [18] from the discrete to the non-commutative setting; see also [46] for non-commutative results of this type.

### Proposition 9.4

Let *M* be as in Lemma 9.3 and set $N := \sup \{ \Vert \nabla A\Vert _{\mathcal {B},2} \ : \ \Vert A\Vert _\mathcal {A}\le 1 \}$. Then, for $\rho _0, \rho _1 \in {{\mathfrak {P}}}$ we have

$$\begin{aligned} N^{-1} \tau [ |\rho _0 - \rho _1 |] \le W_1(\rho _0, \rho _1) \le M \mathscr {W}(\rho _0,\rho _1) \ . \end{aligned}$$

### Proof

The first inequality follows from the definitions, since $\tau [|B|] = \sup _{\Vert A\Vert _\mathcal {A}\le 1} \tau [AB]$ for $B \in \mathcal {A}$.

Fix $\varepsilon > 0$, take ${\bar{\rho }}_0, {\bar{\rho }}_1 \in {{\mathfrak {P}}}$, and let $(\rho _t, B_t)_t$ be a solution to the continuity equation with approximately optimal action, i.e.,

$$\begin{aligned} \partial _t \rho _t + {{\,\mathrm{div}\,}}({\widehat{\rho }}_t \# \nabla B_t) = 0 \quad \mathrm{and}\quad \bigg (\int _0^1 \Vert \nabla B_t\Vert _{\rho _t}^2 \; \mathrm {d}t \bigg )^{\frac{1}{2}} \le \mathscr {W}({\bar{\rho }}_0, {\bar{\rho }}_1 ) + \varepsilon \;. \end{aligned}$$

For any $A \in \mathcal {A}_h$ we obtain using Lemma 9.3

$$\begin{aligned} \big | \tau [A ({\bar{\rho }}_0 - {\bar{\rho }}_1) ] \big |&= \bigg |\int _0^1 \tau [ A {\dot{\rho }}_t ] \; \mathrm {d}t\bigg | \\&= \bigg |\int _0^1 \tau [ A {{\,\mathrm{div}\,}}({\widehat{\rho }}_t \# \nabla B_t) ] \; \mathrm {d}t\bigg | \\&= \bigg |\int _0^1 \langle { \nabla A, \nabla B_t }\rangle _{\rho _t} \; \mathrm {d}t\bigg | \\&\le \bigg ( \int _0^1 \Vert \nabla A \Vert _{\rho _t}^2\; \mathrm {d}t \bigg )^{1/2} \bigg ( \int _0^1 \Vert \nabla B_t \Vert _{\rho _t}^2 \; \mathrm {d}t \bigg )^{1/2} \\&\le M \Vert \nabla A \Vert _{\mathcal {B},2} \big ( \mathscr {W}({\bar{\rho }}_0, {\bar{\rho }}_1) + \varepsilon \big ) \;. \end{aligned}$$

Since $\varepsilon > 0$ is arbitrary, the result follows by definition of $W_1$. $\square$

In the remainder of this section we impose the following natural additional conditions in addition to Assumption 7.2.

### Assumption 9.5

The functions $\theta _j: [0,\infty ) \times [0,\infty ) \rightarrow [0,\infty )$ are 1-homogeneous (which implies that $\theta _j(r,s) = s f_j(r/s)$ for some function $f_j$). The functions $f_j$ are assumed to be operator monotone.

Under this assumption, we will prove some crucial convexity properties for the action functional and the squared distance.

### Proposition 9.6

(Convexity of the action) Let $\rho ^i\in {{\mathfrak {P}}}$ and $\mathbf {B}^i\in \mathcal {B}$ for $i=0,1$. For $s\in [0,1]$ set $\rho ^s := (1-s) \rho ^0 + s \rho ^1$ and $\mathbf {B}^s := (1-s) \mathbf {B}^0 + s \mathbf {B}^1$. Then we have

$$\begin{aligned} \mathscr {A}(\rho ^s,\mathbf {B}^s) \le (1-s)\mathscr {A}(\rho ^0, \mathbf {B}^0) + s \mathscr {A}(\rho ^1,\mathbf {B}^1)\ . \end{aligned}$$

### Proof

This follows immediately from Theorem 8.3 in view of Assumption 9.5. $\square$

### Theorem 9.7

(Convexity of the squared distance) For $i = 0, 1$, let $\rho _0^i, \rho _1^i \in {{\mathfrak {P}}}$, and for $s \in [0,1]$ set $\rho _0^s := (1-s) \rho _0^0 + s \rho _0^1$ and $\rho _1^s := (1-s) \rho _1^0 + s \rho _1^1$. Then:

$$\begin{aligned} \mathscr {W}(\rho _0^s,\rho _1^s)^2 \le (1 - s) \mathscr {W}(\rho _0^0, \rho _1^0)^2 + s \mathscr {W}(\rho _0^1, \rho _1^1)^2\ . \end{aligned}$$

### Proof

Fix $\varepsilon > 0$. By continuity, it suffices to prove the inequality for $\rho _0^i, \rho _1^i \in {{\mathfrak {P}}}_+$ and $i = 0,1$. Let $(\rho ^i_t, \mathbf {B}^i_t)_t$ be such that $\partial _t \rho _t^i + {{\,\mathrm{div}\,}}\mathbf {B}_t^i = 0$ and $\int _0^1 \mathscr {A}(\rho _t^i, \mathbf {B}_t^i) \; \mathrm {d}t \le \mathscr {W}(\rho _0^i, \rho _1^i)^2 + \varepsilon$. For $s \in [0,1]$ we define

$$\begin{aligned} \rho _t^s := (1-s) \rho _t^0 + s \rho _t^1 \quad \text { and } \quad \mathbf {B}_t^s := (1-s) \mathbf {B}_t^0 + s \mathbf {B}_t^1 \ . \end{aligned}$$

It follows that $\partial _t \rho _t^s + {{\,\mathrm{div}\,}}\mathbf {B}_t^s = 0$, and by Lemma 9.1 and Proposition 9.6 we obtain

$$\begin{aligned} \mathscr {W}(\rho _0^s,\rho _1^s)^2&\le \int _0^1 \mathscr {A}(\rho _t^s, \mathbf {B}_t^s) \; \mathrm {d}t \\&\le (1-s) \int _0^1 \mathscr {A}(\rho _t^0, \mathbf {B}_t^0) \; \mathrm {d}t + s \int _0^1 \mathscr {A}(\rho _t^1, \mathbf {B}_t^1) \; \mathrm {d}t \\&\le (1 - s) \mathscr {W}(\rho _0^0, \rho _1^0)^2 + s \mathscr {W}(\rho _0^1, \rho _1^1)^2 + 2 \varepsilon \ . \end{aligned}$$

Since $\varepsilon > 0$ is arbitrary, the desired inequality follows. $\square$

Using these convexity properties, the existence of constant speed geodesics for the metric $\mathscr {W}$ follows by standard arguments; cf. [18, Theorem 3.2]) for a proof in the commutative setting and [46] for a proof in a non-commutative context.

### Theorem 9.8

(Existence of $\mathscr {W}$-geodesics) For any ${\bar{\rho }}_0, {\bar{\rho }}_1 \in {{\mathfrak {P}}}$ there exists a curve $\rho : [0,1] \rightarrow {{\mathfrak {P}}}$ satisfying $\rho _0 = {\bar{\rho }}_0$, $\rho _1 = {\bar{\rho }}_1$, and $\mathscr {W}(\rho _s, \rho _t) = | s-t | \mathscr {W}(\rho _0, \rho _1)$ for all $s, t \in [0,1]$.

## Geodesic Convexity of the Entropy

In this section we will analyse geodesic convexity of the relative entropy functional ${{\,\mathrm{Ent}\,}}_\sigma$. Throughout this section we fix a differential structure $(\mathcal {A}, \nabla , \sigma )$ and assume that the associated quantum Markov semigroup $(\mathscr {P}_t)$ is ergodic. We consider the transport metric $\mathscr {W}$ defined in Theorem 7.7 using the functions $\theta _j$ given by $\theta _j(r,s) := \Lambda (e^{\omega _j/2}r, e^{-\omega _j/2}s)$, so that the Kolmogorov forward equation $\partial _t \rho = \mathscr {L}^\dagger \rho$ is the gradient flow of the relative von Neumann entropy ${{\,\mathrm{Ent}\,}}_\sigma$ with respect to the Riemannian metric induced by $(\mathscr {K}_\rho )_\rho$.

The following terminology will be useful.

### Definition 10.1

Let $(\mathcal {X},d)$ be a metric space. A functional $\mathcal {F}: \mathcal {X}\rightarrow {{\mathbb {R}}}\cup \{ + \infty \}$ is said to be

- *weakly geodesically* $\lambda$*-convex* if any pair $x_0, x_1 \in \mathcal {X}$ can be connected by a geodesic $(\gamma _t)_{t \in [0,1]}$ in $(\mathcal {X}, d)$ along which $\mathcal {F}$ satisfies the $\lambda$-convexity inequality
  10.1 $$\begin{aligned} \mathcal {F}(\gamma _t) \le (1-t) \mathcal {F}(\gamma _0) + t \mathcal {F}(\gamma _1) - \frac{\kappa }{2} t(1-t) d(x_0, x_1)^2\ . \end{aligned}$$
- *strongly geodesically* $\lambda$*-convex* if (10.1) holds for any geodesic $(\gamma _t)_{t \in [0,1]}$ in $(\mathcal {X}, d)$.

The following result, shows in particular that these concepts are equivalent in our setting and provides several equivalent characterizations of geodesic $\lambda$-convexity. We shall use the notation

$$\begin{aligned} \frac{\mathrm {d}^+}{\mathrm {d}t}f(t) = \limsup _{h \downarrow 0} \frac{f(t+h) - f(t)}{h}\ . \end{aligned}$$

We refer to [18] for a version of this result in the discrete setting, and to [46] for the Lindblad setting.

### Theorem 10.2

(Characterizations of geodesic $\lambda$-convexity) Let $\lambda \in {{\mathbb {R}}}$. For a differential structure $(\mathcal {A}, \nabla , \sigma )$ the following assertions are equivalent:

1. ${{\,\mathrm{Ent}\,}}_\sigma$ is weakly geodesically $\lambda$-convex on $({{\mathfrak {P}}},\mathscr {W})$;
2. ${{\,\mathrm{Ent}\,}}_\sigma$ is strongly geodesically $\lambda$-convex on $({{\mathfrak {P}}},\mathscr {W})$;
3. For all $\rho , \nu \in {{\mathfrak {P}}}$, the following ‘evolution variational inequality’ holds for all $t \ge 0$:
  10.2 $$\begin{aligned} \frac{1}{2}\frac{\mathrm {d}^+}{\mathrm {d}t}\mathscr {W}^2(\mathscr {P}_t^\dagger \rho , \nu ) + \frac{\lambda }{2} \mathscr {W}^2(\mathscr {P}_t^\dagger \rho , \nu ) \le {{\,\mathrm{Ent}\,}}_\sigma (\nu ) - \mathcal {H}(\mathscr {P}_t^\dagger \rho )\;; \end{aligned}$$
4. For all $\rho \in {{\mathfrak {P}}}_+$ and $A \in \mathcal {A}_0$ we have
  $$\begin{aligned} {{\,\mathrm{Hess}\,}}_\mathscr {K}{{\,\mathrm{Ent}\,}}_\sigma (\rho )[A, A] \ge \lambda \tau [A \mathscr {K}_\rho A] \ . \end{aligned}$$

### Proof

“$(4) \Rightarrow (3)$” This can be proved by an argument from [14]; see [18, Theorem 4.5] for a proof in a similar setting.

“$(3) \Rightarrow (2)$”: This follows from an application of [14, Theorem 3.2] to the metric space $({{\mathfrak {P}}}, \mathscr {W})$.

“$(2) \Rightarrow (1)$”: Since $({{\mathfrak {P}}}, \mathscr {W})$ is a geodesic space, this implication is immediate.

“$$(1) \Rightarrow (4)$$”: Obvious. $$\square $$

In the classical setting, the Ricci curvature on a Riemannian manifold $\mathscr {M}$ is bounded from below by $\lambda \in {{\mathbb {R}}}$ if and only if the entropy (with respect to the volume measure) is geodesically $\lambda$-convex in the space of probability measures $\mathscr {P}(\mathscr {M})$ endowed with the Kantorovich metric $W_2$. This characterisation is the starting point for the synthetic theory of metric measure spaces with lower Ricci curvature bounds, which has been pioneered by Lott, Sturm and Villani.

By analogy, we make the following definition in the non-commutative setting, which extends the corresponding definition in the discrete setting [18].

### Definition 10.3

(*Ricci curvature*) Let $\lambda \in {{\mathbb {R}}}$. We say that a differential structure $(\mathcal {A}, \nabla , \sigma )$ has Ricci curvature bounded from below by $\lambda$ if the equivalent conditions of Theorem 10.2 hold. In this case, we write ${{\,\mathrm{Ric}\,}}(\mathcal {A}, \nabla , \sigma ) \ge \lambda$.

It is possible to characterize Ricci curvature in terms of a gradient estimate in the spirit of Bakry–Émery; see [17] for the corresponding statement in the setting of finite Markov chains and [46] for an implementation in the Lindblad setting.

### Theorem 10.4

(Gradient estimate) Let $\lambda \in {{\mathbb {R}}}$. A differential structure $(\mathcal {A}, \nabla , \sigma )$ satisfies ${{\,\mathrm{Ric}\,}}(\mathcal {A}, \nabla , \sigma ) \ge \lambda$ if and only if the following gradient estimate holds for all $\rho \in {{\mathfrak {P}}}$, $A \in \mathcal {A}_0$ and $t \ge 0$:

10.3 $$\begin{aligned} \Vert \nabla \mathscr {P}_t A \Vert _{\rho }^2 \le e^{-2 \lambda t} \Vert \nabla A \Vert _{\mathscr {P}_t^\dagger \rho }^2 \ . \end{aligned}$$

### Proof

We follow a standard semigroup interpolation argument. Clearly, (10.3) holds for any $\rho \in {{\mathfrak {P}}}$ if and only if it holds for any $\rho \in {{\mathfrak {P}}}_+$.

Fix $t > 0$, $\rho \in {{\mathfrak {P}}}_+$ and $A \in \mathcal {A}_0$, and define $f: [0,t] \rightarrow {{\mathbb {R}}}$ by

$$\begin{aligned} f(s) := e^{-2 \lambda s} \langle {\mathscr {K}_{\mathscr {P}_s^\dagger \rho } \mathscr {P}_{t-s} A, \mathscr {P}_{t-s} A}\rangle _{L^2(\mathcal {A},\tau )} = e^{-2 \lambda s} \Vert \nabla \mathscr {P}_{t-s} A \Vert _{\mathscr {P}_s^\dagger \rho }^2 \ . \end{aligned}$$

Writing $\rho _s = \mathscr {P}_s^\dagger \rho$ and $A_s = \mathscr {P}_s A$, it follows by (7.17) and Proposition 7.16 that

$$\begin{aligned} f'(s)&= e^{-2\lambda s} \tau \big [ (\nabla A_{t-s})^* (\mathcal {N}^{(1)}_{\rho _s, \mathscr {L}^\dagger \rho _s} + \mathcal {N}^{(2)}_{\rho _s, \mathscr {L}^\dagger \rho _s}) \# \nabla A_{t-s} \\&\quad - 2 (\nabla \mathscr {L}A_{t-s})^* {\widehat{\rho }}_s \# \nabla A_{t-s} - 2 \lambda (\nabla A_{t-s})^* {\widehat{\rho }}_s \# \nabla A_{t-s} \big ] \\&= 2 e^{-2\lambda s} \Big ( {{\,\mathrm{Hess}\,}}_\mathscr {K}{{\,\mathrm{Ent}\,}}_\sigma (\rho _s)[A_{t-s}, A_{t-s}] - \lambda \tau [A_{t-s} \mathscr {K}_{\rho _s} A_{t-s}] \Big ) \ . \end{aligned}$$

Assume now that ${{\,\mathrm{Ric}\,}}(\mathcal {A}, \nabla , \sigma )$. Applying (4) from Theorem 10.2, we obtain $f'(s) \ge 0$ for all *s*. This implies that $f(t) \ge f(0)$, which is (10.3).

To prove the converse, set $g(t) = e^{2\lambda t}\Vert \nabla \mathscr {P}_t A \Vert _{\rho }^2$ and $h(t) = \Vert \nabla A \Vert _{\mathscr {P}_t^\dagger \rho }^2$. Then (10.3) implies hat $g(t) \le h(t)$ for all $t \ge 0$. Since $g(0) = h(0)$, we infer that $g'(0) \le h'(0)$. Since

$$\begin{aligned} g'(0)&= 2 \tau \big [ (\nabla \mathscr {L}A)^* {\widehat{\rho }}\# \nabla A\big ] + 2 \lambda \Vert A \Vert _{\rho }^2 \ , \\ h'(0)&= \tau \Big [(\nabla A)^* \big (\mathcal {N}_{\rho ,\mathscr {L}^\dagger \rho }^{(1)} + \mathcal {N}_{\rho ,\mathscr {L}^\dagger \rho }^{(2)}\big ) \# \nabla A \Big ] \ , \end{aligned}$$

we obtain ${{\,\mathrm{Hess}\,}}_\mathscr {K}{{\,\mathrm{Ent}\,}}_\sigma (\rho )[A, A] \ge \lambda \tau [A \mathscr {K}_\rho A]$ in view of the expression for the Hessian in Proposition 7.16. $\square$

An immediate consequence of a Ricci curvature bound is the following contractivity estimate for the associated semigroup, which was independently proved by Rouzé in [44].

### Proposition 10.5

($\lambda$-Contractivity) If ${{\,\mathrm{Ric}\,}}(\mathcal {A}, \nabla , \sigma ) \ge \lambda$, then the $\lambda$-contractivity bound

$$\begin{aligned} \mathscr {W}(\mathscr {P}_t^\dagger \rho _0, \mathscr {P}_t^\dagger \rho _1) \le e^{-\lambda t} \mathscr {W}(\rho _0, \rho _1) \end{aligned}$$

holds for all $\rho _0, \rho _1 \in {{\mathfrak {P}}}$ and $t \ge 0$.

### Proof

This is a well-known consequence of the evolution variational inequality (10.2); see [14, Proposition 3.1]. $\square$

Using the techniques developed in this paper, we can explicitly compute the Ricci curvature for the depolarizing channel defined in Sect. 5.6. The result has been obtained independently by Rouzé in [44].

### Theorem 10.6

(Ricci bound for the depolarizing channel) Let $\gamma > 0$, and let $(\mathcal {A}, \nabla , \tau )$ be a differential structure for the generator of the depolarizing channel given by $\mathscr {L}A = \gamma (\tau [A]{\mathbf{1}}- A)$. Then ${{\,\mathrm{Ric}\,}}(\mathcal {A}, \nabla , \tau ) \ge \gamma$.

### Proof

Since $\mathscr {L}A = \gamma (\tau [A]{\mathbf{1}}- A)$ and $\partial _j {\mathbf{1}}= 0$, we have $\partial _j \mathscr {L}A = - \gamma \partial _j A$, independently of the choice of the operators $\partial _j$. We will show that the result follows from this identity.

First we note that

10.4 $$\begin{aligned} - \tau [( \nabla \mathscr {L}A)^* {\widehat{\rho }}\# \nabla A] = \gamma \tau [( \nabla A)^* {\widehat{\rho }}\# \nabla A] \ . \end{aligned}$$

Moreover, since $\partial _1\Lambda (a,b) = \int _0^1 (1-s) a^{-s} b^s \; \mathrm {d}s$ we obtain (using the notation from (7.16)),Similarly, we have $\mathcal {N}_{\rho ,\mathscr {L}\rho }^{(2),j} = \gamma \big ( {\mathbf{1}}\otimes ({\mathbf{1}}- \rho ) \big )\partial _2\Lambda (\rho , \rho )$. Using the scalar identity $a\partial _1 \Lambda (a,b) + b\partial _2 \Lambda (a,b) = \Lambda (a,b)$, it follows that

$$\begin{aligned} \mathcal {N}_{\rho ,\mathscr {L}\rho }^{(1),j} + \mathcal {N}_{\rho ,\mathscr {L}\rho }^{(2),j} = \gamma (\partial _1\Lambda + \partial _2\Lambda - \Lambda )(\rho , \rho ) \ . \end{aligned}$$

Moreover, we note that $\partial _1 \Lambda (a,b) + \partial _2 \Lambda (a,b) \ge 1 \ge \Lambda (a,b)$ for $a, b \in [0,1]$ (and hence for $a,b \in {{\,\mathrm{sp}\,}}(\rho )$). Therefore, for $\eta =1 , 2$, we obtain using Lemma 6.6,

10.5 $$\begin{aligned} \begin{aligned} \tau \big [(\nabla A)^* \mathcal {N}_{\rho ,\mathscr {L}^\dagger \rho }^{(\eta )} \# (\nabla A) \big ]&= \frac{1}{2} \tau \big [(\nabla A)^* \big (\mathcal {N}_{\rho ,\mathscr {L}^\dagger \rho }^{(1)} + \mathcal {N}_{\rho ,\mathscr {L}^\dagger \rho }^{(2)}\big ) \# (\nabla A) \big ] \\&= \frac{\gamma }{2} \tau \big [(\nabla A)^* (\partial _1 \Lambda + \partial _2 \Lambda - \Lambda )(\rho , \rho ) \# (\nabla A) \big ] \\&\ge 0 \ . \end{aligned} \end{aligned}$$

Combining (10.4) and (10.5), it follows from (7.19) that

$$\begin{aligned} {{\,\mathrm{Hess}\,}}_\mathscr {K}{{\,\mathrm{Ent}\,}}(\rho )[A, A] \ge \gamma \tau [(\nabla A)^* {\widehat{\rho }}\# \nabla A] = \gamma \langle {\mathscr {K}_\rho A, A}\rangle _{L^2(\tau )} \ , \end{aligned}$$

which proves the result. $\square$

Since the spectral gap of $\mathscr {L}$ equals $\gamma$, it follows from the results in Sect. 11 that the obtained constant is optimal.

### Geodesic Convexity Via Intertwining

In this subsection we provide a useful technique for proving Ricci curvature bounds, which has the advantage that it does not require an explicit computation of the Hessian of the entropy. Instead, it relies on the following intertwining property between the gradient and the quantum Markov semigroup.

#### Definition 10.7

(*Intertwining property*) For $\lambda \in {{\mathbb {R}}}$, we say that a collection of linear operators $(\mathbf {\mathscr {P}_t})_{t \ge 0}$ on $\mathcal {B}$ is $\lambda$-intertwining for the quantum Markov semigroup $(\mathscr {P}_t)_{t \ge 0}$, if the following conditions hold:

1. For all $A \in \mathcal {A}$ and $t \ge 0$, we have $\nabla \mathscr {P}_t A = \mathbf {\mathscr {P}_t} \nabla A$;
2. For all $\rho \in {{\mathfrak {P}}}_+$, $\mathbf {B}= (B_j) \in \mathcal {B}$ and $t \ge 0$, we have
  10.6 $$\begin{aligned} \mathscr {A}\big (\rho , \mathbf {\mathscr {P}_t^\dagger } \mathbf {B}\big ) \le e^{-2\lambda t } \mathscr {A}\big (\rho , (\mathscr {P}_t^\dagger B_j)_j \big ) \ . \end{aligned}$$

By duality, the intertwining relation (1) implies the identity

10.7 $$\begin{aligned} \mathscr {P}_t {{\,\mathrm{div}\,}}(\mathbf {A}) = {{\,\mathrm{div}\,}}(\mathbf {\mathscr {P}_t^\dagger } \mathbf {A})\ , \qquad \text {for } \mathbf {A}\in \mathcal {B}. \end{aligned}$$

The following lemma allows us to check the $\lambda$-intertwining property in several examples of interest.

#### Lemma 10.8

Let $\lambda \in {{\mathbb {R}}}$, and suppose that $\partial _j \mathscr {L}A = (\mathscr {L}- \lambda ) \partial _j A$ for all $A \in \mathcal {A}$. Then the semigroup $(\mathbf {\mathscr {P}_t})_t$ defined by $(\mathbf {\mathscr {P}_t} \mathbf {B})_j = e^{-\lambda t} \mathscr {P}_t B_j$ is $\lambda$-intertwining for the quantum Markov semigroup $(\mathscr {P}_t)_{t \ge 0}$.

#### Proof

By spectral theory, the stated condition on the generator is equivalent to the semigroup property $\partial _j \mathscr {P}_t A = e^{-\lambda t} \mathscr {P}_t \partial _j A$ for all $t \ge 0$. Thus, the semigroup $(\mathbf {\mathscr {P}_t})_t$ satisfies (1) in Definition 10.7. Since $(\mathbf {\mathscr {P}_t^\dagger } \mathbf {B})_j = e^{-\lambda t} \mathscr {P}_t^\dagger B_j$, condition (2) follows as well. $\square$

#### Theorem 10.9

(Lower Ricci bound via intertwining) Let $(\mathcal {A}, \nabla , \sigma )$ be a differential structure, and let $\lambda \in {{\mathbb {R}}}$. If there exists a collection of linear operators $(\mathbf {\mathscr {P}_t})_{t \ge 0}$ on $\mathcal {B}$ that is $\lambda$-intertwining for the associated QMS $(\mathscr {P}_t)_{t \ge 0}$, then ${{\,\mathrm{Ric}\,}}(\mathcal {A}, \nabla , \sigma ) \ge \lambda$.

#### Proof of Theorem 10.9

The proof is a variation on an argument by Dolbeault, Nazaret and Savaré [16].

Fix ${\bar{\rho }}, \nu \in {{\mathfrak {P}}}$, and let $(\rho _s, \mathbf {B}_s)_{s \in [0,1]}$ be a solution to the continuity equation

$$\begin{aligned} \partial _s \rho _s + {{\,\mathrm{div}\,}}\mathbf {B}_s = 0\ , \qquad \rho _0 = \nu \ , \quad \rho _1 = {\bar{\rho }}\ , \end{aligned}$$

that minimizes the action functional (9.3). This implies that $(\rho _s)_s$ is a constant speed geodesic, and

10.8 $$\begin{aligned} \mathscr {A}(\rho _s, \mathbf {B}_{s}) = \mathscr {W}(\nu , {\bar{\rho }})^2 \end{aligned}$$

for all $s \in [0,1]$. We define $\rho _s^t := \mathscr {P}_{st}^\dagger \rho _s$, so that $\partial _s \rho _s^t = \mathscr {P}_{st}^\dagger (\partial _s \rho _s) - t \mathscr {L}^\dagger \mathscr {P}_{st}^\dagger \rho _s$. Using this identity, we obtain

$$\begin{aligned} \partial _s \rho _s^t&= \mathscr {P}_{st}^\dagger (\partial _s \rho _s) - t \mathscr {L}^\dagger \mathscr {P}_{st}^\dagger \rho _s \\&= - \mathscr {P}_{st}^\dagger ({{\,\mathrm{div}\,}}\mathbf {B}_s) - t \mathscr {L}^\dagger \rho _s^t \\&= - {{\,\mathrm{div}\,}}(\mathbf {\mathscr {P}_{st}^\dagger } \mathbf {B}_s) - t \mathscr {L}^\dagger \rho _s^t\ . \end{aligned}$$

Write ${\widetilde{\nabla }} = ({\widetilde{\partial }}_j)_j$, where ${\widetilde{\partial }}_j = e^{-\omega _j/2}V_j {r}_j(\rho ) - e^{\omega _j/2}\ell _j(\rho ) V_j$. It then follows from Lemma 7.8 and Theorem 7.7 that $\mathscr {L}^\dagger = {{\,\mathrm{div}\,}}{\widetilde{\nabla }}$. Hence, we infer that the curve $(\rho _s^t)_{s \in [0,1]}$ satisfies the continuity equation $\partial _s \rho _s^t + {{\,\mathrm{div}\,}}\mathbf {B}_s^t = 0$, where

$$\begin{aligned} \mathbf {B}_s^t = \mathbf {\mathscr {P}_{st}^\dagger }\mathbf {B}_s - t {\widetilde{\nabla }} \rho _s^t\ . \end{aligned}$$

Using the bilinearity of $\mathcal {A}(\rho _s^t, \cdot , \cdot )$, we obtain

10.9 $$\begin{aligned} \begin{aligned} \mathscr {W}(\nu , \mathscr {P}_t^\dagger {\bar{\rho }})^2&\le \int _0^1 \mathscr {A}(\rho _s^t, \mathbf {B}_s^t) \; ds \\&= \int _0^1 \mathscr {A}(\rho _s^t, \mathbf {\mathscr {P}_{st}^\dagger }\mathbf {B}_s) - 2t \mathscr {A}(\rho _s^t, \mathbf {B}_s^t, {\widetilde{\nabla }} \rho _s^t) - t^2 \mathscr {A}(\rho _s^t, {\widetilde{\nabla }} \rho _s^t) \; \mathrm {d}s\ . \end{aligned} \end{aligned}$$

Using (10.6) and Corollary 8.7 we infer that

$$\begin{aligned} \mathscr {A}(\rho _s^t, \mathbf {\mathscr {P}_{st}^\dagger }\mathbf {B}_s)&\le e^{-2\lambda st} \mathscr {A}\big (\mathscr {P}_{st}^\dagger \rho _s, (\mathscr {P}_{st}^\dagger B_{j,s})_j\big ) \le e^{-2\lambda st} \mathscr {A}\big (\rho _s, \mathbf {B}_s\big ) \end{aligned}$$

hence (10.8) yields

$$\begin{aligned} \int _0^1 \mathscr {A}(\rho _s^t, \mathbf {\mathscr {P}_{st}^\dagger }\mathbf {B}_s) \; \mathrm {d}s \le \frac{1 - e^{-2\lambda t}}{2\lambda t} \mathscr {W}(\nu , {\bar{\rho }})^2 \ , \end{aligned}$$

A direct computation using Lemma 7.8 shows that

$$\begin{aligned} \partial _s {{\,\mathrm{Ent}\,}}_\sigma (\rho _s^t)&= \tau [ (\log \rho _s^t - \log \sigma ) \partial _s \rho _s^t ] = - \tau [(\log \rho _s^t - \log \sigma ) {{\,\mathrm{div}\,}}\mathbf {B}_s^t ] \\&= \tau [ (\nabla (\log \rho _s^t - \log \sigma ))^* \mathbf {B}_s^t ] = \tau [ ({\check{\rho }}_s^t \# {\widetilde{\nabla }} \rho _s^t)^* \mathbf {B}_s^t ] = \mathcal {A}(\rho _s^t; \mathbf {B}_s^t, {\widetilde{\nabla }} \rho _s^t) \ . \end{aligned}$$

Estimating the final term in (10.9) by 0, we infer that

$$\begin{aligned} \frac{1}{2t} \Big (\mathscr {W}(\nu , \mathscr {P}_t^\dagger {\bar{\rho }})^2 - \mathscr {W}(\nu , {\bar{\rho }})^2\Big ) \le \frac{1}{2t}\Big ( \frac{1 - e^{-2\lambda t}}{2\lambda t} - 1\Big ) \mathscr {W}(\nu , {\bar{\rho }})^2 - \int _0^1 \partial _s {{\,\mathrm{Ent}\,}}_\sigma (\rho _s^t) \; \mathrm {d}s\ . \end{aligned}$$

Since $t \mapsto {{\,\mathrm{Ent}\,}}_\sigma (\rho _s^t)$ is continuous, we observe that the right-hand side converges as $t \downarrow 0$. Letting $t \downarrow 0$ we infer that

$$\begin{aligned} \frac{1}{2}\frac{\mathrm {d}^+}{\mathrm {d}t}\bigg |_{t = 0}\ \mathscr {W}(\nu , \mathscr {P}_t {\bar{\rho }})^2 \le \frac{\lambda }{2} \mathscr {W}(\nu , {\bar{\rho }})^2 + {{\,\mathrm{Ent}\,}}_\sigma ({\bar{\rho }}) - {{\,\mathrm{Ent}\,}}_\sigma (\nu )\ , \end{aligned}$$

which proves the evolutional variational inequality from Theorem 10.2 for $t = 0$. By the semigroup property, the inequality holds for all $t \ge 0$, hence the result follows. $\square$

#### Remark 10.10

As pointed out by an anonymous referee, the condition from Lemma 10.8 is preserved under taking tensor products of quantum Markov semigroups. Therefore, Theorem 10.9 yields a lower Ricci curvature bound for tensor product semigroups of this type. It is an interesting open question whether such a tensorisation property holds for arbitrary quantum Markov semigroups, as is known to be true in the Markov chain setting [18].

We finish the section with the example of the Fermionic Ornstein–Uhlenbeck equation from Sect. 5.5, which was already discussed in [10]. For the convenience of the reader we provide the details.

#### Proposition 10.11

(Intertwining for fermions) In the fermionic setting, we have the commutation relations $[\partial _j, \mathscr {L}] = - \partial _j$ for $j = 1, \ldots , n$. Consequently, the intertwining property holds with $\lambda = 1$.

#### Proof

We use the well-known fact that the differential operator $\partial _j$ is the *annihilation operator*: it maps the *k*-particle space $\mathcal {H}^k$ into the $(k-1)$-particle space $\mathcal {H}^{k-1}$ for any $0 \le k \le n$ (with the convention that $\mathcal {H}^{-1} = \{0\})$. On the other hand, $-\mathscr {L}$ is the *number operator*, which satisfies $\mathscr {L}A = - k A$ for all $A \in \mathcal {H}^k$. Hence, for $A \in \mathcal {H}^{k}$, we have $\partial _j \mathscr {L}A = - k \partial _j A$, whereas $\mathscr {L}\partial _j A = - (k-1)\partial _j A$. This yields the desired commutation relation $[\partial _j, \mathscr {L}] = - \partial _j$ on $\mathcal {H}^{k}$, which extends to $\mathfrak {C}^n$ by linearity. The result thus follows from Lemma 10.8. $\square$

We immediately obtain the following result.

#### Corollary 10.12

The differential structure for the fermionic Ornstein–Uhlenbeck equation in Sect. 5.5 satisfies ${{\,\mathrm{Ric}\,}}(\mathfrak {C}^n, \nabla , \tau ) \ge 1$ in any dimension $n \ge 1$.

It follows from the results in the following section that the constant 1 is optimal.

## Functional Inequalities

One of the advantages of the framework of this paper is that it allows one to prove a sequence of implications between several useful functional inequalities. Throughout this section we assume that $(\mathscr {P}_t)_t$ is ergodic.

Recall that

$$\begin{aligned} {{\,\mathrm{Ent}\,}}_\sigma (\rho ) := {{\,\mathrm{Tr}\,}}[\rho ( \log \rho - \log \sigma ) ]\ ,\qquad \mathcal {I}_\sigma (\rho ) := -{{\,\mathrm{Tr}\,}}[(\log \rho - \log \sigma )\mathscr {L}^\dagger \rho ] \ , \end{aligned}$$

and note that $\frac{\mathrm {d}}{\mathrm {d}t}{{\,\mathrm{Ent}\,}}_\sigma (\mathscr {P}_t^\dagger \rho ) = - \mathcal {I}_\sigma (\mathscr {P}_t^\dagger \rho )$ for $\rho \in {{\mathfrak {P}}}_+$. The quantity $\mathcal {I}_\sigma$ is a quantum version of the Fisher information (or entropy production) relative to $\sigma$; we refer to [42] for an introduction to several notions of Fisher information in the quantum setting.

The gradient flow structure from Theorem 7.7 implies that $\mathscr {L}^\dagger \rho = {{\,\mathrm{div}\,}}({\widehat{\rho }}\#\nabla (\log \rho - \log \sigma ) )$, which yields $\mathcal {I}_\sigma (\rho ) = \Vert \nabla (\log \rho - \log \sigma )\Vert _\rho ^2$. Recall that for $\rho \in {{\mathfrak {P}}}$ and $A \in \mathcal {A}$ we denote the associated Bogolioubov–Kubo–Mori scalar product and norm by

$$\begin{aligned} \langle {A, B}\rangle _{L^2_\mathrm{BKM}(\rho )} = \int _0^1 \tau \big [A^* \rho ^{1-s} B \rho ^s \big ] \; \mathrm {d}s \ , \qquad \Vert A\Vert _{L^2_\mathrm{BKM}(\rho )} = \sqrt{\langle {A, A}\rangle _{ BKM} } \ . \end{aligned}$$

The results presented in this section have been obtained in the classical discrete setting of finite Markov chains in [18], and in the setting of Lindblad operators in [46]. Here we state and prove the results in the more general framework that includes arbitrary differential structures $(\mathcal {A}, \nabla , \sigma )$. The proofs closely follow the original arguments by Otto and Villani [39], which were adapted in [18, 46]. In our finite-dimensional setting, most of the results follow directly from Riemannian considerations, though some additional care is needed due to the degeneracy of the metric at the boundary ${{\mathfrak {P}}}{\setminus } {{\mathfrak {P}}}_+$.

### Definition 11.1

A differential structure $(\mathcal {A}, \nabla , \sigma )$ satisfies

1. a *modified logarithmic Sobolev inequality* with constant $\lambda >0$ if for all $\rho \in \mathscr {P}(\mathcal {X})$, 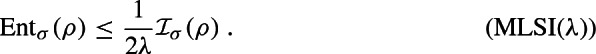
2. an $H\mathscr {W}I$ *inequality* with constant $\kappa \in {{\mathbb {R}}}$ if for all $\rho \in \mathscr {P}(\mathcal {X})$, 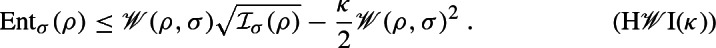
3. a *modified Talagrand inequality* with constant $\lambda > 0$ if for all $\rho \in {{\mathfrak {P}}}$, 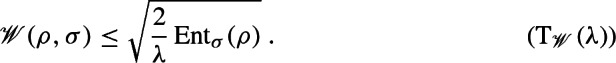
4. a $T_1$*-transport inequality* with constant $\lambda > 0$ if for all $\rho \in {{\mathfrak {P}}}$, 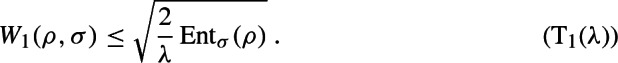
5. a *Poincar*é *inequality* (or *spectral gap inequality*) with constant $\lambda > 0$ if for all $A \in \mathcal {A}_h$ with $\tau [\int _0^1 \sigma ^{1-s} A \sigma ^s \; \mathrm {d}s] = 0$, 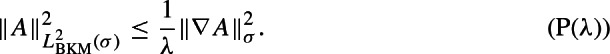

It is well known and an easy consequence of Gronwall’s inequality, that MLSI($\lambda$) is equivalent to the exponential decay of the entropy with rate $2\lambda$:

11.1 $$\begin{aligned} {{\,\mathrm{Ent}\,}}_\sigma (\mathscr {P}_t^\dagger \rho ) \le e^{-2\lambda t} {{\,\mathrm{Ent}\,}}_\sigma (\rho )\ . \end{aligned}$$

There are other approaches to some of these inequalites and variants of them; see, e.g., [3, 4, 9, 30, 41].

Recall that for an absolutely continuous curve $(\rho _t)_t \in ({{\mathfrak {P}}}, \mathscr {W})$, its *metric derivative*

$$\begin{aligned} \vert {\rho _t'}\vert := \lim _{h\rightarrow 0}\frac{\mathscr {W}(\rho _{t+h},\rho _t)}{\vert {h}\vert } \end{aligned}$$

exists for a.e. $t\in [0,T]$; see [2, Theorem 1.1.2].

### Proposition 11.2

Let $\rho ,\nu \in {{\mathfrak {P}}}_+$. For all $t \ge 0$ we have

11.2 $$\begin{aligned} \frac{\mathrm {d}^+}{\mathrm {d}t}\mathscr {W}(\mathscr {P}_t^\dagger \rho ,\nu ) \le \sqrt{\mathcal {I}_\sigma (\mathscr {P}_t^\dagger \rho )}\;. \end{aligned}$$

In particular, the metric derivative of the heat flow with respect to $\mathscr {W}$ satisfies $\vert {(\mathscr {P}_t^\dagger \rho )'}\vert \le \sqrt{\mathcal {I}_\sigma (\mathscr {P}_t^\dagger \rho )}$.

### Proof

Set $\rho _t:=\mathscr {P}_t^\dagger \rho$. Using the triangle inequality for $\mathscr {W}$ we obtain

$$\begin{aligned} \frac{\mathrm {d}^+}{\mathrm {d}t}\mathscr {W}(\rho _t,\nu )&= \limsup _{s\downarrow 0}\frac{1}{s}\big (\mathscr {W}(\rho _{t+s},\nu )-\mathscr {W}(\rho _t,\nu )\big ) \\&\le \limsup _{s\downarrow 0} \frac{1}{s}\mathscr {W}(\rho _t,\rho _{t+s})\ . \end{aligned}$$

In view of the gradient flow identity $\partial _t \rho = {{\,\mathrm{div}\,}}({\widehat{\rho }}\#\nabla (\log \rho - \log \sigma ) )$, the definition of $\mathscr {W}$ yields

$$\begin{aligned} \limsup _{s\downarrow 0}\frac{1}{s} \mathscr {W}(\rho _t,\rho _{t+s})&\le \limsup _{s\downarrow 0}\frac{1}{s}\int _t^{t+s} \Vert \nabla ( \log \rho _r - \log \sigma ) \Vert _{\rho _r} \; \mathrm {d}r \\&= \limsup _{s\downarrow 0}\frac{1}{s}\int \limits _t^{t+s}\sqrt{\mathcal {I}_\sigma (\rho _r)}\; \mathrm {d}r \\&= \sqrt{\mathcal {I}_\sigma (\rho _t)}\ . \end{aligned}$$

The last equality follows from the continuity of $r\mapsto \sqrt{\mathcal {I}_\sigma (\rho _r)}$. $\square$

The following result is a non-commutative analogue of a well-known result by Otto and Villani [39].

### Theorem 11.3

Assume that ${{\,\mathrm{Ric}\,}}(\mathcal {A},\nabla ,\sigma )\ge \kappa$ for some $\kappa \in {{\mathbb {R}}}$. Then ${{\,\mathrm{H{\mathscr {W}}I}\,}}(\kappa )$ holds as well.

### Proof

Fix $\rho \in {{\mathfrak {P}}}$. If $\mathcal {I}_\sigma (\rho )=+\infty$ there is nothing to prove, so we will assume without loss of generality that $\rho \in {{\mathfrak {P}}}_+$. Set $\rho _t :=\mathscr {P}_t^\dagger \rho$. From Theorem 10.2 and the lower bound on the Ricci curvature we know that the curve $(\rho _t)$ satisfies EVI($\kappa$), i.e., equation (10.2). Choosing $\nu =\sigma$ and $t = 0$ in the EVI($\kappa$) yields

$$\begin{aligned} {{\,\mathrm{Ent}\,}}_\sigma (\rho ) \le -\frac{1}{2} \left. \frac{\mathrm {d}^+}{\mathrm {d}t}\right| _{t=0}\mathscr {W}(\rho _t,\sigma )^2 -\frac{\kappa }{2}\mathscr {W}(\rho ,\sigma )^2\ . \end{aligned}$$

It remains to show that

$$\begin{aligned} -\frac{1}{2} \left. \frac{\mathrm {d}^+}{\mathrm {d}t}\right| _{t=0}\mathscr {W}(\rho _t,\sigma )^2~\le ~\mathscr {W}(\rho ,\sigma ) \sqrt{\mathcal {I}_\sigma (\rho )}\ . \end{aligned}$$

To see this, we use the triangle inequality to estimate

$$\begin{aligned} -\frac{1}{2} \left. \frac{\mathrm {d}^+}{\mathrm {d}t}\right| _{t=0}\mathscr {W}(\rho _t,\sigma )^2&= \liminf _{t\downarrow 0}\frac{1}{2t} \left( \mathscr {W}(\rho ,\sigma )^2-\mathscr {W}(\rho _{t},\sigma )^2\right) \\ {}&\le \limsup _{t\downarrow 0}\frac{1}{2t} \left( \mathscr {W}(\rho ,\rho _t)^2 + 2\mathscr {W}(\rho ,\rho _t)\cdot \mathscr {W}(\rho ,\sigma )\right) \ , \end{aligned}$$

Using Proposition 11.2 with $\nu = \rho$ and $t=0$ we see that the second term on the right-hand side is bounded by $\mathscr {W}(\rho ,\sigma ) \sqrt{\mathcal {I}_\sigma (\rho )}$, while the first term vanishes. $\square$

The following result is now a simple consequence.

### Theorem 11.4

(Quantum Bakry–Émery Theorem) Suppose that ${{\,\mathrm{Ric}\,}}(\mathcal {A},\sigma ,\nabla )\ge \lambda$ for some $\lambda >0$. Then the modified logarithmic Sobolev inequality ${{\,\mathrm{MLSI}\,}}(\lambda )$ holds.

### Proof

Take $\rho \in {{\mathfrak {P}}}_+$. It follows from Theorem 11.3 that $(\mathcal {A},\sigma ,\nabla )$ satisfies ${{\,\mathrm{H{\mathscr {W}}I}\,}}(\lambda )$. Using this inequality followed by Young’s inequality we obtain

$$\begin{aligned} {{\,\mathrm{Ent}\,}}_\sigma (\rho ) \le \mathscr {W}(\rho ,\sigma ) \sqrt{\mathcal {I}_\sigma (\rho )} - \frac{\lambda }{2} \mathscr {W}(\rho ,\sigma )^2 \le \frac{1}{2\lambda }\mathcal {I}_\sigma (\rho ) \ , \end{aligned}$$

which is $${{\,\mathrm{MLSI}\,}}(\lambda )$$. $$\square $$

### Theorem 11.5

(Quantum Otto–Villani Theorem) Suppose that the differential structure $(\mathcal {A},\nabla ,\sigma )$ satisfies ${{\,\mathrm{MLSI}\,}}(\lambda )$ for some $\lambda >0$. Then the Talagrand inequality ${{\,\mathrm{T_\mathscr {W}}\,}}(\lambda )$ holds as well.

### Proof

It suffices to prove ${{\,\mathrm{T_\mathscr {W}}\,}}(\lambda )$ for $\rho \in {{\mathfrak {P}}}_+$, since the inequality for general $\rho \in {{\mathfrak {P}}}$ can then be obtained by approximation.

Fix $\rho \in {{\mathfrak {P}}}_+$ and set $\rho _t = \mathscr {P}_t^\dagger \rho$. As $t\rightarrow \infty$, we use (11.1) to infer that

11.3 $$\begin{aligned} {{\,\mathrm{Ent}\,}}_\sigma (\rho _t)\rightarrow 0 \quad \text{ and } \quad \mathscr {W}(\rho ,\rho _t)\rightarrow \mathscr {W}(\rho ,\sigma )\;. \end{aligned}$$

Define $$F:{{\mathbb {R}}}_+\rightarrow {{\mathbb {R}}}_+$$ by

$$\begin{aligned} F(t) := \mathscr {W}(\rho ,\rho _t) + \sqrt{\frac{2}{\lambda }{{\,\mathrm{Ent}\,}}_\sigma (\rho _t)}\;. \end{aligned}$$

We have $F(0) = \sqrt{\frac{2}{\lambda }{{\,\mathrm{Ent}\,}}_\sigma (\rho )}$ and $F(t)\rightarrow \mathscr {W}(\rho ,\sigma )$ as $t\rightarrow \infty$ by (11.3). Hence it is sufficient to show that $\frac{\mathrm {d}^+}{\mathrm {d}t}F(t) \le 0$ for all $t \ge 0$. If $\rho _t\ne \sigma$, we use Proposition 11.2 and the identity $\frac{\mathrm {d}}{\mathrm {d}t}{{\,\mathrm{Ent}\,}}_\sigma (\rho _t) = - \mathcal {I}_\sigma (\rho _t)$ to obtain

$$\begin{aligned} \frac{\mathrm {d}^+}{\mathrm {d}t}F(t) \le \sqrt{\mathcal {I}_\sigma (\rho _t)} - \frac{\mathcal {I}_\sigma (\rho _t)}{\sqrt{2\lambda {{\,\mathrm{Ent}\,}}_\sigma (\rho _t)}} \le 0\ , \end{aligned}$$

where the last inequality follows from ${{\,\mathrm{MLSI}\,}}(\lambda )$. If $\rho _t = \sigma$, then the same inequality holds, since this implies that $\rho _r = \sigma$ for all $r\ge t$. $\square$

It is known that the modified logarithmic Sobolev inequality implies a Poincaré inequality by a linearization argument. The following result shows that Poincaré inequality is in fact implied by the Talagrand inequality, which is weaker than the MLSI in view of the previous theorem. The BKM metric in the left-hand side of P($\lambda$) appears since it also appears in the second order expansion of the relative entropy of ${{\,\mathrm{Ent}\,}}_\sigma (\rho )$ around $\rho = \sigma$; see (6.12).

### Proposition 11.6

Assume that the triple $(\mathcal {A},\sigma ,\nabla )$ satisfies T$_{\mathscr {W}}$($\lambda$) for some $\lambda >0$. Then the Poincaré inequality P($\lambda$) and the $T_1$-transport inequality T$_{1}$($\lambda$) hold as well. Moreover, ${{\,\mathrm{Ric}\,}}(\mathcal {A},\sigma ,\nabla ) \ge \lambda$ implies ${{\,\mathrm{P}\,}}(\lambda )$.

### Proof

The fact that T$_{\mathscr {W}}$($\lambda$) implies the $T_1$-inequality is an immediate consequence of Proposition 9.4.

Suppose that T$_{\mathscr {W}}$($\lambda$) holds and let us show ${{\,\mathrm{P}\,}}(\lambda )$. Fix $\nu \in \mathcal {A}_0$ and set $\rho ^\varepsilon : = \sigma + \varepsilon \nu$. Then $\rho ^\varepsilon \in {{\mathfrak {P}}}_+$ for sufficiently small $\varepsilon >0$. For such $\varepsilon > 0$, let $(\rho _t^\varepsilon , \mathbf {B}_t^\varepsilon )_t$ be an action minimizing curve connecting $\rho _0^\varepsilon = \rho ^\varepsilon$ and $\rho _1^\varepsilon = \sigma$. Thus we have $\partial _t \rho _t^\varepsilon + {{\,\mathrm{div}\,}}({\widehat{\rho }}_t^\varepsilon \# \mathbf {B}_t^\varepsilon ) = 0$ and $\int _0^1 \tau [(\mathbf {B}_t^\varepsilon )^*{\widehat{\rho }}_t^\varepsilon \# \mathbf {B}_t^\varepsilon ] \; \mathrm {d}t = \mathscr {W}(\rho ^\varepsilon , \sigma )^2$.

Write $A = \int _0^\infty (x + \sigma )^{-1} \nu (x + \sigma )^{-1} \; \mathrm {d}x$ so that $\nu = \int _0^1 \sigma ^{1-s} A \sigma ^s \; \mathrm {d}s$. Using the continuity equation we obtain

$$\begin{aligned} \Vert A\Vert _{L^2_\mathrm{BKM}(\sigma )}^2&= \frac{1}{\varepsilon } \tau [A^* (\rho ^\varepsilon - \sigma ) ] = \frac{1}{\varepsilon } \tau [A^* {{\,\mathrm{div}\,}}({\widehat{\rho }}_t^\varepsilon \# \mathbf {B}_t^\varepsilon ) ] = -\frac{1}{\varepsilon } \int _0^1 \tau [ (\nabla A)^* {\widehat{\rho }}_t^\varepsilon \# \mathbf {B}_t^\varepsilon ] \; \mathrm {d}t\ . \end{aligned}$$

The Cauchy-Schwarz inequality yields

$$\begin{aligned} \Vert A\Vert _{L^2_\mathrm{BKM}(\sigma )}^2&\le \frac{1}{\varepsilon } \bigg ( \int _0^1 \Vert \nabla A \Vert _{\rho _t^\varepsilon }^2 \; \mathrm {d}t \bigg )^{1/2} \bigg ( \int _0^1 \Vert \mathbf {B}_t^\varepsilon \Vert _{\rho _t^\varepsilon }^2 \; \mathrm {d}t \bigg )^{1/2} \\&= \frac{1}{\varepsilon } \bigg ( \int _0^1 \Vert \nabla A \Vert _{\rho _t^\varepsilon }^2 \; \mathrm {d}t \bigg )^{1/2} \mathscr {W}(\rho ^\varepsilon ,\sigma ) \ , \end{aligned}$$

since $(\rho _t^\varepsilon )_t$ is a $\mathscr {W}$-geodesic. Using ${{\,\mathrm{T_\mathscr {W}}\,}}(\lambda )$ we obtain

$$\begin{aligned} \limsup _{\varepsilon \rightarrow 0} \frac{\mathscr {W}(\rho ^\varepsilon ,\sigma )}{\varepsilon }&\le \limsup _{\varepsilon \rightarrow 0} \frac{1}{\varepsilon }\sqrt{\frac{2}{\lambda }{{\,\mathrm{Ent}\,}}_\sigma (\rho ^\varepsilon )} \le \frac{1}{\sqrt{\lambda }} \Vert A\Vert _{L^2_\mathrm{BKM}(\sigma )}\ , \end{aligned}$$

since ${{\,\mathrm{Ent}\,}}_\sigma (\rho ^\varepsilon ) = \frac{1}{2} \varepsilon ^2 \Vert A\Vert _{L^2_\mathrm{BKM}(\sigma )}^2 + o(\varepsilon ^2)$ by (6.11) and (6.12). It remains to show that, as $\varepsilon \rightarrow 0$,

$$\begin{aligned} \int _0^1 \Vert \nabla A \Vert _{\rho _t^\varepsilon }^2 \; \mathrm {d}t \rightarrow \Vert \nabla A \Vert _{\sigma }^2 \ . \end{aligned}$$

To see this, note that $\tau [|\rho ^\varepsilon - \sigma |] \rightarrow 0$, hence $\mathscr {W}(\rho ^\varepsilon , \sigma ) \rightarrow 0$. Since $\mathscr {W}(\rho _t^\varepsilon , \sigma ) = (1-t) \mathscr {W}(\rho ^\varepsilon , \sigma )$, it follows that $\mathscr {W}(\rho _t^\varepsilon , \sigma ) \rightarrow 0$ as $\varepsilon \rightarrow 0$ for all $t \in [0,1]$, which implies that $\Vert \nabla A \Vert _{\rho _t^\varepsilon }^2 \rightarrow \Vert \nabla A \Vert _{\sigma }^2$ for all $t \in [0,1]$. The result now follows using dominated convergence, since $\Vert \nabla A \Vert _{\rho _t^\varepsilon }^2 \le \Vert \nabla A\Vert _\mathcal {B}$ by Lemma 9.3.

The final assertion of the proposition follows by combining this result with Theorem 11.4 and Theorem 11.5. $\square$
